# Rad51 Paralogs Remodel Pre-synaptic Rad51 Filaments to Stimulate Homologous Recombination

**DOI:** 10.1016/j.cell.2015.06.015

**Published:** 2015-07-16

**Authors:** Martin R.G. Taylor, Mário Špírek, Kathy R. Chaurasiya, Jordan D. Ward, Raffaella Carzaniga, Xiong Yu, Edward H. Egelman, Lucy M. Collinson, David Rueda, Lumir Krejci, Simon J. Boulton

**Affiliations:** 1 DNA Damage Response Laboratory, Clare Hall Laboratory, The Francis Crick Institute, South Mimms EN6 3LD, UK; 2 Department of Biology, Masaryk University, 62500 Brno, Czech Republic; 3 International Clinical Research Center, St. Anne’s University Hospital in Brno, 62500 Brno, Czech Republic; 4 National Centre for Biomolecular Research, Masaryk University, 62500 Brno, Czech Republic; 5 Section of Virology, Single Molecule Imaging Group and MRC Clinical Sciences Centre, Department of Medicine, Imperial College London, London W12 0NN, UK; 6 UCSF-Mission Bay, Genentech Hall S574, San Francisco, CA 94158, USA; 7 Electron Microscopy Science Technology Platform, Lincoln’s Inn Fields Laboratory, The Francis Crick Institute, London WC2A 3LY, UK; 8 Department of Biochemistry and Molecular Genetics, University of Virginia School of Medicine, Charlottesville, VA 22908, USA

## Abstract

Repair of DNA double strand breaks by homologous recombination (HR) is initiated by Rad51 filament nucleation on single-stranded DNA (ssDNA), which catalyzes strand exchange with homologous duplex DNA. BRCA2 and the Rad51 paralogs are tumor suppressors and critical mediators of Rad51. To gain insight into Rad51 paralog function, we investigated a heterodimeric Rad51 paralog complex, RFS-1/RIP-1, and uncovered the molecular basis by which Rad51 paralogs promote HR. Unlike BRCA2, which nucleates RAD-51-ssDNA filaments, RFS-1/RIP-1 binds and remodels pre-synaptic filaments to a stabilized, “open,” and flexible conformation, in which the ssDNA is more accessible to nuclease digestion and RAD-51 dissociation rate is reduced. Walker box mutations in RFS-1, which abolish filament remodeling, fail to stimulate RAD-51 strand exchange activity, demonstrating that remodeling is essential for RFS-1/RIP-1 function. We propose that Rad51 paralogs stimulate HR by remodeling the Rad51 filament, priming it for strand exchange with the template duplex.

## Highlights

• A Rad51 paralog complex, RFS-1/RIP-1, binds RAD-51-ssDNA pre-synaptic filaments
• RFS-1/RIP-1 stabilizes the filament by reducing RAD-51 dissociation from ssDNA
• RFS-1/RIP-1 remodels the pre-synaptic complex to a more “open,” flexible conformation
• Pre-synaptic complex remodeling stimulates strand exchange and recombination

## Graphical Abstract

**Figure:**
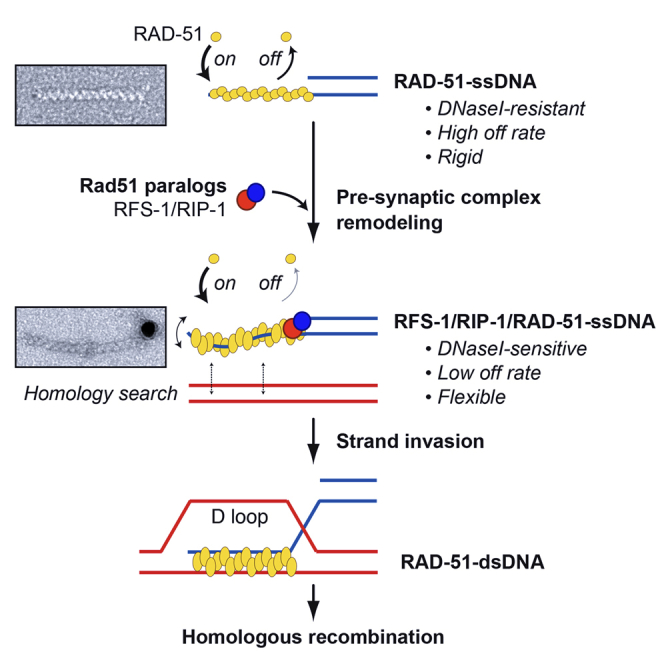

## Teaser

Rad51 paralogs, known tumor suppressors, induce remodeling at the tips of Rad51-ssDNA filaments to form an open and flexible conformation that promotes homologous recombination.

## Introduction

Homologous recombination (HR) is an essential mechanism for the repair of DNA double strand breaks (DSBs) and damaged replication forks. HR is initiated at single-stranded DNA (ssDNA) exposed at nucleolytically processed DSB ends or post-replicative ssDNA gaps by the exchange of the ssDNA binding protein RPA for the recombinase enzyme Rad51, which forms helical nucleoprotein filaments on ssDNA. Rad51-ssDNA filaments probe for homologous duplex DNA and catalyze strand invasion, displacing the non-complementary strand of the template duplex to form a displacement loop (D loop) structure. Unloading of Rad51 from double-stranded DNA (dsDNA) permits the initiation of repair DNA synthesis and the resulting joint molecules are processed by various enzymes to complete the repair reaction (Chapman et al., 2012; San Filippo et al., 2008).

HR is regulated by mediator proteins, including BRCA2, Rad54, and the family of Rad51 paralogs (San Filippo et al., 2008), which appear to act as positive regulators at different steps of the HR reaction. For example, BRCA2 facilitates Rad51 nuclear localization (Jeyasekharan et al., 2013; Martin et al., 2005), RPA displacement from ssDNA, and Rad51 filament nucleation (Jensen et al., 2010; Liu et al., 2010; Thorslund et al., 2010), whereas Rad54 unloads Rad51 from dsDNA to promote repair DNA synthesis (Solinger et al., 2002). However, the molecular mechanism underlying the stimulation of HR by the Rad51 paralogs has remained elusive.

Rad51 paralog proteins share sequence similarity to Rad51 and possess homology extending across the motor ATPase fold, including the highly conserved Walker A and Walker B boxes, but not the N-terminal helix-hairpin-helix motif (Lin et al., 2006). Five Rad51 paralogs have been identified in mammalian and avian species, which interact with one another to form two constitutive complexes, RAD51B-RAD51C-RAD51D-XRCC2 (BCDX2) and RAD51C-XRCC3 (CX3) (Masson et al., 2001; Yonetani et al., 2005), and in budding yeast two Rad51 paralogs constitute the Rad55-Rad57 heterodimer (Sung, 1997). In addition, the budding and fission yeast Shu complexes (Martín et al., 2006; Shor et al., 2005) contain proteins lacking detectable sequence homology to Rad51 but contain Rad51-like folds as determined by crystal structures (Sasanuma et al., 2013; Tao et al., 2012) or show very limited homology centered on the Walker B motif, such as budding/fission yeast Psy3/Rdl1 (Martín et al., 2006). The high degree of conservation around these short Walker motifs among the Rad51 paralog family reflects their functional importance in conferring resistance to DNA damage and the integrity of the Rad51 paralog complexes in mammalian cells (French et al., 2003; Gruver et al., 2005; Wiese et al., 2006; Yamada et al., 2004).

Ablation of Rad51 paralogs leads to severe HR defects, DNA damage sensitivity, chromosome abnormalities, and defective Rad51 nuclear focus formation after DNA damage, suggestive of a major function at an early stage in the HR reaction (Chun et al., 2013; French et al., 2002; Gasior et al., 1998; Godthelp et al., 2002; Hays et al., 1995; Johnson et al., 1999; Pierce et al., 1999; Rattray and Symington, 1995; Takata et al., 2000, 2001). Like *BRCA2* and *PALB2*, which are mutated in Fanconi anemia and breast and ovarian cancer (Howlett et al., 2002; Lancaster et al., 1996; Rahman et al., 2007; Reid et al., 2007; Wooster et al., 1995; Xia et al., 2007), biallelic germline mutations in *RAD51C* cause a severe form of Fanconi anemia (Vaz et al., 2010), whereas monoallelic inheritance of mutations in *RAD51C* and *RAD51D*, and *RAD51B*, predispose individuals to ovarian and breast cancer, respectively (Golmard et al., 2013; Loveday et al., 2011; Meindl et al., 2010), demonstrating an important tumor suppressor function for HR mediators.

The budding yeast Rad55-Rad57 complex (Sung, 1997) and a sub-complex of the human BCDX2 complex, RAD51B-RAD51C (Sigurdsson et al., 2001), have been purified as heterodimers and despite lacking intrinsic recombinase activity they stimulate strand exchange by Rad51 in vitro. Rad55-Rad57 and the Shu complex form co-complexes with Rad51-ssDNA filaments (Liu et al., 2011a; Sasanuma et al., 2013), which in the case of Rad55-Rad57 renders the pre-synaptic complex resistant to disruption by the anti-recombinase Srs2 (Liu et al., 2011a). However, the mechanism by which Rad51 paralogs directly stimulate the recombinase activity of Rad51 has remained enigmatic for many years (Sung, 1997). Additionally, whether the Rad51 paralogs confer any intrinsic stabilization or alteration in the structural properties of the pre-synaptic complex is unknown, as is the mechanistic importance of their conserved Walker motifs.

In this study, we report the identification and characterization of a Rad51 paralog complex, RFS-1/RIP-1, from *Caenorhabditis elegans*. RFS-1/RIP-1 is required for HR and RAD-51 focus formation at DNA damage sites in vivo, and it stimulates the recombinase activity of RAD-51 and associates directly with RAD-51 filaments in vitro. Using multiple biochemical and biophysical approaches, we demonstrate that RFS-1/RIP-1 structurally remodels the pre-synaptic RAD-51-ssDNA filament to a stabilized, “open,” flexible conformation, which facilitates strand exchange with the template duplex. Using specific mutants in the Walker boxes of RFS-1, which are compromised for stimulating strand exchange, we demonstrate that filament remodeling is critical for RFS-1/RIP-1 mediator activity. Collectively, this defines the underlying mechanism of HR stimulation by Rad51 paralogs.

## Results

### Identification of a Heterodimeric Rad51 Paralog Complex in *C. elegans*

**Figure 1:**
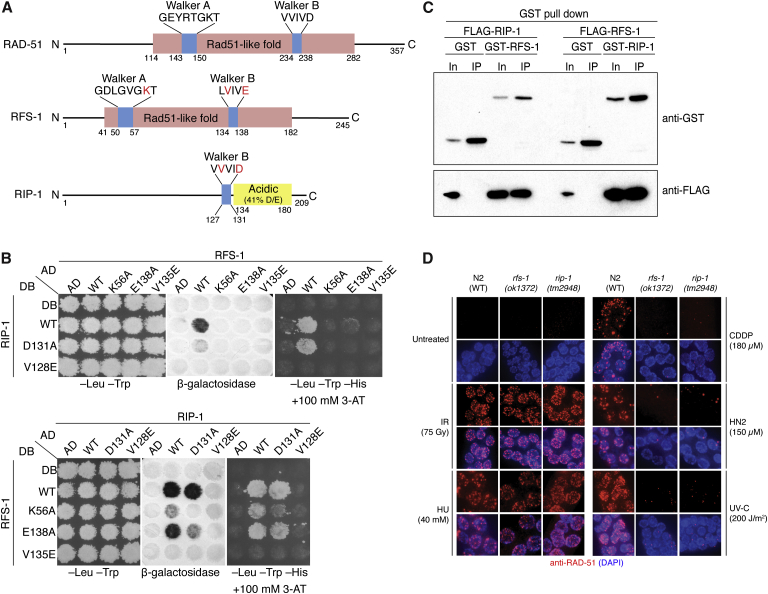
RIP-1 Is a Highly Divergent Rad51 Paralog that Forms a Complex with RFS-1 (A) Schematics of RAD-51, RFS-1, and RIP-1. RAD-51 and RFS-1 are homologous in their central Rad51-like folds (pink). Walker A and B motifs (blue) are annotated, and residues examined by mutagenesis are indicated (red). RIP-1 contains an acidic region (yellow) of unknown function. (B) RFS-1 and RIP-1 interact via ATPase motifs in reciprocal yeast two-hybrid assays, indicated by positive β-galactosidase expression and survival on media containing 3-aminotriazole (3-AT) in the absence of histidine. Growth on media lacking leucine and tryptophan is a positive control for plasmid transfection. (C) GST pull-downs of *C. elegans* RFS-1 and RIP-1 expressed in human 293T cells. In, input. IP, pull-down. (D) RAD-51 immunofluorescence (red) in mitotic nuclei of worm germlines from the indicated genotypes. DNA is stained with DAPI (blue). IR, ionizing radiation. HU, hydroxyurea. CDDP, *cis*-platin. HN2, nitrogen mustard. UV-C, ultraviolet light. See also Figure S1.

**Figure 2:**
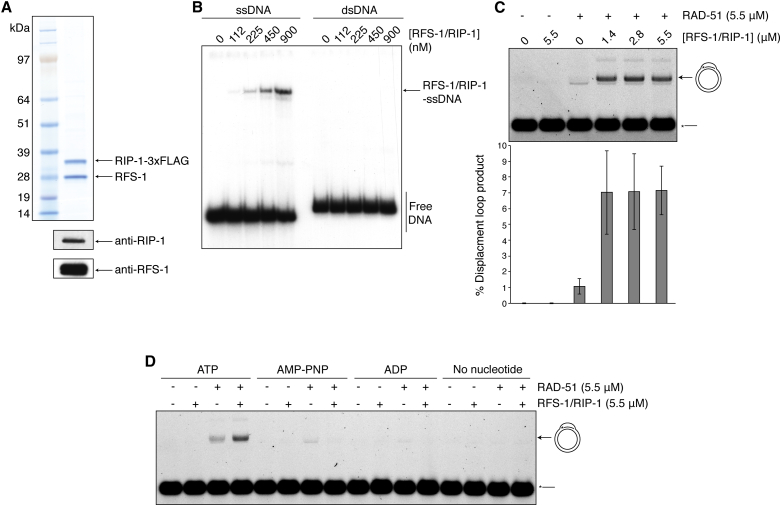
Biochemical Properties of RFS-1/RIP-1 Complex (A) Purification of recombinant RFS-1/RIP-1 from yeast cells by FLAG immunoprecipitation. Western blots confirm the identity of the two bands. (B) EMSA showing RFS-1/RIP-1 binding to ssDNA, but not to dsDNA (60-mer). (C) RFS-1/RIP-1 stimulates D loop formation by RAD-51. Error bars indicate SD (n = 4). (D) RFS-1/RIP-1 mediator activity requires ATP. See also Figure S2.

**Figure S1:**
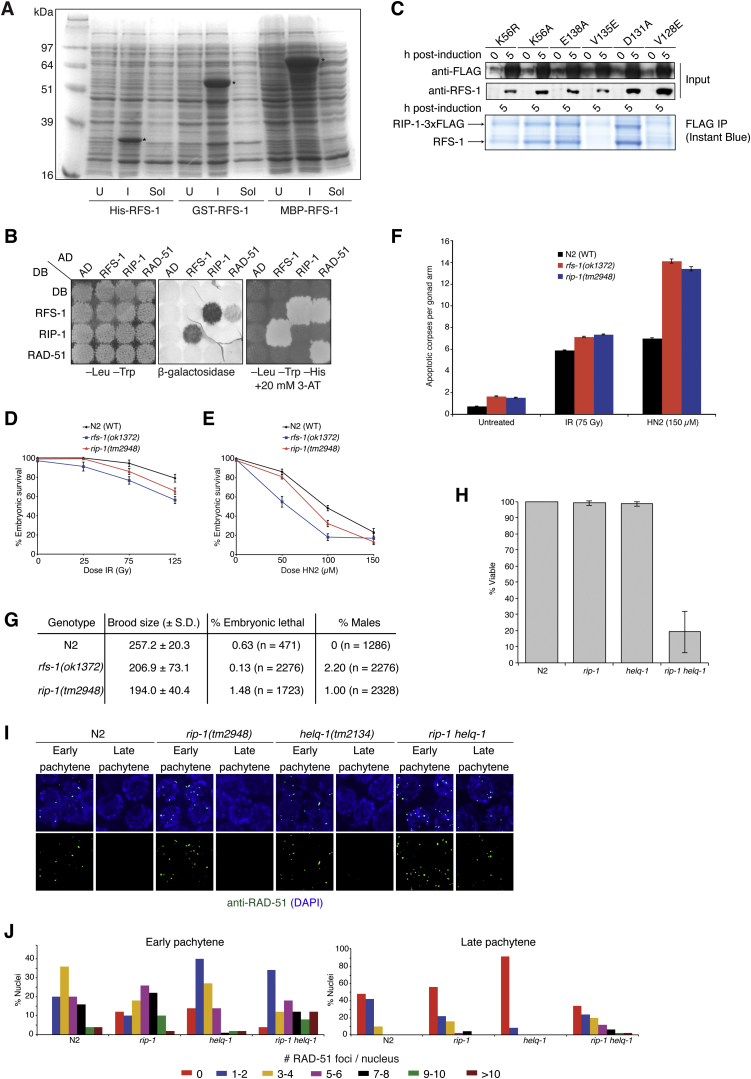
RIP-1 Is a Highly Divergent Rad51 Paralog that Forms a Complex with RFS-1, Related to Figure 1 (A) Expression of N-terminally 6xHis-, GST- or MBP-tagged RFS-1 in *E. coli* fails to produce soluble protein. Proteins were detected by Instant Blue staining. U, uninduced. I, induced. Sol, soluble. Induced RFS-1 fusions are indicated with asterisks. (B) Reciprocal yeast two-hybrid matrix for RFS-1, RIP-1 and RAD-51. Interactions are indicated by positive β-galactosidase expression and survival on media containing 3-aminotriazole (3-AT) in the absence of histidine. Growth on media lacking leucine and tryptophan is a positive control for plasmid transfection. (C) Expression and purification of RFS-1/RIP-1 mutant proteins in yeast. Western blots show whole cell TCA extracts before and 5 hr after 2% galactose induction. FLAG bead eluates from soluble extracts were detected by Instant Blue staining. IP, immunoprecipitate. (D and E) Embryonic survival of progeny of animals treated with ionizing radiation (IR) (D) and nitrogen mustard (HN2) (E). Error bars indicate SEM from at least 24 adults over 2 independent experiments. (F) Number of apoptotic corpses scored by SYTO12 staining in the gonads of animals treated with ionizing radiation (IR) or nitrogen mustard (HN2). Error bars indicate SEM from at least 28 adults over 2 independent experiments. (G) Table of brood size, embryonic lethality and percent males for the indicated genotypes. (H) Embryo viability of all progeny from 5-10 parental worms of the indicated genotype. Error bars indicate SD. (I) RAD-51 immunofluorescence (green) from representative early and late pachytene nuclei of worm germlines from the indicated genotypes. DNA is stained with DAPI (blue). (J) Quantification of (I) indicating the distribution of the number of RAD-51 foci per nucleus in early and late pachytene from each of the indicated genotypes. Foci were counted from 50 nuclei from 5 animals.

To investigate the mechanism of action of Rad51 paralogs in promoting HR, we utilized the simplified model system *C. elegans*, which encodes a single canonical Rad51 paralog, RFS-1 (Figure 1A). We previously showed that *rfs-1* mutant strains are sensitive to DNA damage, defective for RAD-51 focus formation at stalled replication forks, and exhibit meiotic defects when combined with *helq-1* mutations (Ward et al., 2007, 2010). However, attempts to purify RFS-1 yielded largely insoluble protein intractable to biochemical analysis (Figure S1A). Since Rad51 paralogs in other organisms function as complexes, we reasoned that RFS-1 may require a binding partner for optimal function. Using a yeast two-hybrid screen (Boulton et al., 2002), we identified an orphan protein encoded by R01H10.5 (Uniprot ID code Q21621) that we named RIP-1 (RFS-1 interacting protein) (Figure 1A), which interacted with RFS-1 by yeast two-hybrid (Figure 1B), glutathione S-transferase (GST) pull-downs of *C. elegans* RFS-1 and RIP-1 expressed in human cells (Figure 1C), and FLAG co-immunoprecipitation (co-IP) from yeast cells (Figure 2A). Although RFS-1 interacts with RAD-51 in yeast two-hybrid, no interaction between RAD-51 and RIP-1 was detectable (Figure S1B).

RIP-1 bears no obvious sequence homology with Rad51 family proteins, but does contain a sequence resembling a Walker B motif (Figure 1A), similar to the divergent yeast Rad51 paralog Psy3 that adopts a Rad51-like fold (Martín et al., 2006; Sasanuma et al., 2013; Tao et al., 2012). Since Walker B motifs mediate protein-protein interactions in human Rad51 paralog complexes (Wiese et al., 2006), we tested if this dependency is conserved for the RFS-1/RIP-1 protein-protein interaction. Mutation of the second position valine in the Walker B box of either protein (RFS-1 V135E or RIP-1 V128E) was sufficient to completely abolish the interaction in yeast two-hybrid (Figures 1A and 1C), suggesting that the Walker B motifs mediate the RFS-1/RIP-1 interaction interface. Notably, when these mutants were expressed in yeast, FLAG-tagged RIP-1 was expressed but was not detectable after co-IP (Figure S1C), suggesting RFS-1 and RIP-1 are interdependent for their solubility, consistent with the inability to purify RFS-1 in isolation (Figure S1A). In contrast, mutation of other residues, including RFS-1 lysine-56 and glutamate-138 in the Walker A and B boxes, respectively, conferred weakened yeast two-hybrid interactions but were still permissive for co-IP (Figures 1C and S1C), suggestive of abnormal, but intact, protein complexes.

A *rip-1* deletion mutant (*tm2948*) was found to phenocopy *rfs-1* mutants. Like *rfs-1* mutants, *rip-1*-deficient strains are defective for RAD-51 focus formation after treatment with DNA-damaging agents that stall replication forks (Figure 1D), are sensitive to DNA damage (Figures S1D and S1E), and display elevated germline apoptosis after treatment with interstrand crosslinking agents (Figure S1F) (Ward et al., 2007). *rip-1* mutants also phenocopy the meiotic HR defects of *rfs-1* strains (Ward et al., 2010), exhibiting elevated frequencies of males (Figure S1G) and synthetic lethality with *helq-1*, which is associated with persistent meiotic RAD-51 foci (Figures S1H–S1J). These observations suggest that RIP-1 likely represents a highly divergent RAD-51 paralog that functions as a complex with RFS-1. The existence of non-canonical Rad51 paralogs in other organisms is not unprecedented: yeast cells encode the Shu complex (Martín et al., 2006; Shor et al., 2005), which functions in HR, and human cells encode an ATPase-sharing homology with the archael recombinase RadA, SWSAP1, required for HR, Rad51 focus formation, and DNA damage resistance (Liu et al., 2011b).

### RFS-1/RIP-1 Binds ssDNA and Stimulates RAD-51 Recombinase Activity

**Figure S2:**
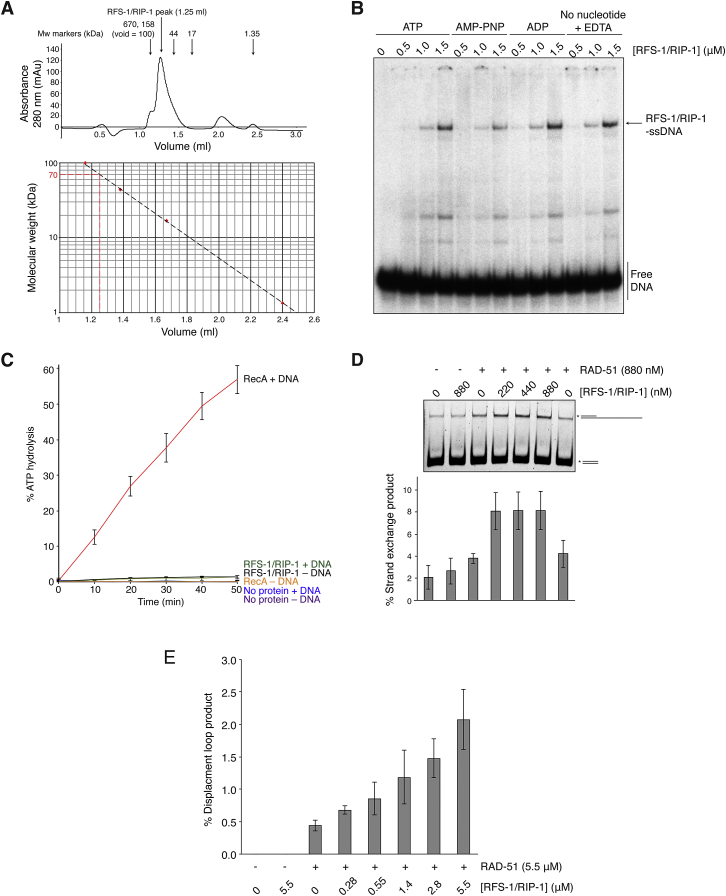
Biochemical Properties of RFS-1/RIP-1 Complex, Related to Figure 2 (A) Size exclusion chromatography trace from a 3 ml Superdex 75 5/150 GL column loaded with 30 μl RFS-1/RIP-1 from single-step FLAG immunoprecipitation. Arrows indicate molecular weight (Mw) markers. A plot of the log_10_(Mw) against elution volume for the markers allowed estimation of a size for the complex. (B) EMSA showing RFS-1/RIP-1 ssDNA binding is nucleotide independent. (C) RFS-1/RIP-1 lacks detectable ATPase activity in the presence or absence of DNA. Error bars indicate SD (n = 3). RecA + DNA is a positive control. (D) RFS-1/RIP-1 stimulates strand exchange by RAD-51. Error bars indicate SD (n = 4). (E) RFS-1/RIP-1 stimulates D loop formation by RAD-51 at the same RAD-51:DNA ratio as used in stopped flow experiments. Error bars indicate SD (n = 3).

To investigate the biochemical properties of RFS-1/RIP-1, we co-purified recombinant proteins from budding yeast cells (Figure 2A), which migrated with a molecular weight of approximately 70 kDa on size-exclusion chromatography (Figure S2A), consistent with a 1:1 heterodimer. Electrophoretic mobility shift assays (EMSA) demonstrated that RFS-1/RIP-1 weakly binds ssDNA, but not dsDNA, in a nucleotide-independent manner to form a discrete slow-migrating protein-DNA complex in native polyacrylamide gels (Figures 2B and S2B). Although RFS-1/RIP-1 contains Walker motifs, there was little detectable ATPase activity over the background, suggesting that the RFS-1/RIP-1 complex may lack intrinsic ATPase activity (Figure S2C). However, it remains possible that the optimal biochemical condition and/or the appropriate substrate for RFS-1/RIP-1 ATPase activity are yet to be identified.

To assess how RFS-1/RIP-1 might regulate the HR reaction, we performed strand exchange and D loop formation assays. The nematode RAD-51 is an extremely weak recombinase in the absence of mediators even when tested at a wide range of protein-to-DNA ratios (Petalcorin et al., 2006). Nevertheless, addition of sub-stoichiometric concentrations of RFS-1/RIP-1 relative to RAD-51 caused a dramatic stimulation of both D loop formation (Figure 2C) and strand exchange activities (Figure S2D), which was dependent on the presence of the nucleotide co-factor ATP (Figure 2D).

### RFS-1/RIP-1 Binds to RAD-51-ssDNA Filaments

**Figure 3:**
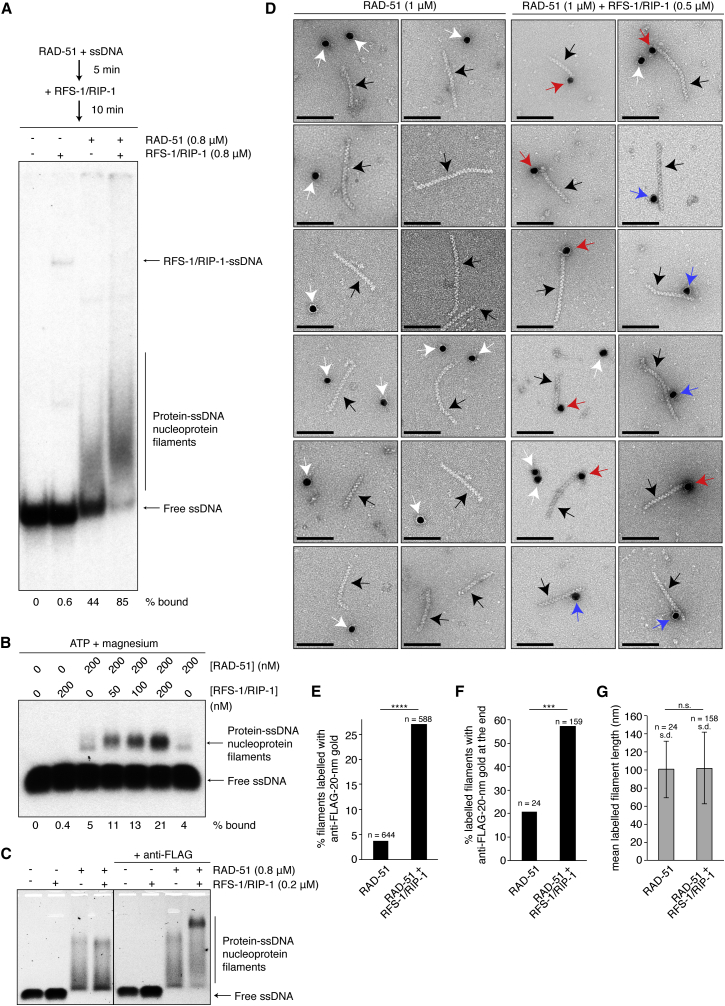
RFS-1/RIP-1 Binds and Modulates the Properties of RAD-51-ssDNA FILAMENTS (A) Protein-DNA complexes formed by RAD-51 and RFS-1/RIP-1 on 60-mer ssDNA with ATP according to the mixing scheme indicated resolved by native PAGE. (B) Proteins were pre-incubated before addition of 60-mer ssDNA, and then protein-DNA complexes were crosslinked and resolved in agarose gels. (C) Immuno-shift analysis of native protein-DNA complexes formed by RAD-51 and RFS-1/RIP-1 on 60-mer ssDNA, followed by incubation with anti-FLAG antibodies and resolution by agarose gel electrophoresis. Black line: cropped superfluous gel lanes. (D) Anti-FLAG-20-nm immuno-gold EM analysis of RAD-51-ssDNA filaments (black arrows) incubated with RFS-1/RIP-1. Scale bar, 100 nm. White arrows: unbound gold; red arrows: gold bound to filament ends; blue arrows: gold bound to filament body. (E) Anti-FLAG-20-nm gold binding is specifically enriched on filaments co-incubated with RFS-1/RIP-1 (Fisher’s exact test; p < 0.0001). (F) Anti-FLAG-20-nm gold is enriched at gold-bound filament ends in the presence of RFS-1/RIP-1 (Fisher’s exact test; p = 0.0009). (G) Gold-bound filament length is not significantly different in the presence or absence of RFS-1/RIP-1 (unpaired two-tailed t test; p = 0.8405). Error bars indicate SD. See also Figure S3.

**Figure S3:**
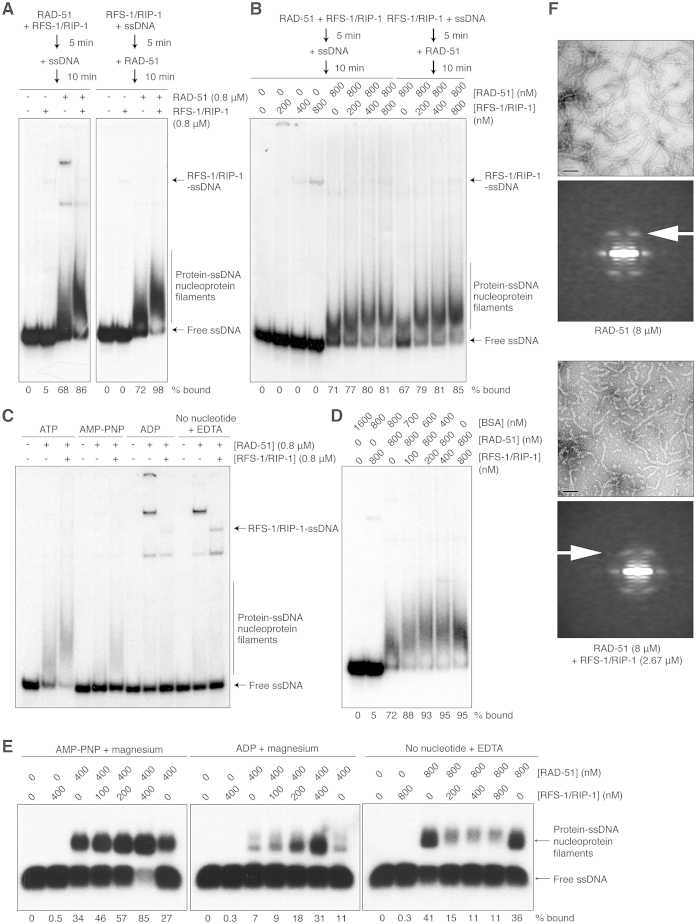
RFS-1/RIP-1 Binds and Modulates the Biochemical Properties of RAD-51-ssDNA Filaments, Related to Figure 3 (A) Protein-DNA complexes formed by RAD-51 and RFS-1/RIP-1 on ssDNA with ATP according to the mixing scheme indicated resolved by native PAGE. (B) Protein-DNA complexes formed by RAD-51 and different concentrations of RFS-1/RIP-1 on ssDNA with ATP according to the mixing scheme indicated resolved by native PAGE. (C) RAD-51 and RFS-1/RIP-1 were pre-incubated before addition of ssDNA with the indicated nucleotide, then protein-DNA complexes were resolved by native PAGE. (D) The effect of RFS-1/RIP-1 on RAD-51-ssDNA filaments is specific and not due to changes in total protein level. RAD-51, RFS-1/RIP-1 and BSA were pre-incubated before addition of ssDNA with the indicated nucleotide, then protein-DNA complexes were resolved by native PAGE. (E) Protein-DNA complexes formed by RAD-51 and RFS-1/RIP-1 on ssDNA with AMP-PNP, ADP or in the absence of nucleotide crosslinked with 0.25% glutaraldehyde and resolved by agarose gel electrophoresis. (F) Electron micrographs (top) and averaged power spectra (bottom) of RAD-51-ssDNA filaments formed with and without RFS-1/RIP-1 used for attempted filament reconstructions. In the averaged power spectra, the arrows show that the layer line arising from the 1-start helix has the same spacing with and without RFS-1/RIP-1, and the average pitch in both cases is ∼90 Å.

To investigate the mechanism by which RFS-1/RIP-1 stimulates RAD-51 recombinase activity, we first assessed how RFS-1/RIP-1 influences RAD-51-ssDNA filament properties in native polyacrylamide gels. RAD-51-ssDNA complexes resolve as fast-migrating smears, representing filaments (Figure 3A) that were only clearly observed in the presence of ATP, and not in the presence of ATPγS, AMP-PNP, or ADP or in the absence of nucleotide (Figure S3C; data not shown). When RAD-51-ssDNA filaments were co-incubated with RFS-1/RIP-1, the resulting nucleoprotein complex migrated more slowly with a concomitant reduction in the amount of free unbound ssDNA, above the additive value predicted from mixing the two proteins (Figure 3A). The effects observed were independent of the relative order of incubation of the protein and DNA components (Figure S3A), occurred at sub-stoichiometric quantities of RFS-1/RIP-1 (Figure S3B), and were specific to RFS-1/RIP-1 (Figure S3D).

We also assessed protein-DNA complexes resolved in agarose gels after glutaraldehyde crosslinking, and this permitted detection of RAD-51-ssDNA filaments in the presence of ATP, ADP, or AMP-PNP and in the absence of nucleotide or magnesium ions (Figures 3B and S3E). RFS-1/RIP-1 caused a greater than additive increase in the proportion of protein-DNA complexes in the presence of any nucleotide. In contrast, the opposite effect was observed in the absence of nucleotide and magnesium, where RFS-1/RIP-1 reduced the proportion of protein-DNA complexes at equilibrium, indicating that the effect of RFS-1/RIP-1 is nucleotide dependent.

Upon co-incubation with RAD-51, we noted that the discrete RFS-1/RIP-1-ssDNA species observed in polyacrylamide gels (Figures 3A, S3A, and S3B) were diminished, suggesting RFS-1/RIP-1 may associate with the filaments. Indeed, retardation of the mobility of the RAD-51-ssDNA filaments formed in the presence of RFS-1/RIP-1 occurred when co-incubated with anti-FLAG antibodies, which bind the FLAG epitope at the RIP-1 C terminus (Figure 3C). Additionally, RFS-1/RIP-1 association with the RAD-51-ssDNA filaments was directly observed using electron microscopy (EM), by incubating with anti-FLAG antibodies conjugated to 20-nm gold particles (Figure 3D). Gold particle binding to the filament was specifically observed in the presence of RFS-1/RIP-1 (Figures 3D and 3E) and was enriched at the filament ends (Figure 3F), suggesting RFS-1/RIP-1 may preferentially cap filaments. No significant differences in filament length were observed among gold particle-bound filaments (RAD-51: 100 ± 31 nm; RAD-51 + RFS-1/RIP-1: 102 ± 40 nm) (Figure 3G), suggesting RFS-1/RIP-1 does not modulate filament extension or disassembly. These data suggest that RFS-1/RIP-1 physically associates with RAD-51-ssDNA filaments, similar to the budding yeast Rad55-Rad57 and Shu complexes (Liu et al., 2011a; Sasanuma et al., 2013).

### Stopped-Flow Measurements Reveal Rapid RAD-51-ssDNA Filament Formation in Real Time

**Figure 4:**
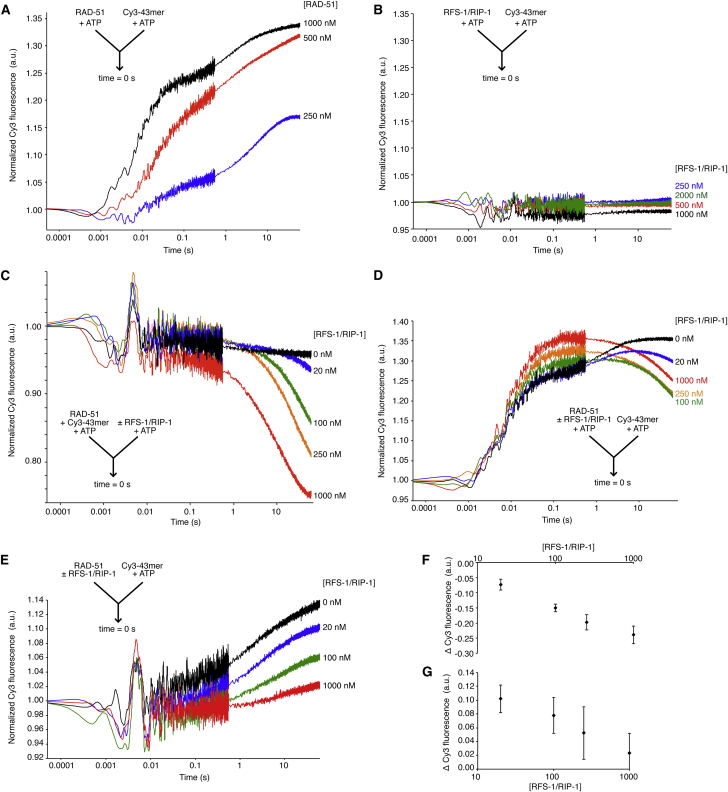
RFS-1/RIP-1 Remodels the RAD-51-ssDNA Filaments (A–G) Analysis of average normalized Cy3-43-mer fluorescence (see the Experimental Procedures for details) plotted as a function of log_10_(time). The arrow (inset) indicates the components of the two syringes rapidly mixed at the 0 s time point in a stopped-flow instrument. (A) Indicated concentrations of RAD-51 mixed with 15 nM Cy3-43-mer (both +ATP) (n = 8–9). (B) Indicated concentrations of RFS-1/RIP-1 mixed with 15 nM Cy3-43-mer (both +ATP) (n = 5–6). (C) RAD-51-ssDNA filaments pre-formed with 1 μM RAD-51 + 15 nM Cy3-43-mer for 10 min and then mixed with the indicated concentrations of RFS-1/RIP-1 (both +ATP) (n = 6–9). (D) 1 μM RAD-51 pre-incubated with indicated concentrations of RFS-1/RIP-1 for 10 min and then mixed with 15 nM Cy3-43-mer (both +ATP) (n = 5–7). (E) As in (D), except proteins were not pre-incubated with ATP (n = 6–9). (F and G) Graphs of average Δ Cy3 fluorescence as a function of RFS-1/RIP-1 concentration for the data presented in (C) and (E), respectively. Error bars indicate SD. See also Figure S4.

**Figure S4:**
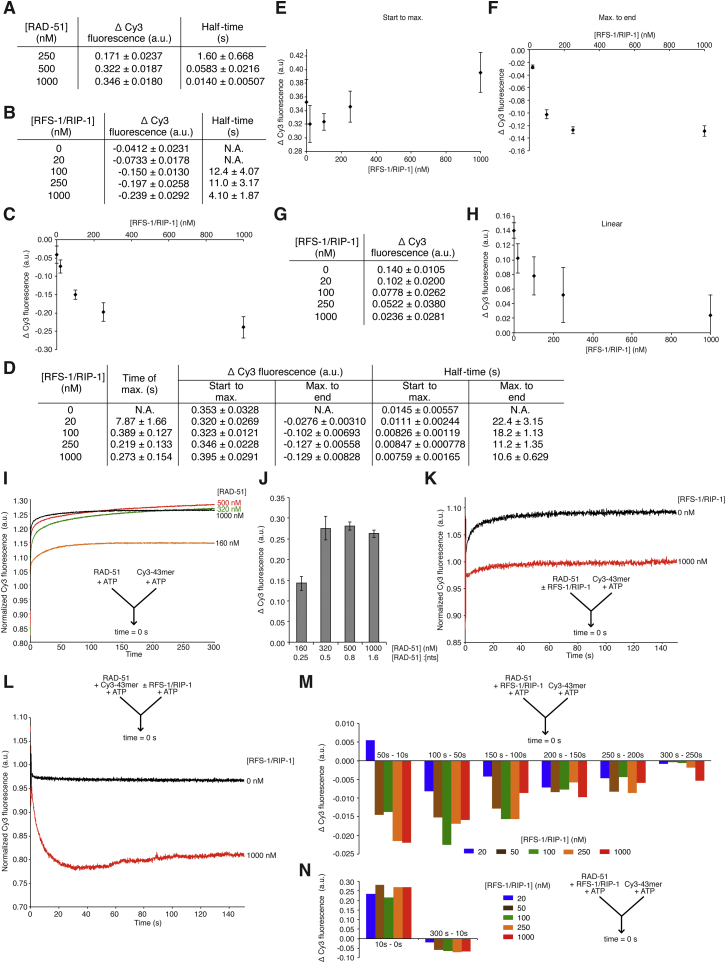
Analysis of Stopped-Flow Data for Remodeling of RAD-51-ssDNA Filaments by RFS-1/RIP-1, Related to Figure 4 (A–N) All fluorescence values for start, end and maximum fluorescence and corresponding time values were determined using ten-point moving averages calculated for all data sets (see the Supplemental Experimental Procedures for details). (A) Table of average difference in normalized Cy3 fluorescence (Δ Cy3 fluorescence) and average time to reach half of the fluorescence change (half-time) over the full time course at different RAD-51 concentrations presented in Figure 4A. Error bars indicate SD (n = 8-9). (B) Table of average Δ Cy3 fluorescence and half-time over the full time course at different RFS-1/RIP-1 concentrations presented in Figure 4C. Half-time values for 0 and 20 nM RFS-1/RIP-1 could not be determined since the initial noise in the dataset due to sample equilibration impairs reliable estimation. Error bars indicate SD (n = 6–9). (C) Graph of average Δ Cy3 fluorescence as a function of RFS-1/RIP-1 concentration plotted in linear scale for the data presented in (B). Error bars indicate SD (n = 6–9). (D) Table showing the average time the maximum in fluorescence is observed (Time of max.), and average Δ Cy3 fluorescence and half-times for both the first phase of increasing fluorescence (Start to max.) and the second phase of decreasing fluorescence (Max. to end) at different RFS-1/RIP-1 concentrations presented in Figure 4D. Errors indicate SD (n = 5–7). (E and F) Graphs of Δ Cy3 fluorescence from start to max. (E) and max. to end (F) as a function of RFS-1/RIP-1 concentration for the data presented in (D). Error bars indicate SD (n = 5–7). (G) Table of average Δ Cy3 fluorescence over the full time course at different RFS-1/RIP-1 concentrations presented in Figure 4E. Errors indicate SD (n = 6–9). (H) Graph of average Δ Cy3 fluorescence as a function of RFS-1/RIP-1 concentration plotted in linear scale for the data presented in (G). Error bars indicate SD (n = 6–9). (I) Analysis of average normalized Cy3-43-mer fluorescence plotted as a function of time over 300 s, for indicated concentrations of RAD-51 rapidly mixed with 15 nM Cy3-43-mer (both +ATP) at the 0 s time point in a stopped-flow instrument (indicated by arrow inset) (n = 3–4). (J) Bar chart showing average Δ Cy3 fluorescence over the full time course at different RAD-51 concentrations presented in (A). Errors indicate SD (n = 3–4). (K and L) Analysis of normalized Cy3-43-mer fluorescence plotted as a function of time over 150 s, demonstrating reactions attain equilibrium. The arrow (inset) indicates the components of the two syringes rapidly mixed at the 0 s time point in a stopped-flow instrument. (K) 1000 nM RAD-51 pre-incubated with indicated concentrations of RFS-1/RIP-1 for 10 min without ATP, then mixed with 15 nM Cy3-43-mer and ATP. (L) RAD-51-ssDNA filaments pre-formed with 1000 nM RAD-51 + 15 nM Cy3-43-mer for 10 min, then mixed with indicated concentrations of RFS-1/RIP-1 (both +ATP). (M) Graphs of Δ Cy3 fluorescence for indicated time intervals over 300 s showing reactions reach equilibrium for an experiment where 1000 nM RAD-51 was pre-incubated with indicated concentrations of RFS-1/RIP-1 for 10 min, then mixed with 15 nM Cy3-43-mer (both +ATP). (N) Graphs of Δ Cy3 fluorescence for time intervals corresponding to the filament formation phase (10 s – 0 s) and filament remodeling phase (300 s – 10 s) for the same experiment as in (M).

To examine the functional impact of RFS-1/RIP-1 on RAD-51-ssDNA filaments, we employed a stopped-flow system to monitor protein-ssDNA complex dynamics in real time by rapidly mixing different combinations of RAD-51, RFS-1/RIP-1, and a 5′-Cy3 fluorescently labeled (dT)_43_ oligonucleotide (Cy3-43-mer). Equal volumes of two solutions of interest were injected into a mixing chamber, and all concentrations quoted herein represent the value in the final reaction mixture. By monitoring changes in Cy3 fluorescence over time, we could quantitatively assess changes in the biophysical properties of the fluorophore as a proxy for changes in the nature of the protein-DNA association (Antony et al., 2009). Although fluorescence was stable in the absence of protein, rapid mixing of RAD-51 with the labeled DNA in the presence of ATP resulted in an increase in fluorescence as a function of time (Figure 4A). Both the size and the rate of change in fluorescence, represented as Δ Cy3 fluorescence and half-time, respectively, increased as a function of RAD-51 concentration (Figures S4A, S4I, and S4J) and serves as a readout for RAD-51-ssDNA filament formation. In contrast, addition of RFS-1/RIP-1 alone did not change fluorescence of Cy3-43-mer (Figure 4B).

### RFS-1/RIP-1 Changes the Biophysical Properties of Pre-formed RAD-51-ssDNA Filaments

Next, we examined the influence of RFS-1/RIP-1 on pre-formed RAD-51-ssDNA filaments using the stopped-flow system. Addition of RFS-1/RIP-1 caused a concentration-dependent decrease in fluorescence (Figures 4C, 4F, S4B, and S4C). The rate of fluorescence change also increased with RFS-1/RIP-1 concentration (half-time 12.40 ± 4.07 and 4.10 ± 1.87 s for 100 and 1,000 nM RFS-1/RIP-1, respectively; Figure S4B). This result demonstrates that RFS-1/RIP-1 is able to rapidly modulate pre-formed RAD-51-ssDNA filaments, arguing for a major activity of RFS-1/RIP-1 at a step downstream of RAD-51 loading onto ssDNA (Figures 3C and 3D).

We considered two interpretations for the reduction in fluorescence of RAD-51-ssDNA filaments induced by RFS-1/RIP-1: (1) filament disassembly or (2) filament remodeling. We do not favor the RAD-51-ssDNA filament disassembly model for the following reasons: (1) EMSA data show protein-DNA complex formation is increased, not reduced, in the presence of RFS-1/RIP-1 (Figures 3A, 3B, and S3A–S3E), (2) filaments do not change in length in EM after co-incubation with RFS-1/RIP-1 (Figures 3D–3G and S3F), (3) RFS-1/RIP-1 stimulates RAD-51 recombinase activity (Figures 2C and S2D), which is dependent on the RAD-51-ssDNA filament, and (4) RFS-1/RIP-1 promotes RAD-51 focus formation in vivo (Figure 1D). All these observations are in striking contrast to Srs2, which disassembles Rad51-ssDNA filaments, inhibits Rad51 recombinase activity, and antagonizes Rad51 focus formation in yeast (Burgess et al., 2009; Krejci et al., 2003). We therefore favor the second model, in which the reduction in fluorescence is attributed to a change in the biophysical properties (hereafter referred to as “remodeling”) of the RAD-51-ssDNA filaments.

Given the dramatic differences in the rates of fluorescence change attributed to RAD-51 binding and filament remodeling (compare half-times in Figures S4A and S4B), we asked whether these distinct phenomena could be temporally separated by pre-incubating RAD-51 with different concentrations of RFS-1/RIP-1, followed by rapid mixing with Cy3-43-mer (Figure 4D). RAD-51 alone displayed a profile of fluorescence increase with similar kinetics to the corresponding experiment in Figure 4A (Figures S4A and S4D). Remarkably, in the presence of RFS-1/RIP-1, a biphasic profile of fluorescence as a function of time was observed, with fluorescence reaching a maximum before declining. We attribute the rapid initial increase in fluorescence, which is only weakly influenced by RFS-1/RIP-1, to RAD-51-ssDNA filament formation (Figures 4D, S4D, and S4E). In contrast, after this initial rapid phase, a clear RFS-1/RIP-1 concentration-dependent reduction in fluorescence intensity was observed (Figures S4D and S4F). The rate of the second phase was comparable to the effect of RFS-1/RIP-1 on pre-formed RAD-51-ssDNA filaments observed in Figure 4C (Figures S4B and S4D), suggesting this slower phase represents the same slower filament-remodeling phase. As a variant of this experiment, we omitted ATP from the syringe containing the proteins (Figure 4E) and observed a much slower increase in fluorescence (compare Figures 4A and 4E), suggesting that ATP binding to RAD-51 primes it for rapid filament assembly. Notably, however, inclusion of RFS-1/RIP-1 led to a concentration-dependent reduction of fluorescence increase (Figures 4E, 4G, S4G, and S4H).

Analysis of longer time courses revealed that the fluorescence of the remodeled filaments reached equilibrium in all experiments (Figures S4K–S4N). For the experiment in which filament formation and remodeling were temporally segregated, at all RFS-1/RIP-1 concentrations ≥100 nM (Figures S4D, S4F, S4M, and S4N), a similar end point of the remodeling phase was attained that was intermediate to naked DNA and unremodeled filaments, demonstrating sub-stoichiometric quantities of RFS-1/RIP-1 are sufficient to drive RAD-51 filament remodeling. We also verified that RFS-1/RIP-1 stimulates D loop formation at the same RAD-51-to-ssDNA ratio as was used in the stopped-flow experiments (Figure S2E), confirming the relevance of these observations to the mechanism of RAD-51 recombinase stimulation by RFS-1/RIP-1.

### RFS-1/RIP-1 Stabilizes RAD-51-ssDNA Filaments

**Figure 5:**
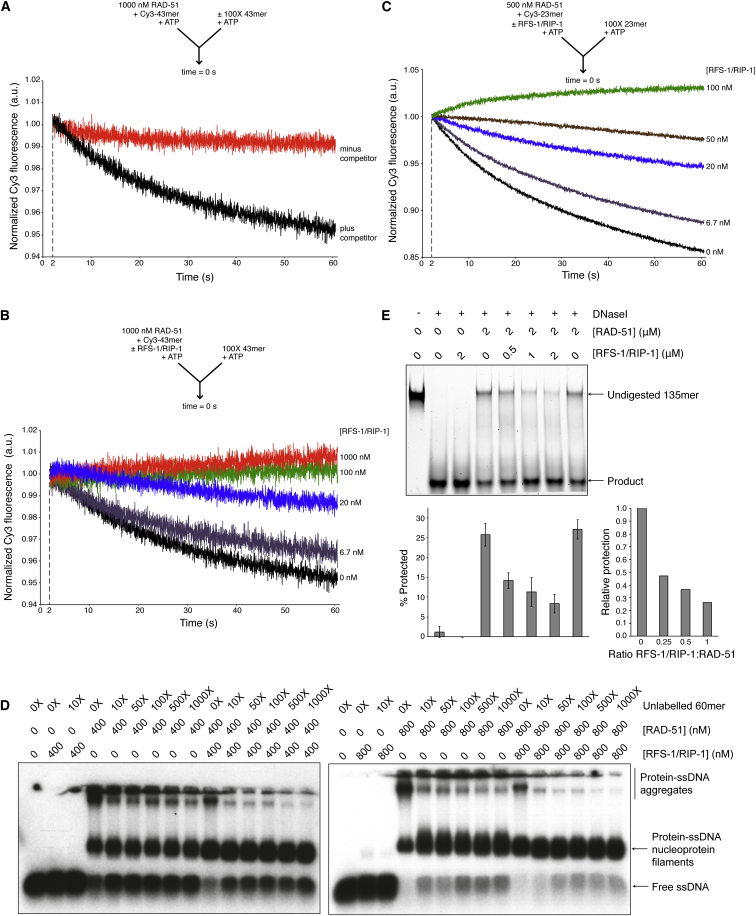
RFS-1/RIP-1 Stabilizes RAD-51-ssDNA Filaments in a Nuclease-Sensitive Conformation (A–E) Analysis of average normalized Cy3-43-mer fluorescence (see the Experimental Procedures for details) plotted as a function of time. The arrow (inset) indicates the components of the two syringes rapidly mixed at the 0 s time point in a stopped-flow instrument in the presence of ATP. (A) RAD-51-ssDNA filaments pre-formed with 1 μM RAD-51 + 15 nM Cy3-43-mer for 10 min and then mixed with (black) or without (red) 100-fold excess unlabeled 43-mer (n = 4–6). (B) RAD-51-ssDNA filaments pre-formed with 1 μM RAD-51 + 15 nM Cy3-43-mer and indicated concentrations of RFS-1/RIP-1 for 10 min and then mixed with 100-fold excess unlabeled 43-mer (n = 4–6). (C) RAD-51-ssDNA filaments pre-formed with 500 nM RAD-51 + 15 nM Cy3-23-mer and indicated concentrations of RFS-1/RIP-1 for 10 min and then mixed with 100-fold excess unlabeled 23-mer (n = 5–8). (D) Proteins were pre-incubated before addition of radiolabeled 60-mer ssDNA for 10 min and then challenged with the indicated molar excess of unlabeled 60-mer for a further 10 min. Protein-DNA complexes were crosslinked and resolved in agarose gels. (E) DNaseI protection assay on protein-DNA complexes formed by RAD-51 and RFS-1/RIP-1 on ssDNA with ATP. Error bars indicate SD (n = 4). The chart indicates the extent of protection by comparison with EMSA data in Figure S3B relative to RAD-51 alone. See also Figure S5.

**Figure S5:**
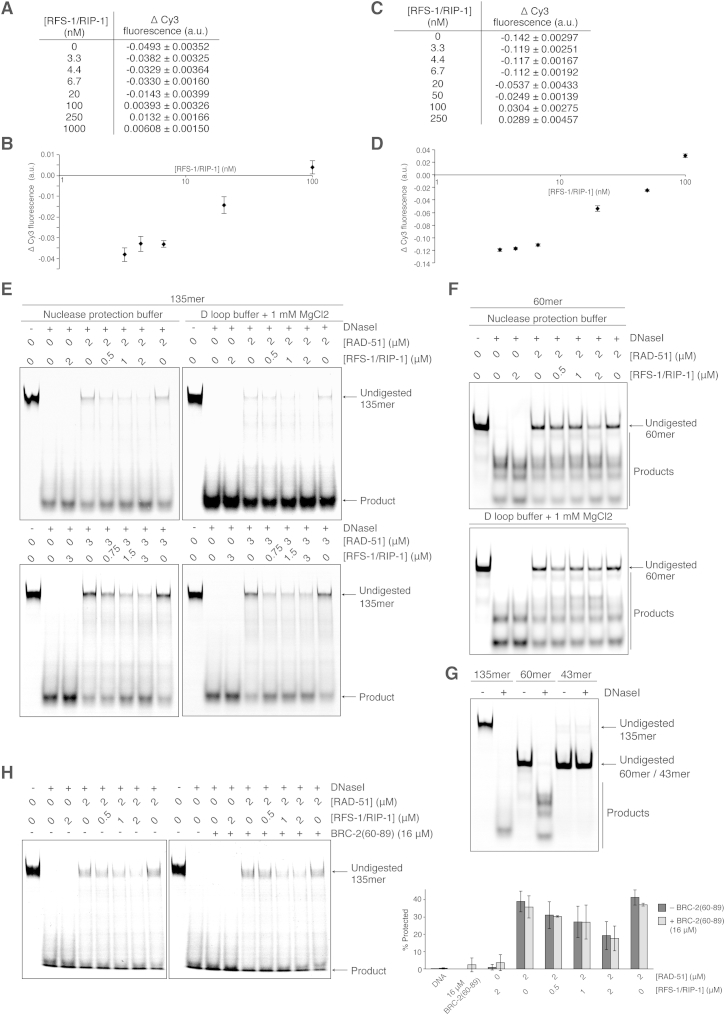
Analysis of Stopped-Flow Data for Stabilization of RAD-51-ssDNA Filaments by RFS-1/RIP-1 in a Nuclease-Sensitive Conformation, Related to Figure 5 (A) Table of average Δ Cy3 fluorescence at different RFS-1/RIP-1 concentrations for the data presented in Figure 5B (see the Supplemental Experimental Procedures for calculation details) using Cy3-43-mer DNA. Errors indicate SD (n = 4–6). (B) Graph of average Δ Cy3 fluorescence as a function of RFS-1/RIP-1 concentration plotted in log_10_ scale for the data presented in (A). Error bars indicate SD (n = 4–6). (C) Table of average Δ Cy3 fluorescence at different RFS-1/RIP-1 concentrations for the data presented in Figure 5C (see the Supplemental Experimental Procedures for calculation details) using Cy3-23-mer DNA. Errors indicate SD (n = 5–8). (D) Graph of average Δ Cy3 fluorescence as a function of RFS-1/RIP-1 concentration plotted in log_10_ scale for the data presented in (C). Error bars indicate SD (n = 5–8). (E and F) DNaseI protection assays on protein-DNA complexes formed by RAD-51 and RFS-1/RIP-1 on 135-mer (E) or 60-mer (F) ssDNA with ATP in either the same nuclease protection buffer as in Figure 5D or the D loop buffer (Figure 2C) supplemented with 1 mM MgCl_2_. (G) The 135-mer and 60-mer are substrates for DNaseI but the 43-mer used in stopped-flow is not efficiently digested by DNaseI. (H) DNaseI protection assays on protein-DNA complexes formed by RAD-51 and RFS-1/RIP-1 in the presence or absence of BRC-2(60-89) peptide on 135-mer ssDNA with ATP (left, gels; right, quantification). Error bars indicate SD (n = 3).

To determine whether RAD-51-ssDNA filament stability is influenced by RFS-1/RIP-1, we challenged RAD-51-ssDNA filaments pre-formed on Cy3-43-mer in the presence or absence of RFS-1/RIP-1 with a 100-fold excess of unlabeled, matched competitor 43-mer in the stopped-flow system. In the absence of RFS-1/RIP-1, fluorescence declined as a function of time, consistent with the ability of unlabeled competitor DNA to bind RAD-51 dissociated from the labeled DNA (Figure 5A). In contrast, addition of as little as 300-fold less RFS-1/RIP-1 (3.3 nM) reduced the extent of the change in fluorescence in a concentration-dependent manner (Figures 5B, S5A, and S5B), suggesting that RFS-1/RIP-1 stabilizes RAD-51 binding to ssDNA at sub-stoichiometric ratios. We verified this observation using half the amount of RAD-51 pre-bound to an oligonucleotide of approximately half the length (23-mer) (Figures 5C, S5C, and S5D). We also monitored filament stability directly by EMSA upon titration of unlabeled scavenger DNA. The levels of labeled free DNA liberated by RAD-51 turnover from the filament were reduced in the presence of RFS-1/RIP-1 (Figure 5D), verifying that the remodeled filaments are more stable. Interestingly, filaments formed in the presence of an excess of RAD-51 tended to aggregate and could not be resolved in agarose gels after crosslinking. This aggregation was reduced by RFS-1/RIP-1, while retaining a large population of resolved filaments (Figure 5D). Together, these data argue that RFS-1/RIP-1 remodels RAD-51-ssDNA filaments to a form in which RAD-51 is more stably associated with ssDNA and the filaments are less prone to aggregation after crosslinking, suggesting filament remodeling may reflect a conformational change in the pre-synaptic complex.

### RFS-1/RIP-1 Sensitizes ssDNA within RAD-51 Filaments to DNaseI Digestion

To directly probe for potential structural changes associated with remodeling of RAD-51-ssDNA filaments by RFS-1/RIP-1, we attempted to perform three-dimensional reconstructions from electron micrographs (Figure S3F). Averaged power spectra revealed that the pitch of the filament helix (∼90 Å) was unaltered with and without RFS-1/RIP-1 (Figure S3F). However, under the conditions used, the filaments were highly heterogeneous due to binding of RFS-1/RIP-1, precluding the generation of high-resolution reconstructions of filament architecture.

We reasoned that a change in the structural properties of the filaments could alter the accessibility of the ssDNA to degradation by nucleases (Zaitsev and Kowalczykowski, 1999). Using a nuclease protection assay to monitor the sensitivity of pre-formed protein-DNA complexes to DNaseI, we observed that the addition of sub-stoichiometric quantities of RFS-1/RIP-1 to RAD-51 caused a dramatic and unexpected increase in the DNaseI sensitivity of the protein-DNA complexes (Figure 5E). We verified these observations under similar buffer conditions to D loop formation and on the 60-mer oligonucleotide used in EMSA (Figures S5E–S5G), demonstrating that the effect is robust, independent of oligonucleotide properties, and relevant to the stimulation of RAD-51 recombinase activity.

Since RAD-51-ssDNA filaments are more stable in the presence of RFS-1/RIP-1 (Figure 5B–5D), de-protection is unlikely due to increased treadmilling by RAD-51 on ssDNA. To verify this, we performed the nuclease protection assay under conditions in which RAD-51 turnover from ssDNA is impaired. Since RAD-51-ssDNA filament disassembly is dependent on ATP hydrolysis by RAD-51 within the filament, we performed assays in the presence of a peptide of *C. elegans* BRC-2 that stabilizes RAD-51-ssDNA filaments by inhibiting RAD-51 ATP hydrolysis (Figure S5H) (Petalcorin et al., 2007). RFS-1/RIP-1 still conferred DNaseI sensitization, confirming this assay reflects a change to a more “open” filament conformation, rather than filament disassembly.

### Walker Box Mutations in RFS-1 Compromise Mediator Activity and Filament Remodeling

**Figure 6:**
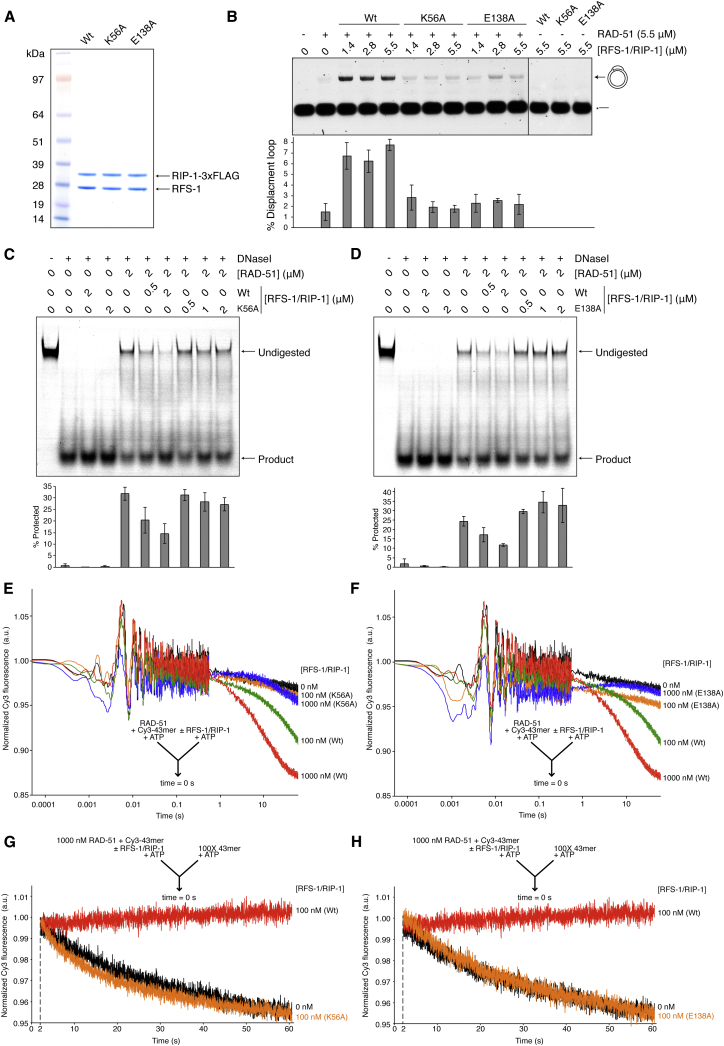
Walker Box Mutations in RFS-1 Prevent Filament Remodeling and Stimulation of Strand Exchange (A) Purification of recombinant RFS-1(K56A)/RIP-1 and RFS-1(E138A)/RIP-1 from yeast cells by FLAG immunoprecipitation. (B) RFS-1/RIP-1 mutants have impaired stimulation of D loop formation by RAD-51. Error bars indicate SD (n = 3). (C and D) RFS-1(K56A)/RIP-1 (C) and RFS-1(E138A)/RIP-1 (D) mutants do not remodel RAD-51-ssDNA filaments to a DNaseI-sensitive conformation. Error bars indicate SD (n = 3). (E and F) RFS-1(K56A)/RIP-1 (E) and RFS-1(E138A)/RIP-1 (F) mutants do not reduce the fluorescence of RAD-51-ssDNA filaments on Cy3-43-mer ssDNA in the same stopped-flow experimental setup as in Figure 4C (n = 6–8). For clarity, traces for wild-type (Wt) and RAD-51 alone are duplicated in (E) and (F). (G and H) RFS-1(K56A)/RIP-1 (G) and RFS-1(E138A)/RIP-1 (H) mutants do not remodel RAD-51-ssDNA filaments to a stable conformation in the same stopped-flow experimental setup as in Figure 5B (n = 5–8). For clarity, traces for Wt and RAD-51 alone are duplicated in (G) and (H). See also Figure S6.

**Figure S6:**
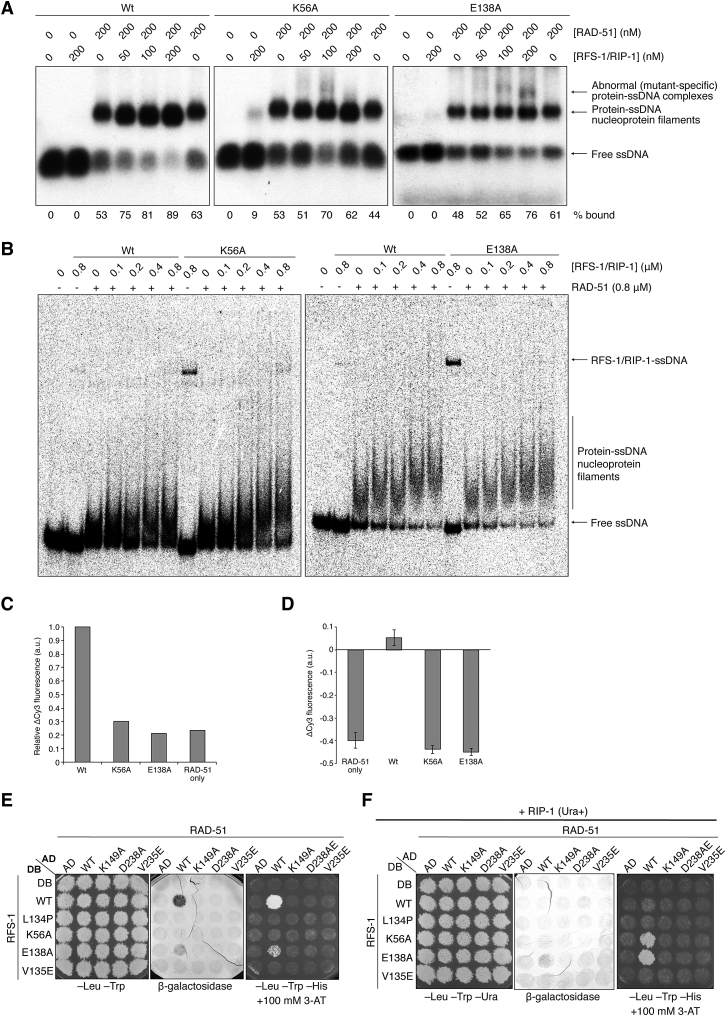
Walker Box Mutations in RFS-1 Are Permissive for Filament Binding but Prevent Remodeling, Related to Figure 6 (A) Protein-DNA complexes formed by RAD-51 and RFS-1/RIP-1 (Wt, K56A or E138A) on ssDNA with ATP. Proteins were pre-incubated before addition of ssDNA, then protein-DNA complexes crosslinked and resolved in agarose gels. (B) Protein-DNA complexes formed by RAD-51 and RFS-1/RIP-1 (Wt, K56A or E138A) on ssDNA with ATP. Proteins were pre-incubated before addition of ssDNA and resolved by native page. (C) Analysis of average Δ Cy3 fluorescence for K56A and E138A mutant complexes normalized to Wt for the data presented in Figures 6E and 6F (see the Supplemental Experimental Procedures for calculation details). Error bars indicate SD (n = 6–8). (D) Analysis of average Δ Cy3 fluorescence for K56A and E138A mutant complexes for the data presented in Figures 6G and 6H (see the Supplemental Experimental Procedures for calculation details). Error bars indicate SD (n = 5–8). (E) DB-RFS-1 and AD-RAD-51 interact via ATPase motifs in yeast two-hybrid, indicated by positive β-galactosidase expression and survival on media containing 3-aminotriazole (3-AT) in the absence of histidine. Growth on media lacking leucine and tryptophan is a positive control for plasmid transfection. (F) RIP-1 was co-expressed in the same strains as in (E) on a Ura-selectable plasmid and modulates the RFS-1/RAD-51 interaction network. Growth on media lacking leucine, tryptophan and uracil is a positive control for plasmid transfection.

The Walker motifs of Rad51 paralogs are important for resistance to DNA-damaging agents in vivo (French et al., 2003; Gruver et al., 2005; Wiese et al., 2006; Yamada et al., 2004), but the biochemical function of these motifs is unclear. To examine the functional importance of the Walker motifs in RFS-1 for the activity of RFS-1/RIP-1, we purified single-point mutants in conserved residues in either the Walker A (K56A) or the Walker B (E138A) box of RFS-1 (Figure 6A). Both mutants were defective for stimulation of D loop formation by RAD-51 (Figure 6B). At the same time, neither mutant was able to remodel the RAD-51-ssDNA filaments to a nuclease-sensitive conformation (Figures 6C and 6D) nor reduce the fluorescence of pre-formed RAD-51-ssDNA filaments on Cy3-labeled DNA in stopped-flow experiments (Figures 6E, 6F, and S6C). Furthermore, the filaments formed in the presence of these mutants were not stabilized against scavenger DNA (Figure 6G, 6H, and S6D), in contrast to the wild-type complex.

Although both mutant complexes bound to RAD-51-ssDNA filaments similarly to wild-type RFS-1/RIP-1 in EMSA (Figures S6A and S6B), they exhibited increased ssDNA affinity (Figure S6B) and formed abnormal protein-ssDNA complexes with RAD-51 after crosslinking (Figure S6A). We also assessed the effect of mutating these residues on the RFS-1/RIP-1/RAD-51 interaction network in yeast two-hybrid and discovered that the RFS-1/RAD-51 interaction was impaired (Figure S6E). Co-expression of RIP-1 in the RFS-1/RAD-51 yeast two-hybrid strains impaired the RFS-1/RAD-51 interaction, suggesting RAD-51 and RIP-1 compete for a common binding surface on RAD-51 in the absence of DNA, but this inhibition was defective in the context of the RFS-1 Walker box mutations (Figure S6F). Together, these observations suggest K56A and E138A mutant complexes interact abnormally with ssDNA, RAD-51, and RAD-51-ssDNA filaments. Crucially, these findings reveal that filament remodeling is intrinsic to the RFS-1/RIP-1 complex, required for its mediator activity, and dependent on the intact Walker boxes of RFS-1.

### RFS-1/RIP-1 Converts RAD-51-ssDNA Filaments to a More Flexible Conformation

**Figure 7:**
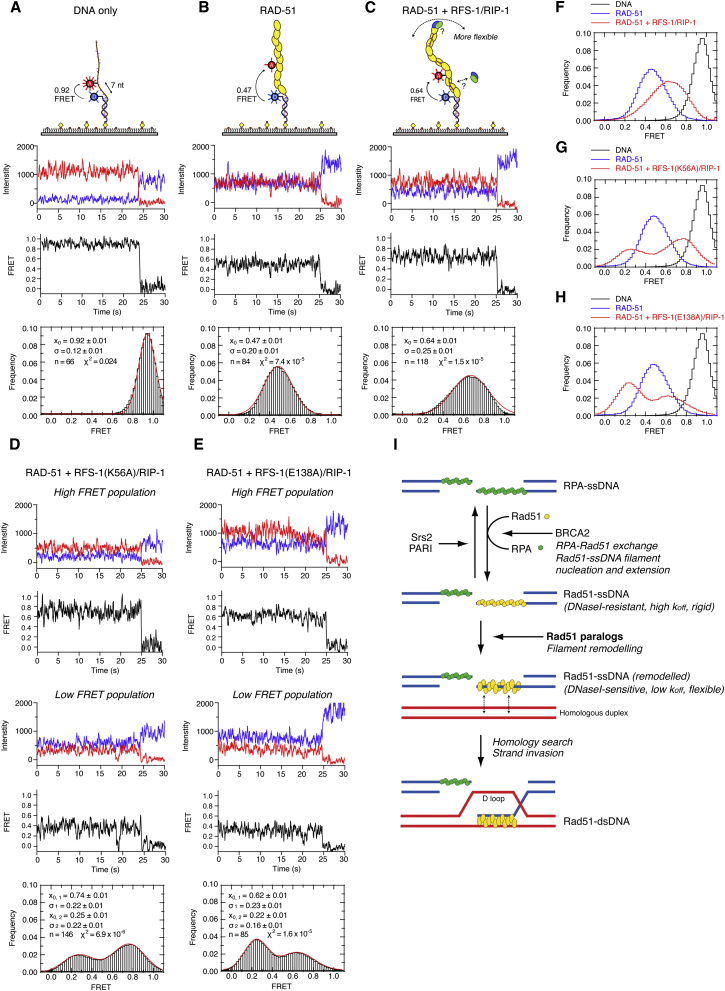
Remodeled RAD-51-ssDNA Filaments Are More Flexible (A–E) smFRET analysis of (A) DNA alone, (B) DNA + RAD-51, (C) DNA + RAD-51 + RFS-1/RIP-1 (Wt), (D) DNA + RAD-51 + RFS-1(K56A)/RIP-1, and (E) DNA + RAD-51 + RFS-1(E138A)/RIP-1. Top to bottom: cartoon schematic of the smFRET experiment indicating FRET between Cy3 and Cy5 (7-nt separation) attached to biotinylated DNA constructs immobilized on a streptavidin-coated biotin-PEG surface; donor (blue) and acceptor (red) intensity trajectories are anti-correlated until single-step photobleaching of the acceptor; FRET trajectories (black) between Cy3 and Cy5 exhibit a sharp drop to zero FRET when the acceptor photobleaches; histogram of FRET values collected from all molecules with a Gaussian fit are shown in red, where x_0_ is the mean FRET, σ is the distribution width, and χ^2^ quantifies the quality of the fit. (F–H) Superimposed histograms from (A)–(E) showing the differences between the populations. Differences in the mean FRET are statistically significant (Student’s t test; p < 0.0005). (I) Model for the proposed function of Rad51 paralogs in Rad51 filament remodeling within the HR mechanism. See also Figure S7.

**Figure S7:**
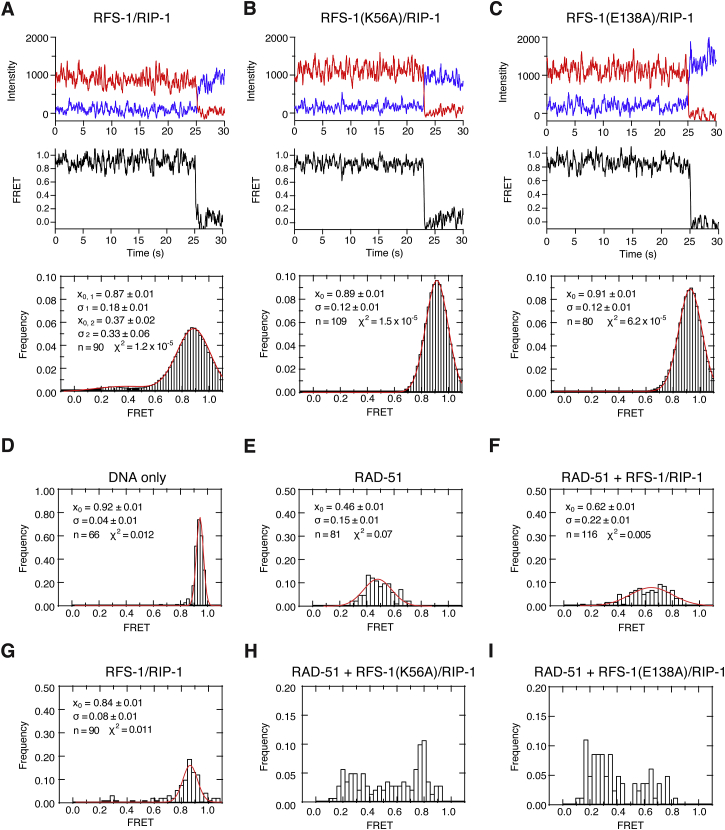
Remodeled RAD-51-ssDNA Filaments Are More Flexible, Related to Figure 7 (A–C) smFRET analysis of (A) DNA + RFS-1/RIP-1 (Wt), (B) DNA + RFS-1(K56A)/RIP-1, (C) DNA + RFS-1(E138A)/RIP-1. Top to bottom: cartoon schematic of the smFRET experiment indicating FRET between Cy3 and Cy5 (7 nt separation) attached to biotinylated DNA constructs immobilized on a streptavidin-coated biotin-PEG surface; donor (blue) and acceptor (red) intensity trajectories are anti-correlated until single-step photobleaching of the acceptor; FRET trajectories (black) between Cy3 and Cy5 exhibit a sharp drop to zero FRET when the acceptor photobleaches; histogram of mean FRET values for individual molecules with a Gaussian fit shown in red, where x_0_ is the mean FRET, σ is the distribution width, and χ^2^ quantifies the quality of the fit. (D–I) Histograms of binned mean FRET values determined from each trajectory individually with Gaussian fits superimposed (red curves) (D) DNA only; (E) DNA + RAD-51; (F) DNA + RAD-51 + RFS-1/RIP-1 (Wt); (G) DNA + RFS-1/RIP-1 (Wt); (H) DNA + RAD-51 + RFS-1(K56A)/RIP-1; (I) DNA + RAD-51 + RFS-1(E138A)/RIP-1. Differences in the mean are statistically significant (p < 0.0005).

To probe the nature of the conformational change induced in the pre-synaptic complex by RFS-1/RIP-1, we performed single-molecule FRET (smFRET) experiments. Filament properties were monitored by measuring FRET efficiency between a Cy3 donor and a Cy5 acceptor fluorophore, separated by seven nucleotides in surface-immobilized ssDNA constructs (Figure 7A). RAD-51 binding to ssDNA results in a dramatic decrease from 0.92 ± 0.01 (naked DNA; Figure 7A) to 0.47 ± 0.01 (DNA + RAD-51; Figure 7B) in mean FRET (x_0_), due to both ssDNA stretching within the filament and a reduction in molecular flexibility relative to naked ssDNA. Co-incubation of RAD-51 with RFS-1/RIP-1 induces a striking increase to 0.64 ± 0.01 (Figures 7C and 7F) in mean FRET, relative to RAD-51 alone, as well as a broadening of the FRET distribution, indicated by the distribution width (σ), from σ = 0.20 ± 0.01 (DNA + RAD-51; Figure 7B) to σ = 0.25 ± 0.01 (DNA + RAD-51 + RFS-1/RIP-1; Figure 7C). In contrast, the majority of the DNA molecules bound by RFS-1/RIP-1 yield a mean FRET of 0.87 ± 0.01, similar to naked DNA (Figure S7A). These results were verified by binning the average FRET value of each trajectory independently (Figures S7D–S7G), instead of time binning each trajectory (Figures 7A–7C and S7A). Given that the filament helical pitch is equivalent in the presence and absence of RFS-1/RIP-1 (Figure S3F), this increase in FRET is unlikely due to filament compression. Instead, these results suggest that in the presence of RFS-1/RIP-1 the filaments adopt a substantially more flexible and less rigid conformation.

We also tested the effect of the K56A and E138A mutants of RFS-1 on filament flexibility by smFRET (Figures 7D, 7E, S7H, and S7I). Similar to wild-type RFS-1/RIP-1, neither mutant complex alone significantly altered FRET relative to naked DNA (Figures S3B and S3C). However, in the presence of RAD-51, we observed a bimodal FRET distribution of the molecules, which is not observed with wild-type RFS-1/RIP-1. The high FRET populations (mean FRET 0.74 ± 0.01 and 0.62 ± 0.01 for K56A and E138A, respectively) were similar to those observed for RAD-51 filaments co-incubated with wild-type RFS-1/RIP-1 (Figures 7F–7H), suggesting some filaments become flexible in the presence of the mutant RFS-1/RIP-1 complexes. In contrast, the low FRET populations (mean FRET 0.25 ± 0.01 and 0.22 ± 0.01 for K56A and E138A, respectively) exhibit lower mean FRET values than that observed for RAD-51 alone (Figures 7G and 7H), which may represent a more rigid intermediate state in the remodeling process, not observed in the presence of the RFS-1/RIP-1 mutant complexes alone (Figures S7B and S7C). These results establish that RFS-1/RIP-1 K56A and E138A mutant complexes are compromised for inducing or maintaining the filament in a flexible high FRET state, consistent with their defects in filament remodeling and mediator activity in ensemble experiments (Figure 6).

## Discussion

### Rad51 Paralogs Remodel Rad51 Filaments to a Conformation More Proficient to Strand Exchange

In this study, we identify a biochemically tractable Rad51 paralog complex, RFS-1/RIP-1, which binds to and strongly stimulates the recombinase activity of RAD-51. Our biochemical and biophysical analysis reveals that the stimulatory activity of this Rad51 paralog complex on HR is due to its ability to induce a conformational change in the pre-synaptic filament. In contrast to BRCA2, RFS-1/RIP-1 does not primarily act to nucleate or extend RAD-51-ssDNA filaments. Instead, RFS-1/RIP-1 structurally remodels the pre-synaptic filament to a more “open,” flexible, and stable conformation. Importantly, RAD-51-ssDNA filament remodeling is an intrinsic property of RFS-1/RIP-1 that is dependent on the Walker boxes of RFS-1. Since the Walker box mutants in RFS-1 are also deficient for stimulation of RAD-51 strand exchange activity, we propose that filament remodeling is a crucial molecular switch through which the Rad51 paralogs stimulate HR. Thus, our study provides mechanistic insights into the functional importance of the Walker boxes in Rad51 paralogs in resisting DNA damage in cells.

### Biological Importance of Rad51 Filament Remodeling to HR

*rfs-1* and *rip-1* mutant animals are defective for RAD-51 focus formation at stalled replication forks, and we propose this likely reflects a failure to remodel and stabilize RAD-51-ssDNA filaments. Although RAD-51 would be loaded onto chromatin normally in the absence of *rfs-1* or *rip-1*, its turnover would be more rapid, preventing detection of RAD-51 foci. Since Rad51 focus formation after damage is also defective in Rad51 paralog mutants from other eukaryotic organisms (Gasior et al., 1998), the filament remodeling function of RFS-1/RIP-1 to a stable conformation is likely to be conserved. This result is also consistent with the suppression of the DNA damage sensitivity and HR defects of yeast *rad55Δ* and *rad57Δ* mutants by a mutant form of Rad51 (I345T) that binds DNA more stably (Fortin and Symington, 2002).

A requirement for Rad51 paralogs in preventing filament disruption by Srs2 at DSBs (Liu et al., 2011a) is likely dispensable in nematodes as they lack a Srs2 ortholog. Although *rfs-1* and *rip-1* animals are sensitive to both IR and nitrogen mustard, but only defective in RAD-51 focus formation in response to the latter, this likely reflects the additional function for RFS-1/RIP-1 in late stages of meiotic DSB repair (Ward et al., 2010). Furthermore, the sensitivity of *rfs-1* animals to IR is relatively weak compared to deficiency in the core HR component BRC-1, whereas sensitivity of *rfs-1* and *brc-1* animals to crosslinking agents is virtually indistinguishable, in support of the greater specificity of RFS-1/RIP-1 toward stalled replication fork substrates (Ward et al., 2007).

### Implications of Rad51 Filament Remodeling for the Homology Search

Structural remodeling of RAD-51-ssDNA filaments by RFS-1/RIP-1 to an “open,” flexible, and stabilized conformation also has implications for the efficiency of the homology search. The structural transitions undergone by Rad51 filaments during homology sampling, template unwinding, strand exchange, and conversion of Rad51-ssDNA to Rad51-dsDNA filaments are extremely challenging to study due to their dynamic nature. Atomic resolution models of the RecA-ssDNA filament show that each nucleotide triplet associated with each RecA protomer adopts a local B-DNA conformation, but the DNA is stretched and underwound from one triplet to the next, resulting in a global underwound DNA conformation. This stretching may facilitate disruption of base pairing and stacking in the template duplex upon binding by the filament (Chen et al., 2008; Danilowicz et al., 2014). The DNaseI sensitivity of the remodeled filament indicates that the ssDNA is more accessible, and it is therefore possible that the remodeling induced by Rad51 paralogs exposes the ssDNA to facilitate homology probing after filament binding to and disruption of the donor duplex. A recent study demonstrated that Rad51 only stably captures template dsDNA harboring at least eight nucleotides of homology, reducing search complexity, and argued that physical discontinuities or gaps within the pre-synaptic complex could further limit search complexity by segregating the Rad51-ssDNA filament into non-overlapping functional search units (Qi et al., 2015). The binding of RFS-1/RIP-1 to or within the pre-synaptic filament could define such search unit boundaries. Alternatively, it is possible that the DNaseI sensitivity of the remodeled filament arises due to flexing of the protomers transiently, exposing naked ssDNA, or due to limited turnover of individual protomers within the filament (without complete filament disassembly), which could also introduce pre-synaptic complex discontinuities to facilitate homology searching.

It has also been known for many years that homology searching by RecA proceeds primarily by a processive 3D search process, facilitated by transient contacts with heterologous dsDNA to enhance the probability of locating homologous sequences (Forget and Kowalczykowski, 2012; Gonda and Radding, 1983, 1986; Honigberg et al., 1986; Tsang et al., 1985). The increased flexibility of the remodeled filament induced by RFS-1/RIP-1 in 3D space could aid such a 3D homology search mechanism. The increased stability of the remodeled Rad51 filament may also increase the lifetime of the homology search and provide more opportunities to locate the correct dsDNA template. Hence, all three altered properties of the remodeled filament are predicted to facilitate homology search during HR, and remodeling could therefore have a very important function in vivo in locating the correct dsDNA template.

### Conclusions

Our observations, together with previously reported findings, suggest that Rad51 paralogs perform two distinct functions to promote HR: they protect Rad51-ssDNA filaments against disruption by antirecombinases (Liu et al., 2011a) and directly stimulate the intrinsic recombinase activity of Rad51 by remodeling pre-synaptic filaments to an active, “open,” flexible, and stable conformation primed for homology search and strand invasion. Importantly, the mechanism we have discovered for the Rad51 paralogs in stimulating HR is distinct from that proposed for other positive regulators of HR, which are epistatic to Rad51 paralogs, including BRCA2 and Rad54 (Chun et al., 2013; Jensen et al., 2010, 2013; Liu et al., 2010; Solinger et al., 2002; Thorslund et al., 2010). We therefore propose a model (Figure 7I) for HR, in which Rad51-ssDNA filaments are first nucleated by BRCA2, displacing RPA from ssDNA. Rad51 paralogs subsequently switch the filament to a more “open” and flexible structure, which is also less prone to disassembly. The altered properties of this remodeled pre-synaptic filament likely facilitate homology probing of the template and strand invasion to stimulate HR.

## Experimental Procedures

RFS-1/RIP-1 complex was co-expressed in yeast cells and purified by FLAG immunoprecipitation. RAD-51 was purified using the pET-SUMO system and the SUMO tag cleaved with Ulp1 SUMO protease to yield native protein followed by MonoQ ion-exchange chromatography. For EMSA, protein-DNA complexes were assembled on ^32^P-labeled 60-mer ssDNA in the presence of ATP (10 min) and then resolved by native PAGE or crosslinked with 0.25% glutaraldehyde and resolved in agarose gels. In immuno-shift experiments, protein-DNA complexes assembled on fluorescently labeled 60-mer ssDNA were incubated with anti-FLAG antibody (Sigma F3165; 5 min) and resolved in agarose gels. Immuno-gold EM was performed by incubating RAD-51, RFS-1/RIP-1, and linearized PhiX ssDNA (10 min) and then incubating with anti-FLAG antibody conjugated to 20-nm gold particles (30 min), staining with uranyl acetate (2 min), and imaging. For nuclease protection assays, protein-DNA complexes were assembled on fluorescently labeled 135-mer ssDNA (10 min) before challenging with DNaseI (20 min), deproteinizing, and resolving DNA products by PAGE. D loop formation was performed by pre-incubating proteins and fluorescently labeled 90-mer ssDNA (15 min) before addition of pBluescript plasmid DNA (15 min), after which time reactions were deproteinized and resolved in agarose gels. Stopped-flow experiments were performed by rapidly mixing equal volumes of the indicated components and monitoring Cy3 fluorescence for 1 min using the following measurement protocol: (1) every 0.00005 s from 0–0.05 s, (2) every 0.0005 s from 0.05–0.56 s, and (3) every 0.02 s from 0.56–60.54 s. Raw data sets were normalized as follows: for binding and remodeling experiments (Figures 4, 6E, and 6F), data sets were normalized to the same starting value for Cy3 fluorescence, and for competition experiments (Figures 5A–5C, 6G, and 6H), data sets were normalized to the same value for Cy3 fluorescence at the 2.01998 s time point and truncated before this. Yeast two-hybrid and nematode genetic analysis were performed as previously described (Boulton et al., 2002; Ward et al., 2007, 2010). Full materials and methods, including details of stopped-flow data analysis and smFRET, are available in the Supplemental Experimental Procedures.

## Supplemental Information

Document S1. Supplemental Experimental Procedures

Document S2. Article plus Supplemental Information
