# Supplementary material for: Rad51 Paralogs Remodel Pre-synaptic Rad51 Filaments to Stimulate Homologous Recombination
Source: Cell. 2015 Jul 16;162(2):271–86. doi: 10.1016/j.cell.2015.06.015 (PMC4518479; doi:10.1016/j.cell.2015.06.015)
Supplement: Document S1. Supplemental Experimental Procedures [file mmc1.pdf]

Cell

Supplemental Information

Rad51 Paralogs Remodel Pre-synaptic Rad51 Filaments to Stimulate Homologous Recombination

Martin R.G. Taylor, Mário Špírek, Kathy R. Chaurasiya, Jordan D. Ward, Raffaella Carzaniga, Xiong Yu, Edward H. Egelman, Lucy M. Collinson, David Rueda, Lumir Krejčí, and Simon J. Boulton

Supplemental Experimental Procedures

Cloning of protein expression constructs

Codon optimized cDNA encoding native RFS-1 and C-terminally 3xFLAG-tagged RIP-1 for expression in budding yeast were obtained from Geneart (Life Technologies). The sequence of the tag immediately downstream of RIP-1 is: LGGGGSGGGGDYKDDDDKDYKDDDDKDYKDDDDK. These constructs were cloned into a modified form of the pRS306 vector containing the bidirectional Gal1-10 promoter cloned between the AscI and SgrAI restriction sites, as described previously (Frigola et al., 2013). RFS-1 was cloned between the SgrAI and NotI restriction sites and RIP-1 between the AscI and SmaI restriction sites, and the construct integrated into yeast strain yJF1 (*W303-1a pep4::KanMx4 bar1::Hph-NT1 ade2-1 ura3-1 his3-11 trp1-1 leu2-3*) (gift of John Diffley and Jordi Frigola) after linearization at the NcoI unique restriction site in the URA3 cassette. Site directed mutagenesis to generate mutant expression constructs was performed using the QuikChange Site-Directed Mutagenesis Kit (Stratagene). Protein expression was confirmed in small scale cultures by growing cells in YP + 2% raffinose at 30°C until OD_{600} of 0.7-0.8, before induction for 5 h with 2% galactose. Samples of 5 x 10^8 cells were collected before and after induction, lysed with alkaline and proteins precipitated with trichloroacetic acid (TCA). The precipitate was harvested and resuspended in sample buffer and analysed by SDS-PAGE.

Codon optimized cDNA encoding native RAD-51 (short isoform) in *E. coli* was obtained from Geneart (Life Technologies) and cloned in the Champion pET-SUMO vector (Life Technologies) according to the manufacturer's instructions.

RFS-1/RIP-1 expression and purification

RFS-1/RIP-1 (Wt, K56A or E138A) were expressed in budding yeast cells by growing a 100 L culture in a 120 L BIOFLO 5000 fermenter (New Brunswick Scientific) in YP media + 2% raffinose at 30°C, pH 5.8, agitation 200 rpm and airflow 20 l/min until OD_{600} of 0.7-0.8, before induction for 5 h with 2% galactose. The cells were harvested and washed twice with Yeast Wash Buffer (25 mM HEPES-KOH (pH 7.5), 1 M sorbitol) then once with Buffer K (45 mM HEPES-KOH, 10% glycerol, 0.1 M potassium glutamate, 5 mM magnesium acetate) supplemented with 0.02% NP-40. The cells were then resuspended in 70 ml buffer K + 0.02% NP-40 supplemented with cOmplete, EDTA-free protease inhibitor cocktail tablets (Roche) (1 tablet per 25 ml buffer) and frozen dropwise in liquid nitrogen. Cells were lysed by crushing in a freezer mill (SPEX CertiPrep 6850) for 6 x 2 min cycles at rate 15 under liquid nitrogen and the frozen cell powder stored at –80°C. For purification, the cell

powder was thawed to room temperature in a water bath, diluted with Buffer K and the potassium glutamate concentration adjusted to 0.5 M in a total volume of 780 ml, and mixed well with a magnetic stirrer at 4°C until the lysate was homogenous. The lysate was cleared in an Optima LE-80K Ultracentrifuge (Beckman Coulter) using a Ti45 rotor at 40,000 rpm for 90 min at 4°C and the supernatant was applied to 10 ml bed volume of anti-FLAG M2 affinity gel (Sigma A2220), which had been pre-washed with 0.1 M glycine (pH 3.5), TBS and Buffer K according to the manufacturer's instructions. The protein was bound to the beads by rotating at 4°C overnight then applied to 5 x 25 ml batch purification columns. All subsequent steps were carried out at 4°C. The flow-through was discarded and the beads washed with 750 ml Buffer K (150 ml per column). The protein was eluted with 1 mg/ml 3xFLAG peptide (synthesised by Nicola O'Reilly, Peptide Chemistry Laboratory, The Francis Crick Institute) in Buffer K by passing 2 ml twice over each column then washing with 4 ml Buffer K per column (total eluate volume 30 ml), and dialyzed against 4 L Storage Buffer (20 mM Tris-acetate (pH 8.0), 100 mM potassium acetate, 10% glycerol, 1 mM EDTA, 0.5 mM DTT) overnight using 10 kDa MWCO SnakeSkin dialysis tubing (Thermo Scientific). The protein was then concentrated on a 30 kDa MWCO Amicon Ultra-15 Centrifugal Filter Unit pre-washed with water and Storage Buffer to a final volume of 1 ml, frozen in small aliquots in liquid nitrogen and stored at –80 °C. This method typically yielded 3-3.6 mg/ml recombinant RFS-1/RIP-1 (55-65 μM complex), devoid of detectable nuclease activity. Antibodies to detect RFS-1 and RIP-1 were raised in-house against peptides from each protein in rabbit or guinea pig.

RAD-51 expression and purification

For some EMSA experiments, RAD-51 was purified as described previously (Petalcorin et al., 2006). For all other experiments, RAD-51 was purified using the Champion pET-SUMO system (Life Technologies). The protein was expressed in BL21(DE3) One Shot *E. coli* by inoculating 6 L LB pre-warmed to 37 °C and supplemented with 50 μg/ml kanamycin to OD_{600} of ~0.01 from an overnight culture in 2TY grown at 30 °C. The culture was grown at 37 °C to OD_{600} of 0.6-0.8, before induction for 4 h with 1 mM IPTG at 30 °C. The cells were harvested at 4 °C and the pellets frozen on dry ice and stored at –80 °C. For purification, the pellets were thawed at 37 °C in a water bath and resuspended in 400 ml ice cold Lysis Buffer (50 mM potassium phosphate (pH 7.8), 1 M KCl, 10% glycerol) supplemented with cOmplete, EDTA-free protease inhibitor cocktail tablets (Roche) (1 tablet per 25 ml buffer), and mixed well with a magnetic stirrer at 4 °C until the mixture was homogenous. The cells were lysed by addition of Triton X-100 to 0.1% on the stirrer. The lysate was transferred to 4 x 100 ml flasks and each flask sonicated on ice 3 x 2 min with a large flat tip using a Branson Sonifier 450 (duty

cycle 80%, output control 8). The lysate was cleared in an Optima LE-80K Ultracentrifuge (Beckman Coulter) using a Ti45 rotor at 40,000 rpm for 60 min at 4 °C. Imidazole was added to the supernatant to a final concentration of 25 mM and applied to 12 ml bed volume of Ni-NTA agarose affinity gel (Qiagen 30210), which had been pre-washed with Binding Buffer (50 mM potassium phosphate (pH 7.8), 1 M KCl, 10% glycerol, 25 mM imidazole (pH 7.5)). The protein was bound to the beads by rotating at 4 °C for 2 h then applied to 6 x 25 ml batch purification columns. All subsequent steps were carried out at 4 °C. The flowthrough was discarded and the beads washed with 300 ml Binding Buffer and 300 ml Binding Buffer containing 50 mM imidazole (50 ml per column). The protein was eluted with Binding Buffer containing 200 mM imidazole by passing 8 ml twice over each column then washing with 4 ml Buffer K per column (total eluate volume 72 ml), and dialyzed against 4 L Dialysis Buffer (20 mM Tris-HCl (pH 8.0), 300 mM KCl, 10% glycerol) overnight using 10 kDa MWCO SnakeSkin dialysis tubing (Thermo Scientific). Some precipitate was observed the following day, but most protein remained in solution. The His-SUMO tag was cleaved to yield native RAD-51 by addition of 6 µl His-tagged Ulp1 SUMO protease (gift from Peter Cherepanov) for 45 min. The protein was centrifuged and the soluble fraction collected and bound to the same batch of NiNTA agarose affinity gel used for purification after regeneration according to the manufacturer's instructions to remove the SUMO protease and His-SUMO tag. The flowthrough containing native RAD-51 was collected and the resin washed with an additional 18 ml (3 ml per column) of Dialysis Buffer. These were pooled (total 90 ml) and mixed at 1:1 ratio with Dilution Buffer (20 mM Tris-HCl (pH 8.0), 10% glycerol, 2 mM EDTA, 1 mM DTT) (total 180 ml) to reduce salt concentration to 150 mM KCl. The protein was bound to a 1 ml Mono Q 5/50 GL column (GE Healthcare) at 0.5 ml / min using an Äkta Explorer HPLC system and washed with 30 ml R buffer (20 mM Tris-HCl (pH 8.0), 10% glycerol, 1 mM EDTA, 0.5 mM DTT) supplemented with 100 mM KCl then 20 ml R buffer supplemented with 150 mM KCl. The protein was eluted with a 30 ml gradient 150-640 mM KCl in R buffer and 0.5 ml fractions collected. The peak fractions were pooled and either dialysed against Storage Buffer for 1 h, frozen in small aliquots in liquid nitrogen and stored at –80 °C, or concentrated and frozen directly in the elution buffer. This method typically yielded 1.5-2.5 mg/ml recombinant RAD-51 (40-65 µM), which could be concentrated as high as 550 µM without precipitation.

Analytical size exclusion chromatography

30 μl of RFS-1/RIP-1 was loaded onto a 3 ml Superdex 75 5/150 GL column at 0.1 ml/min using an Ettan LC system and 0.1 ml fractions collected. Migration of molecular weight standards of 670, 158, 44, 17, 1.35 kDa (Biorad 151-1901) allowed estimation of a molecular weight for the complex.

RFS-1 expression in *E. coli*

N-terminal 6xHis, GST and MBP fusions of RFS-1 were cloned using the Gateway system under the control of the T7 promoter. Constructs were transformed into BL21(DE3) *E. coli* strains. Protein expression was performed by growing cells at 30°C until OD_{600} of 0.6, before induction for 4 h with 1 mM IPTG. Soluble extracts were prepared by harvesting cells, resuspending in 20 mM Tris-HCl (pH 7.4), 200 mM NaCl, 1 mM EDTA (pH 8.0), sonicating, then clearing the lysate at 4°C.

EMSA

Proteins were diluted from concentrated stocks into Storage Buffer, which was also used in no protein controls. For native polyacrylamide gels, proteins were mixed with a master mix (containing 100 nM (nucleotides) 5'-[^{32}P]-labelled 60mer oligonucleotide (ACGCTGCCGAATTCTACCAGTGCCTTGCTAGGACATCTTTGCCCACCTGCAGGTTCACCC), 20 mM Triethanolamine-HCl (pH 7.5), 8% glycerol, 1 mM DTT, 50 mM sodium acetate, and either 2 mM $MgCl_2$ and 2 mM of the indicated nucleotide, or 10 mM EDTA (pH 8.0)) according to the indicated scheme in 10 μl reaction volume at 25°C. Double-stranded DNA was prepared by annealing the above 60mer to its reverse complement. The reactions were transferred to ice and resolved on 8% native PAGE in 1X TBE using a Protean II xi cell system (Biorad) (200 V, 4 h 20 min, 4°C). For agarose gels, proteins were pre-incubated for 5 min then mixed with a master mix as above for 10 min, before crosslinking with 0.25% glutaraldehyde for 10 min, all at 25°C. In competition experiments, the same 60mer was added in unlabelled form in the indicated molar excess after protein-DNA complex formation for a further 10 min. Reactions were resolved on 1% agarose gels in 1X TBE (70 V, 2 h 20 min). Gels were dried and imaged by autoradiography or using a storage phosphor screen (Amersham Biosciences) and a Storm 840 molecular imager (Molecular Dynamics) and quantified using ImageQuant TL (Amersham Biosciences).

For immuno-shift experiments, proteins were incubated with 600 nM (nucleotides) of a 5'-fluorescein labelled version of the same 60mer for 10 min, before incubating protein-DNA complexes with anti-FLAG antibody (Sigma F3165) for 5 min and resolving in native agarose gels.

Nuclease protection assays

Proteins were diluted from concentrated stocks into T Buffer (25 mM Tris-HCl (pH 7.5), 10% glycerol, 0.5 mM EDTA (pH 8.0), 50 mM KCl), which was also used in no protein controls. Proteins were pre-incubated for 5 min then mixed with 7310 nM (nucleotides) 5'-fluorescein-labelled 135mer oligonucleotide (AGCTACCATGCCTGCACGAATTAAGCAATTCGTAATCATGGGTCAAAATCAATCTAAAGTATATATGAGTAAACTTGGTCTGACAGTTACCAATGCTTAATCAGTGAGGCACCTATCTCAGCGATCTGTCTATTT) or a 5'-fluorescein-labelled version of the 60mer used in EMSA in either nuclease protection buffer (20 mM Tris-HCl (pH 7.5), 8% glycerol, 1 mM DTT, 50 mM sodium acetate, 2 mM ATP, 2 mM $MgCl_2$, 0.5 mM $CaCl_2$) or D loop Buffer (see below) supplemented with 1 mM ATP, 1 mM $MgCl_2$, 1 mM $CaCl_2$, in 10 µl reaction volume at 25°C for 10 min. 1 µl (2 U) bovine pancreatic DNaseI (New England Biolabs) was then added for 20 min at 25°C. The samples were deproteinized with 0.125% SDS and 12.5 µg proteinase K for 10 min at 37°C and resolved in 10% polyacrylamide gels in 1X TBE (110 V, 60 min). Gels were imaged on a FLA-9000 scanner (Fujifilm) and quantified with Multi Gauge V3.2 (Fujifilm) or ImageQuant TL (Amersham Biosciences). To calculate the normalized protection relative to RAD-51 alone (Figure 5E) the percentage of DNA binding at different RFS-1/RIP-1:RAD-51 ratios was determined from the native PAGE EMSA experiment in Figure S3B. The percentage of DNA binding and percentage of DNA protected in the EMSA and nuclease protection assays, respectively, were normalized such that the values for RAD-51 alone in each case were 1, and the normalized relative protection for each concentration of RFS-1/RIP-1 determined as the ratio of these values. This accounts for the underestimate in DNaseI deprotection due to the concomitant increase in protein-ssDNA complex formation in the presence of RFS-1/RIP-1.

D-loop formation assays

RAD-51 and RFS-1/RIP-1 were diluted from concentrated stocks into T Buffer (25 mM Tris-HCl (pH 7.5), 10% glycerol, 0.5 mM EDTA (pH 8.0), 50 mM KCl) or D-loop Buffer (10 mM Tris-HCl (pH 7.5), 50 mM KCl, 1 mM DTT) respectively, which were also used in no protein controls. Proteins were pre-incubated for 5 min then mixed with either 50 nM (moles) (Figure 2C, D) or 38.2 nM (moles) (Figure S2E) 5'-fluorescein-labelled 90mer oligonucleotide (AAATCAATCTAAAGTATATATGAGTAAACTTGGTCTGACAGTTACCAATGCTTAATCAGTGAGGCACCTATCTCAGCGATCTGTCTATTT), 1 mM ATP or the indicated nucleotide, 1 mM $CaCl_2$ in D-loop Buffer at 25 °C for 10 min. 2 µl (920 ng) pBluescript SK(–) (460 ng / µl) was then added to bring the final reaction volume to 10 µl and incubated for 15 min. The samples were

deproteinized with 0.1% SDS and 10 μg proteinase K for 10 min at 37 °C and resolved in 0.9% agarose gels in 1X TAE (90 V, 35 min). Gels were imaged on a FLA-9000 scanner (Fujifilm) and quantified with Multi Gauge V3.2 (Fujifilm).

Strand exchange assays

40mer dsDNA was prepared by annealing 5'-fluorescein-labelled 40mer oligonucleotide (TAATACAAAATAAGTAAATGAATAAACAGAGAAAATAAAG) to the complementary unlabelled 40mer oligonucleotide (CTTTATTTTCTCTGTTTATTCATTTACTTATTTTGTATTA) in 50 mM Tris-HCl (pH 7.5), 100 mM NaCl, 10 mM $MgCl_2$, and stored at stock concentration 200 nM (moles). Proteins were diluted from concentrated stocks into T Buffer (25 mM Tris-HCl (pH 7.5), 10% glycerol, 0.5 mM EDTA (pH 8.0), 50 mM KCl), which was also used in no protein controls. Proteins were pre-incubated for 5 min then mixed with 5.6 nM (moles) 150mer oligonucleotide (TCTTATTTATGTCTCTTTTATTTCATTTCCTATATTTATTCCTATTATGTTTTATTCATTTACT TATTCTTTATGTTCATTTTTTATATCCTTTACTTTATTTTCTCTGTTTATTCATTTACTTATTT TGTATTATCCTTATCTTATTTA), 50 mM Tris-HCl (pH 7.5), 1 mM DTT, 100 μg/ml of BSA, 2 mM ATP, 4 mM $CaCl_2$ in 12.5 μl reaction volume at 25 °C for 10 min. 0.5 μl dsDNA stock and 0.5 μl 0.1 M spermidine were then added incubated for 1 h. The samples were deproteinized with 0.1% SDS and 12.5 μg proteinase K at 37 °C and resolved in 10% polyacrylamide gels in 1X TBE (80 V, 1 h 15 min). Gels were imaged on a FLA-9000 scanner (Fujifilm) and quantified with Multi Gauge V3.2 (Fujifilm).

Stopped-flow assays and data analysis

Stopped-flow experiments were performed using an SFM-300 stopped-flow machine (Bio-Logic) fitted with a MOS-200 monochromator spectrometer (Bio-Logic) with excitation wavelength set at 545 nm. Fluorescence measurements were collected with a 550 nm long pass emission filter. The machine temperature was maintained at 25 °C with a circulating water bath.

For all experimental setups, a master mix containing all common reaction components for each of the two syringes was prepared to which variable components were added to generate the mixtures for individual syringes for different experimental conditions. These individual syringe mixtures are indicated in the mixing schemes. Since equal volumes were injected into the mixing chamber from each syringe, the two solutions became mutually diluted. Therefore, all reaction components common to each syringe were prepared at the final concentration, whereas reaction components present in only one syringe were added at twice the desired final concentration. All concentrations quoted represent final concentrations after mixing. Components of each syringe were pre-incubated for 10 min before

the start of experiments to allow the contents to reach equilibrium. Therefore in competition experiments with scavenger DNA, RAD-51-ssDNA filaments pre-formed in the presence of RFS-1/RIP-1 had already undergone remodelling, which is largely completed within 30-60 s, as judged by other experiments.

All reactions were performed in Stopped Flow Buffer (50 mM Tris-HCl (pH 7.5), 5 mM $MgCl_2$, 50 mM NaCl, 2 mM ATP), except in Figure 4E, where ATP was only pre-incubated with DNA, giving a final concentration of 1 mM. All reactions contained 15 nM (moles) 5'-Cy3 fluorescently labelled $(dT)_{43}$ or $(dT)_{23}$ oligonucleotide (Cy3-43mer or Cy3-23mer). Proteins were added directly from concentrated stocks. Unlabelled $(dT)_{43}$ or $(dT)_{23}$ oligonucleotide was used at 1500 nM (moles) (100-fold excess) in competition experiments. For all experiments, controls were also performed for buffer alone with and without DNA to confirm fluorescence signal stability over the time course of the experiments (data not shown). We verified that the theoretical minimum concentration of RAD-51 (320 nM) required to fully coat all molecules of DNA (640 nM nucleotides in stopped-flow) with filaments was sufficient to induce a maximal change in Cy3 fluorescence over longer time courses, whilst sub-saturating concentrations of RAD-51 (<320 nM) gave intermediate changes in Cy3 fluorescence (Figure S4I, J). Successively increasing RAD-51 concentration above 320 nM increased the rate of binding to ssDNA and attainment of equilibrium (Figure S4I). For experiments on pre-formed RAD-51 filaments we employed a saturating concentration of RAD-51 (1 μM), to ensure all ssDNA present was coated by RAD-51 filaments.

Fluorescence measurements for most experiments were collected according to the following protocol: (1) every 0.00005 s from 0-0.05 s; (2) every 0.0005 s from 0.05-0.56 s; (3) every 0.02 s from 0.56-60.54 s. For longer time courses (150 or 300 s), measurements were collected every 0.05 s during stage (3).

For each condition analysed, traces were collected from between three and nine independent reactions (n = 3-9, see individual figure legends for details). For presentation, average traces for each experiment were generated, except for some longer time course (150-300 s) experiments where single traces were analysed. Importantly, all conclusions from this study are made based on the magnitude and rate of changes in fluorescence over time and how these vary with RFS-1/RIP-1 concentration, and therefore the absolute fluorescence values were converted to arbitrary units by a normalization procedure to facilitate comparison. For all experiments the raw data were normalized to the same fluorescence value for the 0 s time point, except for the unlabelled DNA competition experiments (Figure 5, 6G, H) where raw data were normalized differently. In these experiments, an initial increase in fluorescence was observed in most samples, which reached a maximum at approximately 2 s,

particularly for the 43mer experiments. This is also observed for human Rad51 (M.S. and L.K., unpublished data) and therefore represents a systematic artefact of reaction equilibration upon mixing pre-assembled ATP-bound filaments with competitor DNA. Traces were therefore normalized to the same value for Cy3 fluorescence at the 2.01998 s time point and truncated before this to eliminate these artefacts. These adjustments were to aid comparison of protein concentration-dependent trends in the change in fluorescence over time.

For analysis, for all experiments in Figure 4 and 6E, F ten-point moving averages were calculated on each individual normalized trace, which were used to define initial (0 s), final (60.54 s), maximum fluorescence (and corresponding time point) and Δ Cy3 fluorescence values for each experiment. Half-times for different phases were also measured from time points where the fluorescence from moving averages was closest to the value calculated for Δ Cy3 fluorescence midpoints. Average values and associated standard deviations were then calculated. For competition experiments (Figure 5B, C and 6G, H), the relatively small changes in fluorescence observed meant the data was too noisy for analysis from moving averages for Δ Cy3 fluorescence and half-times in this way. For these experiments, Δ Cy3 fluorescence values were calculated on each individual normalized trace as the difference between the mean fluorescence values measured across all time points from 1.01998 to 2.99998 s (start value, on either side of the maximum at ~2 s) and 58.54 to 60.54 s (end value). Average values and associated standard deviations were then calculated. For data analysis of 300 s reactions (Figure S4M, N), estimates of fluorescence values at a given time point were determined as the mean fluorescence values measured across all time points ±1 s on individual traces. Positive and negative Δ Cy3 fluorescence values represent increases and decreases in fluorescence respectively.

Electron microscopy

For attempted filament reconstructions, 8 μM RAD-51 was incubated ± 3 μM RFS-1/RIP-1 with M13 ssDNA (USB) at RAD-51:DNA ratio 80:1 (w/w) in 25 mM triethanolamine-HCl (pH 7.2), 1.25 mM ATP, 1.25 mM NaF, 1.25 mM $Al(NO_3)_3$, 2 mM magnesium acetate at 30 °C for 30 min. Samples were applied to glow-discharged continuous carbon-coated grids, negatively stained by 2% uranyl acetate, and imaged on an FEI Tecnai T12 microscope at 80 keV with a nominal magnification of ×30,000. Images were recorded on Kodak SO163 film and digitized using a Nikon Coolpix 8000 scanner at a raster of 4.16 Å per pixel. The EMAN software package was used to extract filament images from micrographs, and the SPIDER software package was used for most image processing.

Immuno-gold labeling experiments were performed similarly to described previously (Shahid et al., 2014). Anti-FLAG antibody (Sigma F3165) was conjugated to 20-nm gold particles using the InnovaCoat Gold Conjugation Mini kit (Innova Biosciences). 1 μM RAD-51 ± 0.5 μM RFS-1/RIP-1 and 1 μM (nucleotides) of linearized PhiX ssDNA (gift of Michael Mcilwraith and Stephen West) were co-incubated in 20 mM Triethanolamine-HCl (pH 7.5), 8% glycerol, 1 mM DTT, 50 mM sodium acetate, 2 mM $MgCl_2$, 2 mM ATP for 10 min at 25 °C, before incubation with anti-FLAG-20-nm gold for a further 30 min at 25 °C. Samples were imaged by negative stain as described (Shahid et al., 2014). For quantification, five positions were counted for each grid (at the four cardinal directions and one centrally). Discrete filaments >50 nm in length (RAD-51: n = 644; RAD-51 + RFS-1/RIP-1: n = 588) were counted from each of 93 (RAD-51) or 112 (RAD-51 + RFS-1/RIP-1) fields of view, each an image of size 2120 nm x 1511 nm at nominal magnification 67,000X, and scored for number bound with anti-FLAG-20-nm gold on the end or along the filament length. Filament length was also determined for filaments associated with anti-FLAG-20-nm gold. Statistical analysis was performed in GraphPad Prism 6 (GraphPad Software).

Single molecule FRET (smFRET)

DNA oligonucleotides were ordered from Operon, and labelled with Cy3 or Cy5 using an NSH-ester modification (GE healthcare) reaction with amino-modified C6-dT. The sequence of the Cy3 anchor DNA is 5′-Cy3-CGCGAGGAATGGATGTAGGG-biotin-3′. The sequence of the Cy5 poly-dT7 DNA is 5′-CCCTACATCCATTCCTCGCGTTTTTT(Cy5-T)TTTTTTTTTTTTTTTTTTTTTTTTTT TTTTTTTTTTTTTTTTTTTTTTTTTTTTTTTTTTTTTT-3′. The labelling reaction was performed with 1 nmol DNA dissolved in 44 μL of 100 mM sodium tetraborate buffer, pH 8.5, and 10 nmol of Cy3 or Cy5 dissolved in 7 μL of DMSO. The reaction was incubated at room temperature overnight to obtain ≥80% labelled DNA. Labelled and unlabelled DNA fractions were separated by reverse phase HPLC chromatography on an analytical C18-column. Purified oligonucleotides were stored at –20 °C in 10 mM Tris-HCl, pH 8.0.

Single molecule FRET experiments were performed as previously described (Senavirathne et al., 2012). Briefly, quartz microscope slides were passivated with methoxy-PEG-SVA (M_r = 5,000; Laysan Bio Inc.) doped with 10% biotin-PEG-SVA (M_r = 3,400; Laysan Bio Inc.) to minimize non-specific binding to the surface. First, the reaction chamber was incubated with 0.2 mg/ml BSA (Sigma) in T50 buffer (50 mM Tris-HCl (pH 7.0), 50 mM NaCl) for 10 min, and then excess BSA was washed out with T50 buffer. Second, streptavidin (1 mg/ml in T50 buffer) was incubated for 10 min, and then

excess streptavidin was washed out with buffer (50 mM Tris-HCl (pH 7.5), 50 mM NaCl, 5 mM $MgCl_2$, 2 mM Trolox). Third, the ssDNA construct was surface immobilized. The DNA construct was prepared by annealing 1 μM each of the 3′-biotin 5′-Cy3 anchor and the Cy5 polydT7 DNA (Figure 7) in buffer (50 mM Tris-HCl (pH 7.5), 50 mM NaCl, 5 mM $MgCl_2$, 2 mM Trolox) by heating to 90 ˚C for 45 s and cooling to room temperature over 15 min, followed by serial dilutions to 25-50 pM DNA for surface immobilization. Excess DNA was washed out after a 10 min incubation. Finally, the proteins for each experiment (RAD-51, RFS-1/RIP-1, RFS-1(K56A)/RIP-1, and RFS-1(E138A)/RIP-1, 1 μM each) were introduced in imaging buffer (50 mM Tris-HCl (pH 7.5), 50 mM NaCl, 5 mM $MgCl_2$, 2 mM ATP, 0.2 mg/mL BSA, 2 mM Trolox to prevent photoblinking of the dyes, and an oxygen scavenger system consisting of 2.5 mM 3,4-dihydroxybenzoic acid (PCA) (Sigma) and 250 nM protocatechuate dioxygenase (PCD) (Sigma) to minimize photobleaching). Data was acquired on a home-built, prism-based single-molecule Total Internal Reflection Fluorescence (smTIRF) microscope at 30 ms time resolution. Movies were recorded for 5 min from 10 different areas of the slide. Apparent energy transfer efficiencies were calculated as FRET = $I_A/(I_D + I_A)$, where I_A is the acceptor intensity and I_D is the donor intensity.

ATPase assays

0.5 μM of RFS-1/RIP-1 or RecA (New England Biolabs) were mixed with or without 100 nM (moles) φX174 viron ssDNA (New England Biolabs) in 20 mM Triethanolamine-HCl (pH 7.5), 8% glycerol, 1 mM DTT, 50 mM sodium acetate, 2 mM $MgCl_2$, 2 mM ATP, 0.6 μCi γ[^{32}P]-ATP (6000 Ci/mmol) in 60 μl total volume at 37 °C. 8 μl samples were taken at the indicated time points and the reaction stopped with 4 μl 0.5 M EDTA (pH 8.0). 1.5 μl of each sample were loaded onto Polygram CEL 300 PEI thin layer chromatography plates (Macherey Nagel 801053), separated in 0.5 M LiCl, 1 M formic acid, and imaged using a storage phosphor screen (Amersham Biosciences) and a Storm 840 molecular imager (Molecular Dynamics).

Yeast two-hybrid

Yeast two-hybrid was performed as described previously (Boulton et al., 2002). N-terminal fusions of the DNA binding (DB) and transactivation (AD) domains of yeast Gal4 to RFS-1 and RIP-1 were cloned using the Gateway system and site-directed mutagenesis performed using the QuikChange Site-Directed Mutagenesis Kit (Stratagene).

GST pull-downs from human cells

N-terminal GST and FLAG fusions of RFS-1 and RIP-1 were cloned using the Gateway system under the control of the CMV promoter. Plasmids were transfected into 5 x 10^6 human 293T cells with Lipofectamine 2000 and cells were harvested 24 h later by scraping in Benzonase Lysis Buffer (20 mM Tris-HCl (pH 7.5), 40 mM NaCl, 2 mM $MgCl_2$, 0.5% NP-40, 50 U / ml benzonase (Novagen), cOmplete, EDTA-free protease inhibitor cocktail tablets (Roche) (1 tablet per 25 ml buffer)). After incubation for 10 min on ice, NaCl concentration was increased to 150 mM and the lysate rested on ice for a further 30 min with occasional vortexing. The lysate was cleared and the soluble fraction (4 mg protein) applied to 30 µl glutathione sepharose 4 fast flow beads (GE Healthcare 17-5132) by rotating overnight at 4 °C. 10% of the soluble fraction was saved as the input sample. The beads were washed three times with IP Buffer (25 mM Tris-HCl (pH 7.5), 10% glycerol, 1.5 mM DTT, cOmplete, EDTA-free protease inhibitor cocktail tablets (1 tablet per 50 ml buffer), 0.25% NP-40, 150 mM NaCl) before boiling for 5 min in 30 µl sample buffer to release pulled-down proteins. 40 µg input and 10 µl pull-downs were analysed by western blot with anti-GST (Santa Cruz 459) and anti-FLAG-HRP (Sigma A8592) antibodies.

***C. elegans* strains and methods**

Strains were maintained using standard techniques on nematode growth medium agarose plates supplemented with nystatin. N2 (Bristol) wild type and *rfs-1(ok1372)* and *helq-1(tm2134)* mutant strains were as described previously (Ward et al., 2007; Ward et al., 2010). A mutant allele in the open reading frame R01H10.5, *rip-1(tm2948)*, was obtained from the Japanese National BioResource Project for *C. elegans*, which is a 668 bp deletion between nucleotides 12202 and 12870 in cosmid R01H10 that completely removes exons 2 and 3 and generates a frameshift that introduces a premature stop codon in exon 4. This allele is predicted to encode a truncated protein of 68 amino acids. The strain was backcrossed to N2 six times before use. The *rip-1(tm2948) helq-1(tm2134)* double mutant was maintained as a *rip-1(tm2948)* heterozygote by balancing with the hT2(gfp) balancer. Homozygote double mutants were selected by picking worms not expressing GFP in the pharynx.

DNA damage sensitivity assays

Nitrogen mustard (HN2, mechlorethamine; Sigma) sensitivity assays were performed in liquid culture by incubating randomly picked young adult animals in 2 ml total volume S Medium, the indicated drug concentration and 50 µl concentrated HB101 bacteria, at room temperature for 19 h on a slow shaker protected from light. Animals were washed in PBS, recovered for 3 h on OP50 seeded

MYOB plates, then transferred individually to single plates and left to lay for 4 h at 20 °C. 12 worms per strain per dose were scored. The parental worms were then removed and the eggs laid counted immediately and after 2 days at 20 °C, and the percentage of embryonic survival scored. Ionizing irradiation was carried out using a caesium-137 source on young adult animals on seeded plates. Plates were incubated for 22 h at 20 °C before animals were transferred individually to single plates and scored as for HN2.

Immunofluorescence

Randomly picked gravid adult hermaphrodites were treated with nitrogen mustard (HN2, 200 μM) and cisplatin (CDDP, 180 μM) for 19 h in liquid culture and hydroxyurea (HU, 40 mM) on OP50 seeded MYOB plates containing HU for 16 h. Ionizing irradiation and UV-C (254 nm) treatment were performed on seeded plates. After treatment, animals were transferred to fresh seeded plates and allowed to recover (HN2, 16 h; CDDP, 18 h; HU, 16 h; IR, 4 h; UV-C, 2h). Worms were washed twice in PBS, transferred to poly-L-lysine coated slides and germlines dissected. Germlines were fixed in 4% paraformaldehyde for 10 min, permeabilized in TBSBT (1X TBS, 0.5% BSA, 0.1% Triton X-100) for 20 min then washed 3 x 30 min with TBSB (1X TBS, 0.5% BSA). Germlines were stained with a rabbit anti-RAD-51 antibody (gift of Anton Gartner) diluted 1:500 in TBSB overnight at 4 °C in a humid chamber. Germlines were washed 3 x 30 min with TBSB, then incubated with secondary antibodies (anti-rabbit Cy3 (Sigma) or anti-rabbit Alexa488 (Life Technologies)) diluted 1:10000 in TBSB for 2 h at room temperature in a dark humid chamber. The germ lines were washed 1 x 30 min with TBSB, stained 5 min with DAPI (1 μg/ml in TBSB), washed 3 x 30 min with TBSB, then mounted with Vectashield. Images were acquired by Deltavision microscopy and analysed as described (Martin et al., 2005). Foci were analysed from 10-20 nuclei from each of 5-25 animals.

Germline apoptosis assays

Randomly picked young adult worms were incubated in 33 μM SYTO12 dye (Molecular Probes) in 50 μl M9 for 3 h at 20 °C protected from the light. Worms were then washed with PBS, incubated for 1 h on OP50 seeded MYOB plates in the dark to purge SYTO12-stained bacteria in the gut. Animals were then mounted in 10 mM levamisole on a 4% agarose pad and imaged and scored using an excitation wavelength of 488 nm on an Axioskop2 microscope (Zeiss).

Supplemental References

Frigola, J., Remus, D., Mehanna, A., and Diffley, J.F. (2013). ATPase-dependent quality control of DNA replication origin licensing. Nature. *495*, 339-343.

Senavirathne, G., Jaszczur, M., Auerbach, P.A., Upton, T.G., Chelico, L., Goodman, M.F., and Rueda, D. (2012). Single-stranded DNA Scanning and Deamination by APOBEC3G Cytidine Deaminase at Single Molecule Resolution. J. Biol. Chem. *287*, 15826-15835.

Shahid, T., Soroka, J., Kong, E.H., Malivert, L., McIlwraith, M.J., Pape, T., West, S.C., and Zhang, X. (2014). Structure and mechanism of action of the BRCA2 breast cancer tumor suppressor. Nat. Struct. Mol. Biol. *21*, 962-968.
