# Supplementary material for: Rad51 Paralogs Remodel Pre-synaptic Rad51 Filaments to Stimulate Homologous Recombination
Source: Cell. 2015 Jul 16;162(2):271–86. doi: 10.1016/j.cell.2015.06.015 (PMC4518479; doi:10.1016/j.cell.2015.06.015)
Supplement: Document S2. Article plus Supplemental Information [file mmc2.pdf]

Cell

Article

Rad51 Paralogs Remodel Pre-synaptic Rad51 Filaments to Stimulate Homologous Recombination

Graphical Abstract

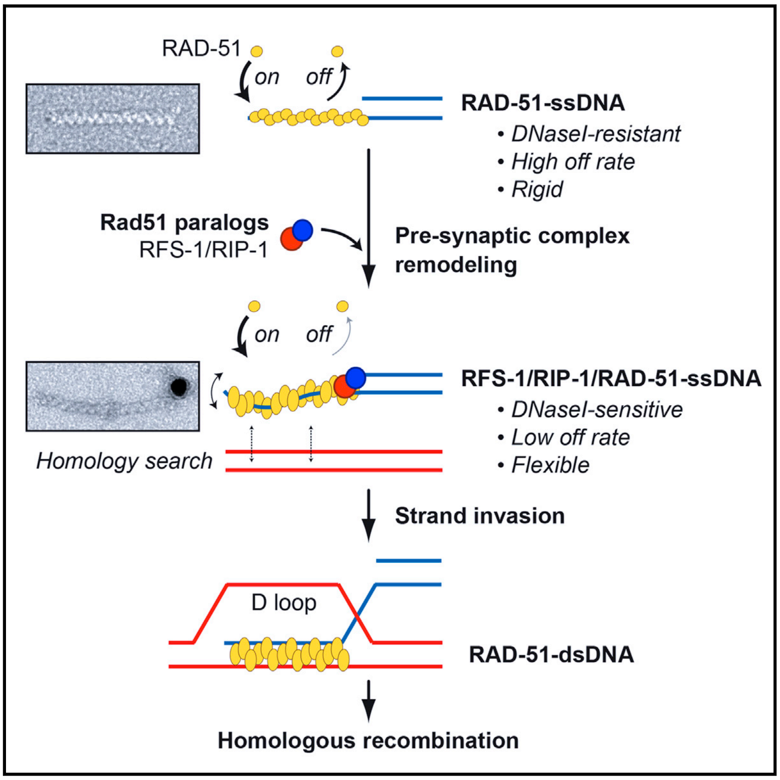

Highlights

- A Rad51 paralog complex, RFS-1/RIP-1, binds RAD-51-ssDNA pre-synaptic filaments
- RFS-1/RIP-1 stabilizes the filament by reducing RAD-51 dissociation from ssDNA
- RFS-1/RIP-1 remodels the pre-synaptic complex to a more "open," flexible conformation
- Pre-synaptic complex remodeling stimulates strand exchange and recombination

Authors

Martin R.G. Taylor, Mário Špírek, Kathy R. Chaurasiya, ..., David Rueda, Lumir Krejci, Simon J. Boulton

Correspondence

lkrejci@chemi.muni.cz (L.K.),
simon.boulton@crick.ac.uk (S.J.B.)

In Brief

Rad51 paralogs, known tumor suppressors, induce remodeling at the tips of Rad51-ssDNA filaments to form an open and flexible conformation that promotes homologous recombination.

CrossMark

Taylor et al., 2015, Cell *162*, 271–286
July 16, 2015 ©2015 The Authors
http://dx.doi.org/10.1016/j.cell.2015.06.015

CellPress

Cell

Rad51 Paralogs Remodel Pre-synaptic Rad51 Filaments to Stimulate Homologous Recombination

Martin R.G. Taylor,[1] Mário Špírek,[2,3] Kathy R. Chaurasiya,[5] Jordan D. Ward,[1,6] Raffaella Carzaniga,[7] Xiong Yu,[8] Edward H. Egelman,[8] Lucy M. Collinson,[7] David Rueda,[5] Lumir Krejci,[2,3,4,*] and Simon J. Boulton[1,*]
[1]DNA Damage Response Laboratory, Clare Hall Laboratory, The Francis Crick Institute, South Mimms EN6 3LD, UK
[2]Department of Biology, Masaryk University, 62500 Brno, Czech Republic
[3]International Clinical Research Center, St. Anne's University Hospital in Brno, 62500 Brno, Czech Republic
[4]National Centre for Biomolecular Research, Masaryk University, 62500 Brno, Czech Republic
[5]Section of Virology, Single Molecule Imaging Group and MRC Clinical Sciences Centre, Department of Medicine, Imperial College London, London W12 0NN, UK
[6]UCSF-Mission Bay, Genentech Hall S574, San Francisco, CA 94158, USA
[7]Electron Microscopy Science Technology Platform, Lincoln's Inn Fields Laboratory, The Francis Crick Institute, London WC2A 3LY, UK
[8]Department of Biochemistry and Molecular Genetics, University of Virginia School of Medicine, Charlottesville, VA 22908, USA
*Correspondence: lkrejci@chemi.muni.cz (L.K.), simon.boulton@crick.ac.uk (S.J.B.)
http://dx.doi.org/10.1016/j.cell.2015.06.015
This is an open access article under the CC BY license (http://creativecommons.org/licenses/by/4.0/).

SUMMARY

Repair of DNA double strand breaks by homologous recombination (HR) is initiated by Rad51 filament nucleation on single-stranded DNA (ssDNA), which catalyzes strand exchange with homologous duplex DNA. BRCA2 and the Rad51 paralogs are tumor suppressors and critical mediators of Rad51. To gain insight into Rad51 paralog function, we investigated a heterodimeric Rad51 paralog complex, RFS-1/RIP-1, and uncovered the molecular basis by which Rad51 paralogs promote HR. Unlike BRCA2, which nucleates RAD-51-ssDNA filaments, RFS-1/RIP-1 binds and remodels pre-synaptic filaments to a stabilized, "open," and flexible conformation, in which the ssDNA is more accessible to nuclease digestion and RAD-51 dissociation rate is reduced. Walker box mutations in RFS-1, which abolish filament remodeling, fail to stimulate RAD-51 strand exchange activity, demonstrating that remodeling is essential for RFS-1/RIP-1 function. We propose that Rad51 paralogs stimulate HR by remodeling the Rad51 filament, priming it for strand exchange with the template duplex.

INTRODUCTION

Homologous recombination (HR) is an essential mechanism for the repair of DNA double strand breaks (DSBs) and damaged replication forks. HR is initiated at single-stranded DNA (ssDNA) exposed at nucleolytically processed DSB ends or post-replicative ssDNA gaps by the exchange of the ssDNA binding protein RPA for the recombinase enzyme Rad51, which forms helical nucleoprotein filaments on ssDNA. Rad51-ssDNA filaments probe for homologous duplex DNA and catalyze strand invasion, displacing the non-complementary strand of the template duplex to form a displacement loop (D loop) structure. Unloading of Rad51 from double-stranded DNA (dsDNA) permits the initiation of repair DNA synthesis and the resulting joint molecules are processed by various enzymes to complete the repair reaction (Chapman et al., 2012; San Filippo et al., 2008).

HR is regulated by mediator proteins, including BRCA2, Rad54, and the family of Rad51 paralogs (San Filippo et al., 2008), which appear to act as positive regulators at different steps of the HR reaction. For example, BRCA2 facilitates Rad51 nuclear localization (Jeyasekharan et al., 2013; Martin et al., 2005), RPA displacement from ssDNA, and Rad51 filament nucleation (Jensen et al., 2010; Liu et al., 2010; Thorslund et al., 2010), whereas Rad54 unloads Rad51 from dsDNA to promote repair DNA synthesis (Solinger et al., 2002). However, the molecular mechanism underlying the stimulation of HR by the Rad51 paralogs has remained elusive.

Rad51 paralog proteins share sequence similarity to Rad51 and possess homology extending across the motor ATPase fold, including the highly conserved Walker A and Walker B boxes, but not the N-terminal helix-hairpin-helix motif (Lin et al., 2006). Five Rad51 paralogs have been identified in mammalian and avian species, which interact with one another to form two constitutive complexes, RAD51B-RAD51C-RAD51D-XRCC2 (BCDX2) and RAD51C-XRCC3 (CX3) (Masson et al., 2001; Yonetani et al., 2005), and in budding yeast two Rad51 paralogs constitute the Rad55-Rad57 heterodimer (Sung, 1997). In addition, the budding and fission yeast Shu complexes (Martín et al., 2006; Shor et al., 2005) contain proteins lacking detectable sequence homology to Rad51 but contain Rad51-like folds as determined by crystal structures (Sasanuma et al., 2013; Tao et al., 2012) or show very limited homology centered on the Walker B motif, such as budding/fission yeast Psy3/Rdl1 (Martín et al., 2006). The high degree of conservation around these short Walker motifs among the Rad51 paralog family reflects their functional importance in conferring resistance to DNA damage and the integrity of the Rad51 paralog complexes

in mammalian cells (French et al., 2003; Gruver et al., 2005; Wiese et al., 2006; Yamada et al., 2004).

Ablation of Rad51 paralogs leads to severe HR defects, DNA damage sensitivity, chromosome abnormalities, and defective Rad51 nuclear focus formation after DNA damage, suggestive of a major function at an early stage in the HR reaction (Chun et al., 2013; French et al., 2002; Gasior et al., 1998; Godthelp et al., 2002; Hays et al., 1995; Johnson et al., 1999; Pierce et al., 1999; Rattray and Symington, 1995; Takata et al., 2000, 2001). Like *BRCA2* and *PALB2*, which are mutated in Fanconi anemia and breast and ovarian cancer (Howlett et al., 2002; Lancaster et al., 1996; Rahman et al., 2007; Reid et al., 2007; Wooster et al., 1995; Xia et al., 2007), biallelic germline mutations in *RAD51C* cause a severe form of Fanconi anemia (Vaz et al., 2010), whereas monoallelic inheritance of mutations in *RAD51C* and *RAD51D*, and *RAD51B*, predispose individuals to ovarian and breast cancer, respectively (Golmard et al., 2013; Loveday et al., 2011; Meindl et al., 2010), demonstrating an important tumor suppressor function for HR mediators.

The budding yeast Rad55-Rad57 complex (Sung, 1997) and a sub-complex of the human BCDX2 complex, RAD51B-RAD51C (Sigurdsson et al., 2001), have been purified as heterodimers and despite lacking intrinsic recombinase activity they stimulate strand exchange by Rad51 in vitro. Rad55-Rad57 and the Shu complex form co-complexes with Rad51-ssDNA filaments (Liu et al., 2011a; Sasanuma et al., 2013), which in the case of Rad55-Rad57 renders the pre-synaptic complex resistant to disruption by the anti-recombinase Srs2 (Liu et al., 2011a). However, the mechanism by which Rad51 paralogs directly stimulate the recombinase activity of Rad51 has remained enigmatic for many years (Sung, 1997). Additionally, whether the Rad51 paralogs confer any intrinsic stabilization or alteration in the structural properties of the pre-synaptic complex is unknown, as is the mechanistic importance of their conserved Walker motifs.

In this study, we report the identification and characterization of a Rad51 paralog complex, RFS-1/RIP-1, from *Caenorhabditis elegans*. RFS-1/RIP-1 is required for HR and RAD-51 focus formation at DNA damage sites in vivo, and it stimulates the recombinase activity of RAD-51 and associates directly with RAD-51 filaments in vitro. Using multiple biochemical and biophysical approaches, we demonstrate that RFS-1/RIP-1 structurally remodels the pre-synaptic RAD-51-ssDNA filament to a stabilized, "open," flexible conformation, which facilitates strand exchange with the template duplex. Using specific mutants in the Walker boxes of RFS-1, which are compromised for stimulating strand exchange, we demonstrate that filament remodeling is critical for RFS-1/RIP-1 mediator activity. Collectively, this defines the underlying mechanism of HR stimulation by Rad51 paralogs.

RESULTS

Identification of a Heterodimeric Rad51 Paralog Complex in *C. elegans*

To investigate the mechanism of action of Rad51 paralogs in promoting HR, we utilized the simplified model system *C. elegans*, which encodes a single canonical Rad51 paralog, RFS-1 (Figure 1A). We previously showed that *rfs-1* mutant strains are sensitive to DNA damage, defective for RAD-51 focus formation at stalled replication forks, and exhibit meiotic defects when combined with *helq-1* mutations (Ward et al., 2007, 2010). However, attempts to purify RFS-1 yielded largely insoluble protein intractable to biochemical analysis (Figure S1A). Since Rad51 paralogs in other organisms function as complexes, we reasoned that RFS-1 may require a binding partner for optimal function. Using a yeast two-hybrid screen (Boulton et al., 2002), we identified an orphan protein encoded by R01H10.5 (Uniprot ID code Q21621) that we named RIP-1 (RFS-1 interacting protein) (Figure 1A), which interacted with RFS-1 by yeast two-hybrid (Figure 1B), glutathione S-transferase (GST) pulldowns of *C. elegans* RFS-1 and RIP-1 expressed in human cells (Figure 1C), and FLAG co-immunoprecipitation (co-IP) from yeast cells (Figure 2A). Although RFS-1 interacts with RAD-51 in yeast two-hybrid, no interaction between RAD-51 and RIP-1 was detectable (Figure S1B).

RIP-1 bears no obvious sequence homology with Rad51 family proteins, but does contain a sequence resembling a Walker B motif (Figure 1A), similar to the divergent yeast Rad51 paralog Psy3 that adopts a Rad51-like fold (Martín et al., 2006; Sasanuma et al., 2013; Tao et al., 2012). Since Walker B motifs mediate protein-protein interactions in human Rad51 paralog complexes (Wiese et al., 2006), we tested if this dependency is conserved for the RFS-1/RIP-1 protein-protein interaction. Mutation of the second position valine in the Walker B box of either protein (RFS-1 V135E or RIP-1 V128E) was sufficient to completely abolish the interaction in yeast two-hybrid (Figures 1A and 1C), suggesting that the Walker B motifs mediate the RFS-1/RIP-1 interaction interface. Notably, when these mutants were expressed in yeast, FLAG-tagged RIP-1 was expressed but was not detectable after co-IP (Figure S1C), suggesting RFS-1 and RIP-1 are interdependent for their solubility, consistent with the inability to purify RFS-1 in isolation (Figure S1A). In contrast, mutation of other residues, including RFS-1 lysine-56 and glutamate-138 in the Walker A and B boxes, respectively, conferred weakened yeast two-hybrid interactions but were still permissive for co-IP (Figures 1C and S1C), suggestive of abnormal, but intact, protein complexes.

A *rip-1* deletion mutant (*tm2948*) was found to phenocopy *rfs-1* mutants. Like *rfs-1* mutants, *rip-1*-deficient strains are defective for RAD-51 focus formation after treatment with DNA-damaging agents that stall replication forks (Figure 1D), are sensitive to DNA damage (Figures S1D and S1E), and display elevated germline apoptosis after treatment with interstrand crosslinking agents (Figure S1F) (Ward et al., 2007). *rip-1* mutants also phenocopy the meiotic HR defects of *rfs-1* strains (Ward et al., 2010), exhibiting elevated frequencies of males (Figure S1G) and synthetic lethality with *helq-1*, which is associated with persistent meiotic RAD-51 foci (Figures S1H–S1J). These observations suggest that RIP-1 likely represents a highly divergent RAD-51 paralog that functions as a complex with RFS-1. The existence of non-canonical Rad51 paralogs in other organisms is not unprecedented: yeast cells encode the Shu complex (Martín et al., 2006; Shor et al., 2005), which functions in HR, and human cells encode an ATPase-sharing homology with the archael recombinase RadA, SWSAP1, required for

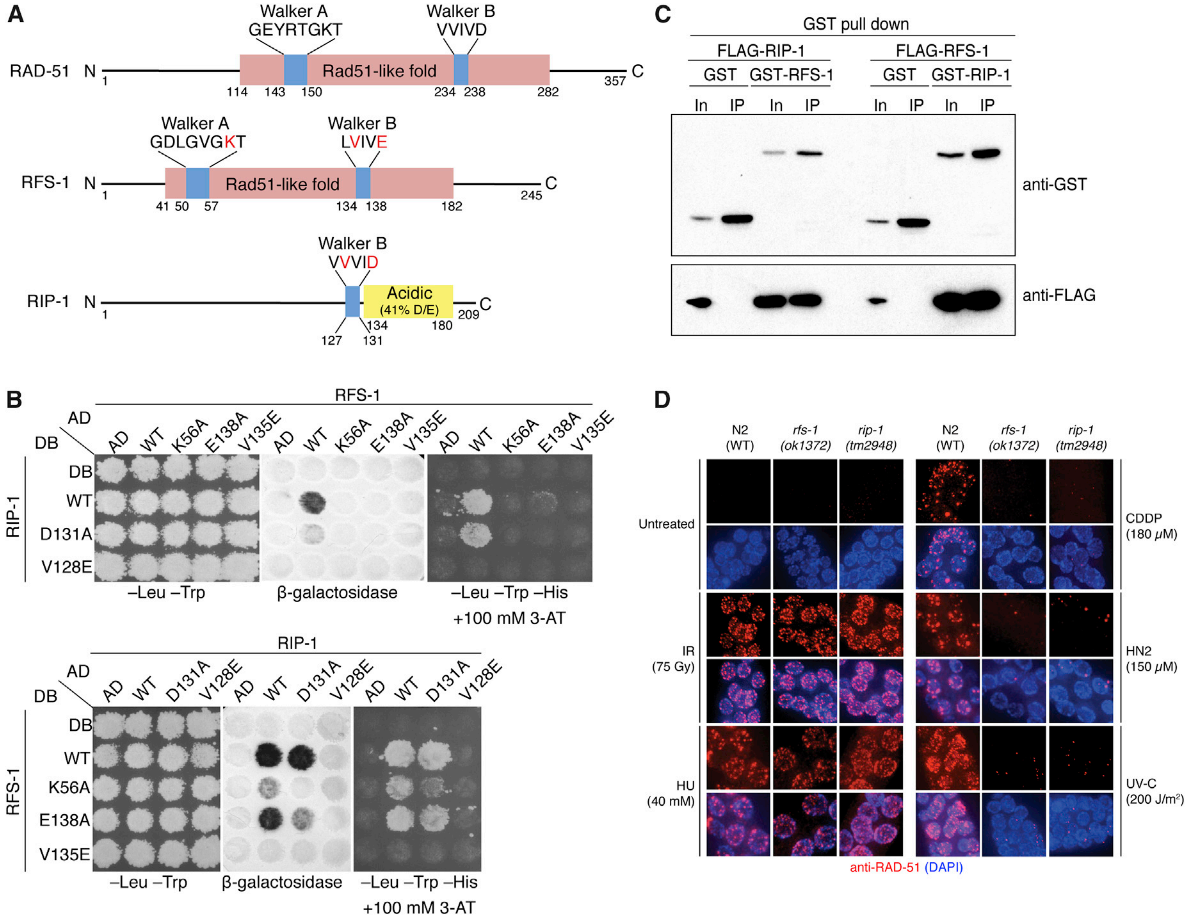

Figure 1. RIP-1 Is a Highly Divergent Rad51 Paralog that Forms a Complex with RFS-1

(A) Schematics of RAD-51, RFS-1, and RIP-1. RAD-51 and RFS-1 are homologous in their central Rad51-like folds (pink). Walker A and B motifs (blue) are annotated, and residues examined by mutagenesis are indicated (red). RIP-1 contains an acidic region (yellow) of unknown function.

(B) RFS-1 and RIP-1 interact via ATPase motifs in reciprocal yeast two-hybrid assays, indicated by positive β-galactosidase expression and survival on media containing 3-aminotriazole (3-AT) in the absence of histidine. Growth on media lacking leucine and tryptophan is a positive control for plasmid transfection.

(C) GST pull-downs of *C. elegans* RFS-1 and RIP-1 expressed in human 293T cells. In, input. IP, pull-down.

(D) RAD-51 immunofluorescence (red) in mitotic nuclei of worm germlines from the indicated genotypes. DNA is stained with DAPI (blue). IR, ionizing radiation. HU, hydroxyurea. CDDP, *cis*-platin. HN2, nitrogen mustard. UV-C, ultraviolet light.

See also Figure S1.

HR, Rad51 focus formation, and DNA damage resistance (Liu et al., 2011b).

RFS-1/RIP-1 Binds ssDNA and Stimulates RAD-51 Recombinase Activity

To investigate the biochemical properties of RFS-1/RIP-1, we co-purified recombinant proteins from budding yeast cells (Figure 2A), which migrated with a molecular weight of approximately 70 kDa on size-exclusion chromatography (Figure S2A), consistent with a 1:1 heterodimer. Electrophoretic mobility shift assays (EMSA) demonstrated that RFS-1/RIP-1 weakly binds ssDNA, but not dsDNA, in a nucleotide-independent manner to form a discrete slow-migrating protein-DNA complex in native polyacrylamide gels (Figures 2B and S2B). Although RFS-1/RIP-1 contains Walker motifs, there was little detectable ATPase activity over the background, suggesting that the RFS-1/RIP-1 complex may lack intrinsic ATPase activity (Figure S2C). However, it remains possible that the optimal biochemical condition and/or the appropriate substrate for RFS-1/RIP-1 ATPase activity are yet to be identified.

To assess how RFS-1/RIP-1 might regulate the HR reaction, we performed strand exchange and D loop formation assays. The nematode RAD-51 is an extremely weak recombinase in the absence of mediators even when tested at a wide range of protein-to-DNA ratios (Petalcorin et al., 2006). Nevertheless, addition of sub-stoichiometric concentrations of RFS-1/RIP-1 relative to RAD-51 caused a dramatic stimulation of both D loop formation (Figure 2C) and strand exchange activities (Figure S2D), which was dependent on the presence of the nucleotide co-factor ATP (Figure 2D).

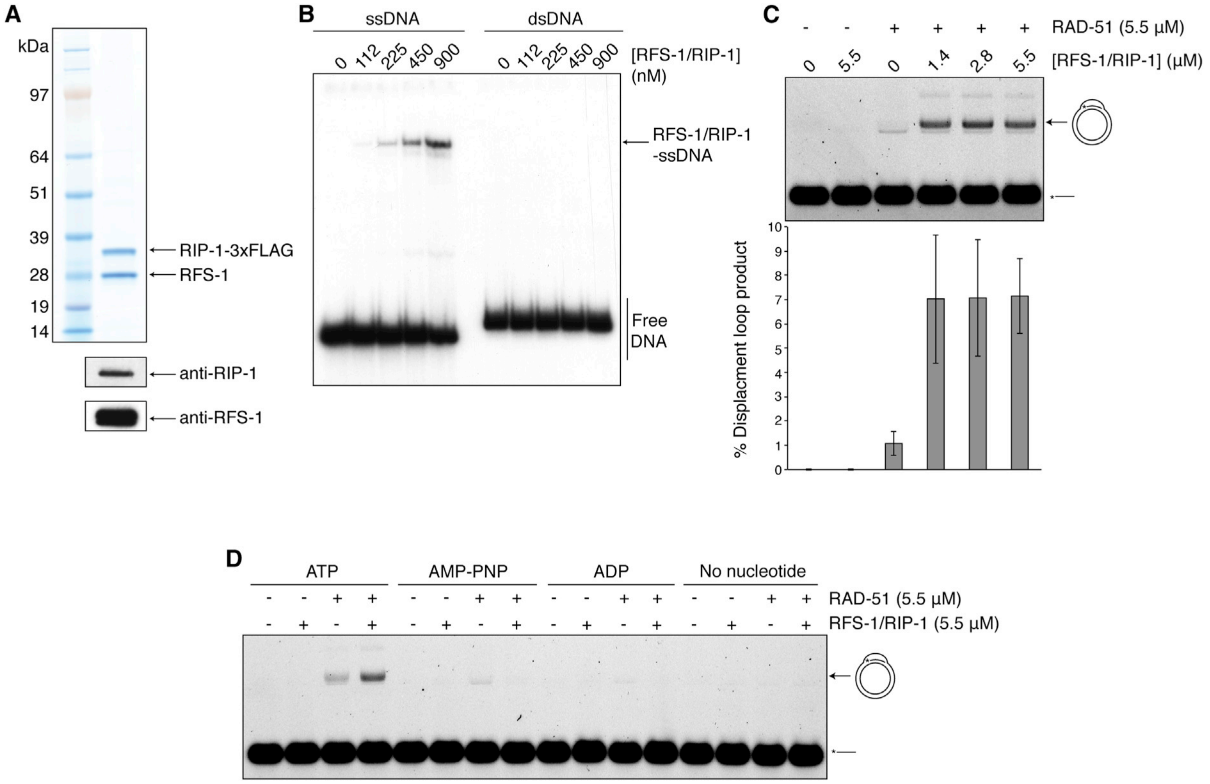

Figure 2. Biochemical Properties of RFS-1/RIP-1 Complex

(A) Purification of recombinant RFS-1/RIP-1 from yeast cells by FLAG immunoprecipitation. Western blots confirm the identity of the two bands.

(B) EMSA showing RFS-1/RIP-1 binding to ssDNA, but not to dsDNA (60-mer).

(C) RFS-1/RIP-1 stimulates D loop formation by RAD-51. Error bars indicate SD (n = 4).

(D) RFS-1/RIP-1 mediator activity requires ATP.

See also Figure S2.

RFS-1/RIP-1 Binds to RAD-51-ssDNA Filaments

To investigate the mechanism by which RFS-1/RIP-1 stimulates RAD-51 recombinase activity, we first assessed how RFS-1/RIP-1 influences RAD-51-ssDNA filament properties in native polyacrylamide gels. RAD-51-ssDNA complexes resolve as fast-migrating smears, representing filaments (Figure 3A) that were only clearly observed in the presence of ATP, and not in the presence of ATPγS, AMP-PNP, or ADP or in the absence of nucleotide (Figure S3C; data not shown). When RAD-51-ssDNA filaments were co-incubated with RFS-1/RIP-1, the resulting nucleoprotein complex migrated more slowly with a concomitant reduction in the amount of free unbound ssDNA, above the additive value predicted from mixing the two proteins (Figure 3A). The effects observed were independent of the relative order of incubation of the protein and DNA components (Figure S3A), occurred at sub-stoichiometric quantities of RFS-1/RIP-1 (Figure S3B), and were specific to RFS-1/RIP-1 (Figure S3D).

We also assessed protein-DNA complexes resolved in agarose gels after glutaraldehyde crosslinking, and this permitted detection of RAD-51-ssDNA filaments in the presence of ATP, ADP, or AMP-PNP and in the absence of nucleotide or magnesium ions (Figures 3B and S3E). RFS-1/RIP-1 caused a greater than additive increase in the proportion of protein-DNA complexes in the presence of any nucleotide. In contrast, the opposite effect was observed in the absence of nucleotide and magnesium, where RFS-1/RIP-1 reduced the proportion of protein-DNA complexes at equilibrium, indicating that the effect of RFS-1/RIP-1 is nucleotide dependent.

Upon co-incubation with RAD-51, we noted that the discrete RFS-1/RIP-1-ssDNA species observed in polyacrylamide gels (Figures 3A, S3A, and S3B) were diminished, suggesting RFS-1/RIP-1 may associate with the filaments. Indeed, retardation of the mobility of the RAD-51-ssDNA filaments formed in the presence of RFS-1/RIP-1 occurred when co-incubated with anti-FLAG antibodies, which bind the FLAG epitope at the RIP-1 C terminus (Figure 3C). Additionally, RFS-1/RIP-1 association with the RAD-51-ssDNA filaments was directly observed using electron microscopy (EM), by incubating with anti-FLAG antibodies conjugated to 20-nm gold particles (Figure 3D). Gold particle binding to the filament was specifically observed in the presence of RFS-1/RIP-1 (Figures 3D and 3E) and was enriched at the filament ends (Figure 3F), suggesting RFS-1/RIP-1 may preferentially cap filaments. No significant differences in filament length were observed among gold particle-bound filaments (RAD-51: 100 ± 31 nm; RAD-51 + RFS-1/RIP-1: 102 ± 40 nm)

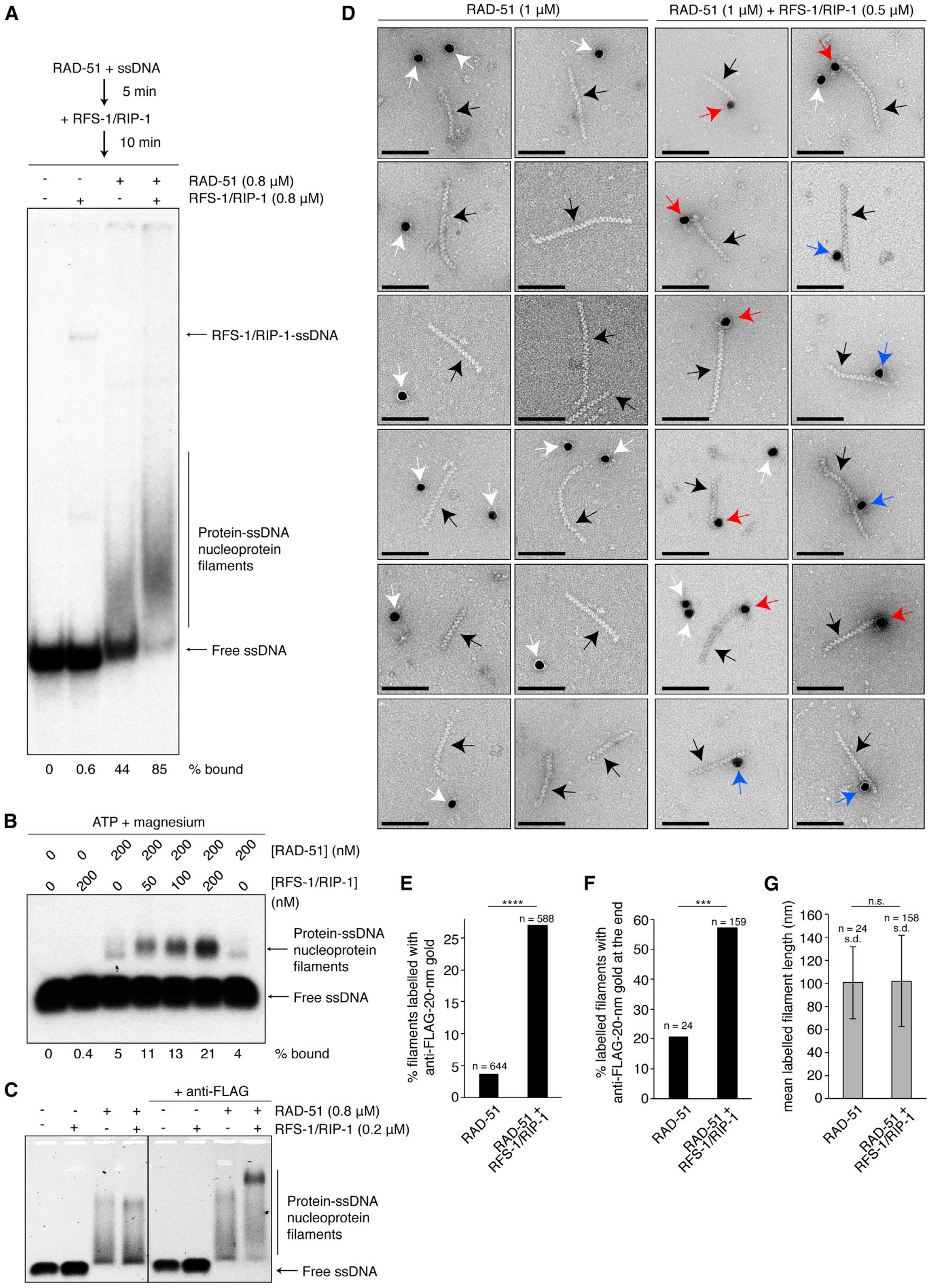

Figure 3. RFS-1/RIP-1 Binds and Modulates the Properties of RAD-51-ssDNA FILAMENTS

(A) Protein-DNA complexes formed by RAD-51 and RFS-1/RIP-1 on 60-mer ssDNA with ATP according to the mixing scheme indicated resolved by native PAGE.

(B) Proteins were pre-incubated before addition of 60-mer ssDNA, and then protein-DNA complexes were crosslinked and resolved in agarose gels.

(legend continued on next page)

(Figure 3G), suggesting RFS-1/RIP-1 does not modulate filament extension or disassembly. These data suggest that RFS-1/RIP-1 physically associates with RAD-51-ssDNA filaments, similar to the budding yeast Rad55-Rad57 and Shu complexes (Liu et al., 2011a; Sasanuma et al., 2013).

Stopped-Flow Measurements Reveal Rapid RAD-51-ssDNA Filament Formation in Real Time

To examine the functional impact of RFS-1/RIP-1 on RAD-51-ssDNA filaments, we employed a stopped-flow system to monitor protein-ssDNA complex dynamics in real time by rapidly mixing different combinations of RAD-51, RFS-1/RIP-1, and a 5′-Cy3 fluorescently labeled $(dT)_{43}$ oligonucleotide (Cy3-43-mer). Equal volumes of two solutions of interest were injected into a mixing chamber, and all concentrations quoted herein represent the value in the final reaction mixture. By monitoring changes in Cy3 fluorescence over time, we could quantitatively assess changes in the biophysical properties of the fluorophore as a proxy for changes in the nature of the protein-DNA association (Antony et al., 2009). Although fluorescence was stable in the absence of protein, rapid mixing of RAD-51 with the labeled DNA in the presence of ATP resulted in an increase in fluorescence as a function of time (Figure 4A). Both the size and the rate of change in fluorescence, represented as Δ Cy3 fluorescence and half-time, respectively, increased as a function of RAD-51 concentration (Figures S4A, S4I, and S4J) and serves as a readout for RAD-51-ssDNA filament formation. In contrast, addition of RFS-1/RIP-1 alone did not change fluorescence of Cy3-43-mer (Figure 4B).

RFS-1/RIP-1 Changes the Biophysical Properties of Pre-formed RAD-51-ssDNA Filaments

Next, we examined the influence of RFS-1/RIP-1 on pre-formed RAD-51-ssDNA filaments using the stopped-flow system. Addition of RFS-1/RIP-1 caused a concentration-dependent decrease in fluorescence (Figures 4C, 4F, S4B, and S4C). The rate of fluorescence change also increased with RFS-1/RIP-1 concentration (half-time 12.40 $\pm$ 4.07 and 4.10 $\pm$ 1.87 s for 100 and 1,000 nM RFS-1/RIP-1, respectively; Figure S4B). This result demonstrates that RFS-1/RIP-1 is able to rapidly modulate pre-formed RAD-51-ssDNA filaments, arguing for a major activity of RFS-1/RIP-1 at a step downstream of RAD-51 loading onto ssDNA (Figures 3C and 3D).

We considered two interpretations for the reduction in fluorescence of RAD-51-ssDNA filaments induced by RFS-1/RIP-1: (1) filament disassembly or (2) filament remodeling. We do not favor the RAD-51-ssDNA filament disassembly model for the following reasons: (1) EMSA data show protein-DNA complex formation is increased, not reduced, in the presence of RFS-1/RIP-1 (Figures 3A, 3B, and S3A–S3E), (2) filaments do not change in length in EM after co-incubation with RFS-1/RIP-1 (Figures 3D–3G and S3F), (3) RFS-1/RIP-1 stimulates RAD-51 recombinase activity (Figures 2C and S2D), which is dependent on the RAD-51-ssDNA filament, and (4) RFS-1/RIP-1 promotes RAD-51 focus formation in vivo (Figure 1D). All these observations are in striking contrast to Srs2, which disassembles Rad51-ssDNA filaments, inhibits Rad51 recombinase activity, and antagonizes Rad51 focus formation in yeast (Burgess et al., 2009; Krejci et al., 2003). We therefore favor the second model, in which the reduction in fluorescence is attributed to a change in the biophysical properties (hereafter referred to as "remodeling") of the RAD-51-ssDNA filaments.

Given the dramatic differences in the rates of fluorescence change attributed to RAD-51 binding and filament remodeling (compare half-times in Figures S4A and S4B), we asked whether these distinct phenomena could be temporally separated by pre-incubating RAD-51 with different concentrations of RFS-1/RIP-1, followed by rapid mixing with Cy3-43-mer (Figure 4D). RAD-51 alone displayed a profile of fluorescence increase with similar kinetics to the corresponding experiment in Figure 4A (Figures S4A and S4D). Remarkably, in the presence of RFS-1/RIP-1, a biphasic profile of fluorescence as a function of time was observed, with fluorescence reaching a maximum before declining. We attribute the rapid initial increase in fluorescence, which is only weakly influenced by RFS-1/RIP-1, to RAD-51-ssDNA filament formation (Figures 4D, S4D, and S4E). In contrast, after this initial rapid phase, a clear RFS-1/RIP-1 concentration-dependent reduction in fluorescence intensity was observed (Figures S4D and S4F). The rate of the second phase was comparable to the effect of RFS-1/RIP-1 on pre-formed RAD-51-ssDNA filaments observed in Figure 4C (Figures S4B and S4D), suggesting this slower phase represents the same slower filament-remodeling phase. As a variant of this experiment, we omitted ATP from the syringe containing the proteins (Figure 4E) and observed a much slower increase in fluorescence (compare Figures 4A and 4E), suggesting that ATP binding to RAD-51 primes it for rapid filament assembly. Notably, however, inclusion of RFS-1/RIP-1 led to a concentration-dependent reduction of fluorescence increase (Figures 4E, 4G, S4G, and S4H).

Analysis of longer time courses revealed that the fluorescence of the remodeled filaments reached equilibrium in all experiments (Figures S4K–S4N). For the experiment in which filament formation and remodeling were temporally segregated, at all RFS-1/RIP-1 concentrations $\geq$100 nM (Figures S4D, S4F, S4M, and S4N), a similar end point of the remodeling phase

(C) Immuno-shift analysis of native protein-DNA complexes formed by RAD-51 and RFS-1/RIP-1 on 60-mer ssDNA, followed by incubation with anti-FLAG antibodies and resolution by agarose gel electrophoresis. Black line: cropped superfluous gel lanes.

(D) Anti-FLAG-20-nm immuno-gold EM analysis of RAD-51-ssDNA filaments (black arrows) incubated with RFS-1/RIP-1. Scale bar, 100 nm. White arrows: unbound gold; red arrows: gold bound to filament ends; blue arrows: gold bound to filament body.

(E) Anti-FLAG-20-nm gold binding is specifically enriched on filaments co-incubated with RFS-1/RIP-1 (Fisher's exact test; $p < 0.0001$).

(F) Anti-FLAG-20-nm gold is enriched at gold-bound filament ends in the presence of RFS-1/RIP-1 (Fisher's exact test; $p = 0.0009$).

(G) Gold-bound filament length is not significantly different in the presence or absence of RFS-1/RIP-1 (unpaired two-tailed t test; $p = 0.8405$). Error bars indicate SD.

See also Figure S3.

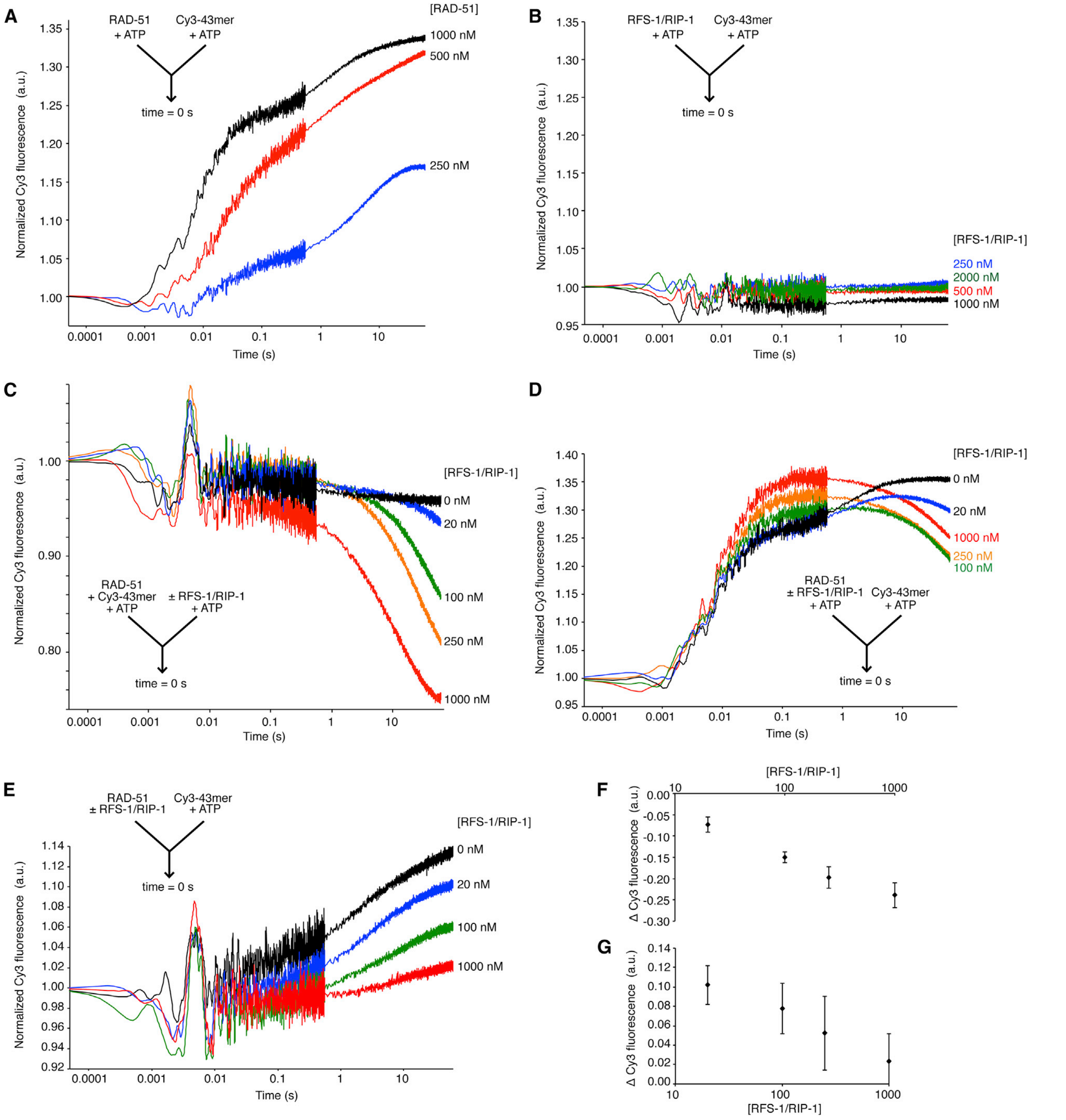

Figure 4. RFS-1/RIP-1 Remodels the RAD-51-ssDNA Filaments

(A–G) Analysis of average normalized Cy3-43-mer fluorescence (see the Experimental Procedures for details) plotted as a function of $\log_{10}$(time). The arrow (inset) indicates the components of the two syringes rapidly mixed at the 0 s time point in a stopped-flow instrument.

(A) Indicated concentrations of RAD-51 mixed with 15 nM Cy3-43-mer (both +ATP) (n = 8–9).

(B) Indicated concentrations of RFS-1/RIP-1 mixed with 15 nM Cy3-43-mer (both +ATP) (n = 5–6).

(C) RAD-51-ssDNA filaments pre-formed with 1 μM RAD-51 + 15 nM Cy3-43-mer for 10 min and then mixed with the indicated concentrations of RFS-1/RIP-1 (both +ATP) (n = 6–9).

(D) 1 μM RAD-51 pre-incubated with indicated concentrations of RFS-1/RIP-1 for 10 min and then mixed with 15 nM Cy3-43-mer (both +ATP) (n = 5–7).

(E) As in (D), except proteins were not pre-incubated with ATP (n = 6–9).

(F and G) Graphs of average Δ Cy3 fluorescence as a function of RFS-1/RIP-1 concentration for the data presented in (C) and (E), respectively. Error bars indicate SD. See also Figure S4.

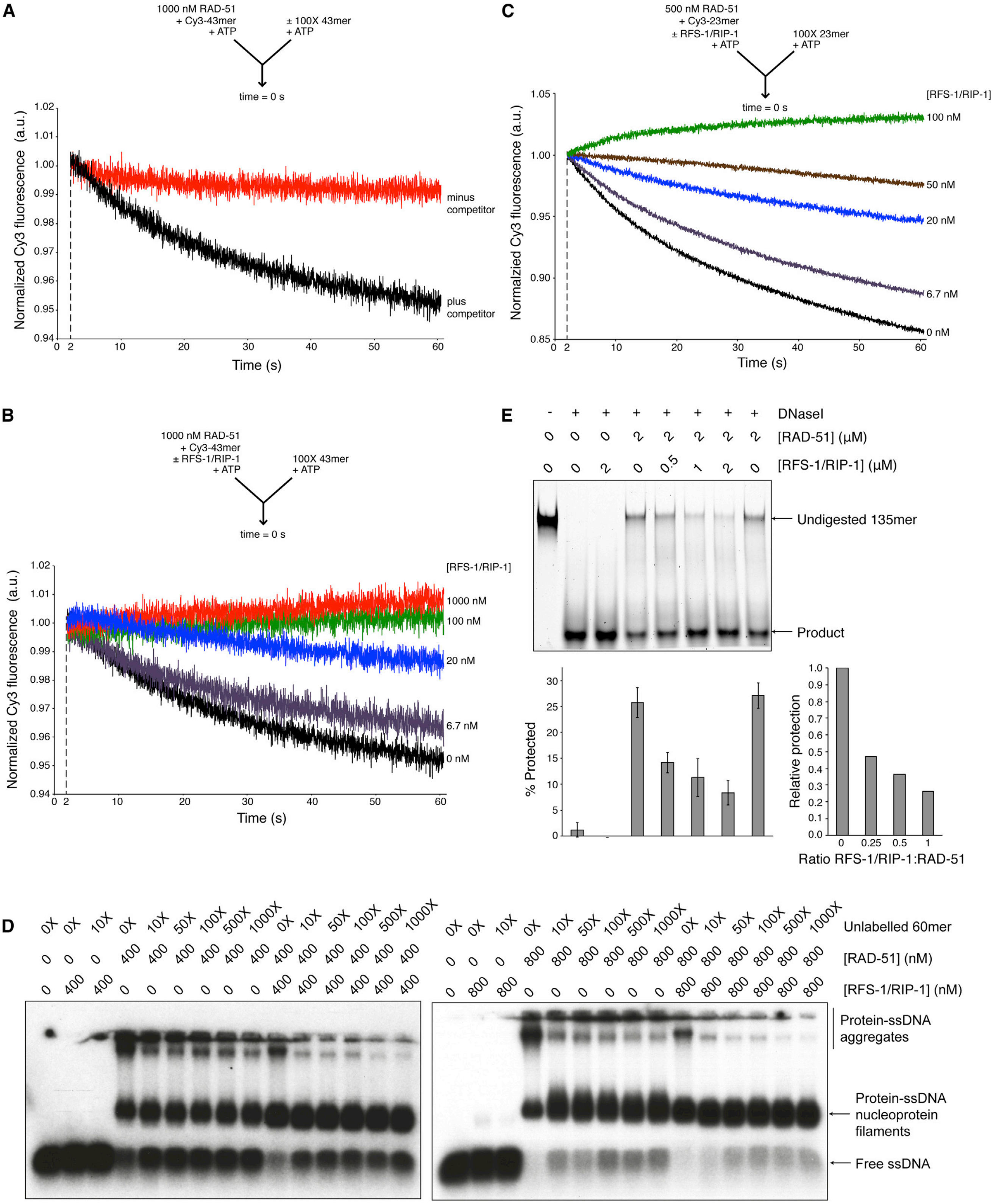

Figure 5. RFS-1/RIP-1 Stabilizes RAD-51-ssDNA Filaments in a Nuclease-Sensitive Conformation

(A–E) Analysis of average normalized Cy3-43-mer fluorescence (see the Experimental Procedures for details) plotted as a function of time. The arrow (inset) indicates the components of the two syringes rapidly mixed at the 0 s time point in a stopped-flow instrument in the presence of ATP.

(legend continued on next page)

was attained that was intermediate to naked DNA and unremodeled filaments, demonstrating sub-stoichiometric quantities of RFS-1/RIP-1 are sufficient to drive RAD-51 filament remodeling. We also verified that RFS-1/RIP-1 stimulates D loop formation at the same RAD-51-to-ssDNA ratio as was used in the stopped-flow experiments (Figure S2E), confirming the relevance of these observations to the mechanism of RAD-51 recombinase stimulation by RFS-1/RIP-1.

RFS-1/RIP-1 Stabilizes RAD-51-ssDNA Filaments

To determine whether RAD-51-ssDNA filament stability is influenced by RFS-1/RIP-1, we challenged RAD-51-ssDNA filaments pre-formed on Cy3-43-mer in the presence or absence of RFS-1/RIP-1 with a 100-fold excess of unlabeled, matched competitor 43-mer in the stopped-flow system. In the absence of RFS-1/RIP-1, fluorescence declined as a function of time, consistent with the ability of unlabeled competitor DNA to bind RAD-51 dissociated from the labeled DNA (Figure 5A). In contrast, addition of as little as 300-fold less RFS-1/RIP-1 (3.3 nM) reduced the extent of the change in fluorescence in a concentration-dependent manner (Figures 5B, S5A, and S5B), suggesting that RFS-1/RIP-1 stabilizes RAD-51 binding to ssDNA at sub-stoichiometric ratios. We verified this observation using half the amount of RAD-51 pre-bound to an oligonucleotide of approximately half the length (23-mer) (Figures 5C, S5C, and S5D). We also monitored filament stability directly by EMSA upon titration of unlabeled scavenger DNA. The levels of labeled free DNA liberated by RAD-51 turnover from the filament were reduced in the presence of RFS-1/RIP-1 (Figure 5D), verifying that the remodeled filaments are more stable. Interestingly, filaments formed in the presence of an excess of RAD-51 tended to aggregate and could not be resolved in agarose gels after crosslinking. This aggregation was reduced by RFS-1/RIP-1, while retaining a large population of resolved filaments (Figure 5D). Together, these data argue that RFS-1/RIP-1 remodels RAD-51-ssDNA filaments to a form in which RAD-51 is more stably associated with ssDNA and the filaments are less prone to aggregation after crosslinking, suggesting filament remodeling may reflect a conformational change in the pre-synaptic complex.

RFS-1/RIP-1 Sensitizes ssDNA within RAD-51 Filaments to DNaseI Digestion

To directly probe for potential structural changes associated with remodeling of RAD-51-ssDNA filaments by RFS-1/RIP-1, we attempted to perform three-dimensional reconstructions from electron micrographs (Figure S3F). Averaged power spectra revealed that the pitch of the filament helix (~90 Å) was unaltered with and without RFS-1/RIP-1 (Figure S3F). However, under the conditions used, the filaments were highly heterogeneous due to binding of RFS-1/RIP-1, precluding the generation of high-resolution reconstructions of filament architecture.

We reasoned that a change in the structural properties of the filaments could alter the accessibility of the ssDNA to degradation by nucleases (Zaitsev and Kowalczykowski, 1999). Using a nuclease protection assay to monitor the sensitivity of pre-formed protein-DNA complexes to DNaseI, we observed that the addition of sub-stoichiometric quantities of RFS-1/RIP-1 to RAD-51 caused a dramatic and unexpected increase in the DNaseI sensitivity of the protein-DNA complexes (Figure 5E). We verified these observations under similar buffer conditions to D loop formation and on the 60-mer oligonucleotide used in EMSA (Figures S5E–S5G), demonstrating that the effect is robust, independent of oligonucleotide properties, and relevant to the stimulation of RAD-51 recombinase activity.

Since RAD-51-ssDNA filaments are more stable in the presence of RFS-1/RIP-1 (Figure 5B–5D), de-protection is unlikely due to increased treadmilling by RAD-51 on ssDNA. To verify this, we performed the nuclease protection assay under conditions in which RAD-51 turnover from ssDNA is impaired. Since RAD-51-ssDNA filament disassembly is dependent on ATP hydrolysis by RAD-51 within the filament, we performed assays in the presence of a peptide of *C. elegans* BRC-2 that stabilizes RAD-51-ssDNA filaments by inhibiting RAD-51 ATP hydrolysis (Figure S5H) (Petalcorin et al., 2007). RFS-1/RIP-1 still conferred DNaseI sensitization, confirming this assay reflects a change to a more "open" filament conformation, rather than filament disassembly.

Walker Box Mutations in RFS-1 Compromise Mediator Activity and Filament Remodeling

The Walker motifs of Rad51 paralogs are important for resistance to DNA-damaging agents in vivo (French et al., 2003; Gruver et al., 2005; Wiese et al., 2006; Yamada et al., 2004), but the biochemical function of these motifs is unclear. To examine the functional importance of the Walker motifs in RFS-1 for the activity of RFS-1/RIP-1, we purified single-point mutants in conserved residues in either the Walker A (K56A) or the Walker B (E138A) box of RFS-1 (Figure 6A). Both mutants were defective for stimulation of D loop formation by RAD-51 (Figure 6B). At the same time, neither mutant was able to remodel the RAD-51-ssDNA filaments to a nuclease-sensitive conformation (Figures

(A) RAD-51-ssDNA filaments pre-formed with 1 μM RAD-51 + 15 nM Cy3-43-mer for 10 min and then mixed with (black) or without (red) 100-fold excess unlabeled 43-mer (n = 4–6).
(B) RAD-51-ssDNA filaments pre-formed with 1 μM RAD-51 + 15 nM Cy3-43-mer and indicated concentrations of RFS-1/RIP-1 for 10 min and then mixed with 100-fold excess unlabeled 43-mer (n = 4–6).
(C) RAD-51-ssDNA filaments pre-formed with 500 nM RAD-51 + 15 nM Cy3-23-mer and indicated concentrations of RFS-1/RIP-1 for 10 min and then mixed with 100-fold excess unlabeled 23-mer (n = 5–8).
(D) Proteins were pre-incubated before addition of radiolabeled 60-mer ssDNA for 10 min and then challenged with the indicated molar excess of unlabeled 60-mer for a further 10 min. Protein-DNA complexes were crosslinked and resolved in agarose gels.
(E) DNaseI protection assay on protein-DNA complexes formed by RAD-51 and RFS-1/RIP-1 on ssDNA with ATP. Error bars indicate SD (n = 4). The chart indicates the extent of protection by comparison with EMSA data in Figure S3B relative to RAD-51 alone.
See also Figure S5.

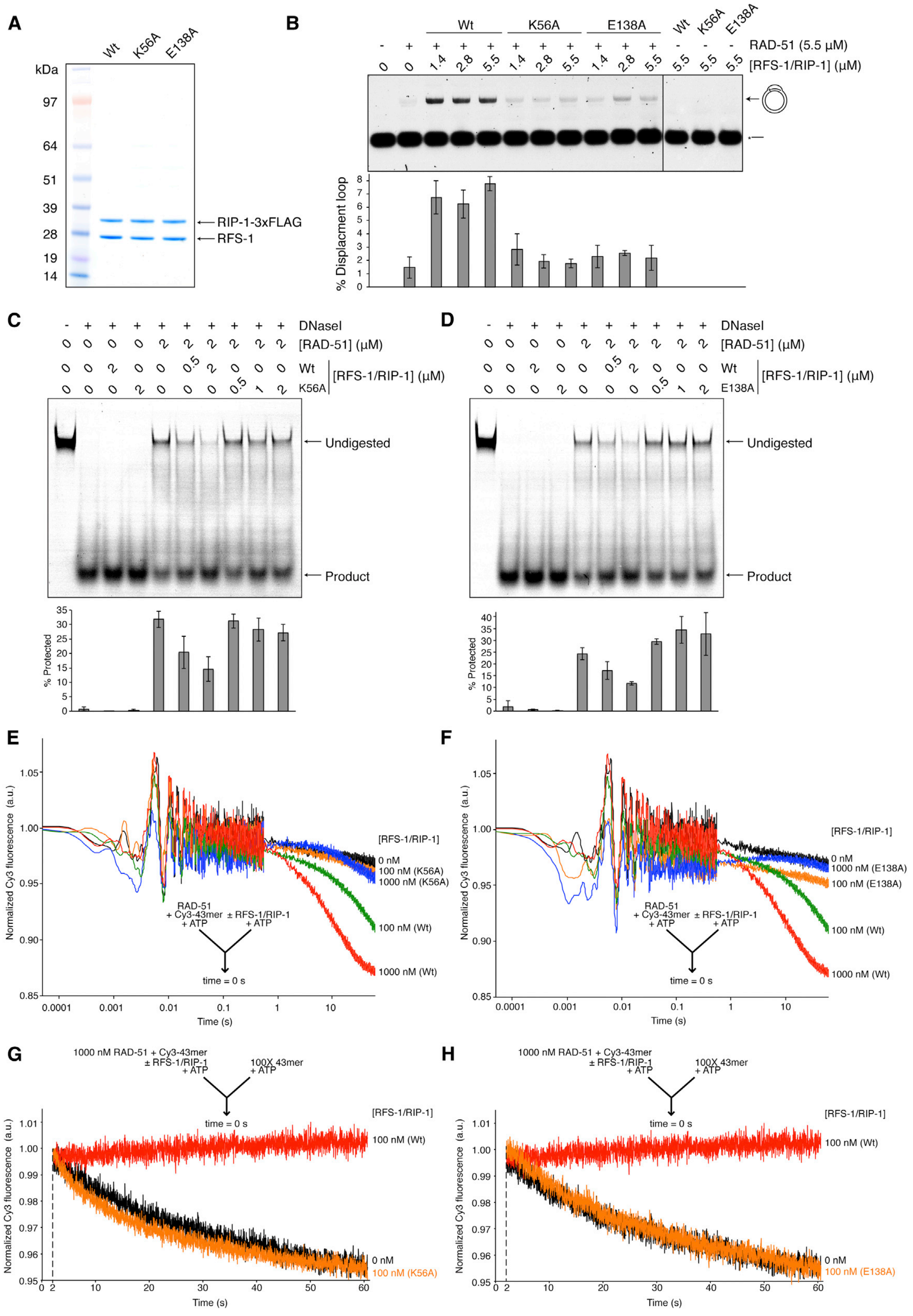

(legend on next page)

6C and 6D) nor reduce the fluorescence of pre-formed RAD-51-ssDNA filaments on Cy3-labeled DNA in stopped-flow experiments (Figures 6E, 6F, and S6C). Furthermore, the filaments formed in the presence of these mutants were not stabilized against scavenger DNA (Figure 6G, 6H, and S6D), in contrast to the wild-type complex.

Although both mutant complexes bound to RAD-51-ssDNA filaments similarly to wild-type RFS-1/RIP-1 in EMSA (Figures S6A and S6B), they exhibited increased ssDNA affinity (Figure S6B) and formed abnormal protein-ssDNA complexes with RAD-51 after crosslinking (Figure S6A). We also assessed the effect of mutating these residues on the RFS-1/RIP-1/RAD-51 interaction network in yeast two-hybrid and discovered that the RFS-1/RAD-51 interaction was impaired (Figure S6E). Co-expression of RIP-1 in the RFS-1/RAD-51 yeast two-hybrid strains impaired the RFS-1/RAD-51 interaction, suggesting RAD-51 and RIP-1 compete for a common binding surface on RAD-51 in the absence of DNA, but this inhibition was defective in the context of the RFS-1 Walker box mutations (Figure S6F). Together, these observations suggest K56A and E138A mutant complexes interact abnormally with ssDNA, RAD-51, and RAD-51-ssDNA filaments. Crucially, these findings reveal that filament remodeling is intrinsic to the RFS-1/RIP-1 complex, required for its mediator activity, and dependent on the intact Walker boxes of RFS-1.

RFS-1/RIP-1 Converts RAD-51-ssDNA Filaments to a More Flexible Conformation

To probe the nature of the conformational change induced in the pre-synaptic complex by RFS-1/RIP-1, we performed single-molecule FRET (smFRET) experiments. Filament properties were monitored by measuring FRET efficiency between a Cy3 donor and a Cy5 acceptor fluorophore, separated by seven nucleotides in surface-immobilized ssDNA constructs (Figure 7A). RAD-51 binding to ssDNA results in a dramatic decrease from 0.92 ± 0.01 (naked DNA; Figure 7A) to 0.47 ± 0.01 (DNA + RAD-51; Figure 7B) in mean FRET (x_0), due to both ssDNA stretching within the filament and a reduction in molecular flexibility relative to naked ssDNA. Co-incubation of RAD-51 with RFS-1/RIP-1 induces a striking increase to 0.64 ± 0.01 (Figures 7C and 7F) in mean FRET, relative to RAD-51 alone, as well as a broadening of the FRET distribution, indicated by the distribution width (σ), from σ = 0.20 ± 0.01 (DNA + RAD-51; Figure 7B) to σ = 0.25 ± 0.01 (DNA + RAD-51 + RFS-1/RIP-1; Figure 7C). In contrast, the majority of the DNA molecules bound by RFS-1/RIP-1 yield a mean FRET of 0.87 ± 0.01, similar to naked DNA (Figure S7A). These results were verified by binning the average FRET value of each trajectory independently (Figures S7D–S7G), instead of time binning each trajectory (Figures 7A–7C and S7A). Given that the filament helical pitch is equivalent in the presence and absence of RFS-1/RIP-1 (Figure S3F), this increase in FRET is unlikely due to filament compression. Instead, these results suggest that in the presence of RFS-1/RIP-1 the filaments adopt a substantially more flexible and less rigid conformation.

We also tested the effect of the K56A and E138A mutants of RFS-1 on filament flexibility by smFRET (Figures 7D, 7E, S7H, and S7I). Similar to wild-type RFS-1/RIP-1, neither mutant complex alone significantly altered FRET relative to naked DNA (Figures S3B and S3C). However, in the presence of RAD-51, we observed a bimodal FRET distribution of the molecules, which is not observed with wild-type RFS-1/RIP-1. The high FRET populations (mean FRET 0.74 ± 0.01 and 0.62 ± 0.01 for K56A and E138A, respectively) were similar to those observed for RAD-51 filaments co-incubated with wild-type RFS-1/RIP-1 (Figures 7F–7H), suggesting some filaments become flexible in the presence of the mutant RFS-1/RIP-1 complexes. In contrast, the low FRET populations (mean FRET 0.25 ± 0.01 and 0.22 ± 0.01 for K56A and E138A, respectively) exhibit lower mean FRET values than that observed for RAD-51 alone (Figures 7G and 7H), which may represent a more rigid intermediate state in the remodeling process, not observed in the presence of the RFS-1/RIP-1 mutant complexes alone (Figures S7B and S7C). These results establish that RFS-1/RIP-1 K56A and E138A mutant complexes are compromised for inducing or maintaining the filament in a flexible high FRET state, consistent with their defects in filament remodeling and mediator activity in ensemble experiments (Figure 6).

DISCUSSION

Rad51 Paralogs Remodel Rad51 Filaments to a Conformation More Proficient to Strand Exchange

In this study, we identify a biochemically tractable Rad51 paralog complex, RFS-1/RIP-1, which binds to and strongly stimulates the recombinase activity of RAD-51. Our biochemical and biophysical analysis reveals that the stimulatory activity of this Rad51 paralog complex on HR is due to its ability to induce a conformational change in the pre-synaptic filament. In contrast to BRCA2, RFS-1/RIP-1 does not primarily act to nucleate or extend RAD-51-ssDNA filaments. Instead, RFS-1/RIP-1 structurally remodels the pre-synaptic filament to a more "open," flexible, and stable conformation. Importantly, RAD-51-ssDNA filament remodeling is an intrinsic property of RFS-1/RIP-1 that is dependent on the Walker boxes of RFS-1. Since the

Figure 6. Walker Box Mutations in RFS-1 Prevent Filament Remodeling and Stimulation of Strand Exchange

(A) Purification of recombinant RFS-1(K56A)/RIP-1 and RFS-1(E138A)/RIP-1 from yeast cells by FLAG immunoprecipitation.

(B) RFS-1/RIP-1 mutants have impaired stimulation of D loop formation by RAD-51. Error bars indicate SD (n = 3).

(C and D) RFS-1(K56A)/RIP-1 (C) and RFS-1(E138A)/RIP-1 (D) mutants do not remodel RAD-51-ssDNA filaments to a DNaseI-sensitive conformation. Error bars indicate SD (n = 3).

(E and F) RFS-1(K56A)/RIP-1 (E) and RFS-1(E138A)/RIP-1 (F) mutants do not reduce the fluorescence of RAD-51-ssDNA filaments on Cy3-43-mer ssDNA in the same stopped-flow experimental setup as in Figure 4C (n = 6–8). For clarity, traces for wild-type (Wt) and RAD-51 alone are duplicated in (E) and (F).

(G and H) RFS-1(K56A)/RIP-1 (G) and RFS-1(E138A)/RIP-1 (H) mutants do not remodel RAD-51-ssDNA filaments to a stable conformation in the same stopped-flow experimental setup as in Figure 5B (n = 5–8). For clarity, traces for Wt and RAD-51 alone are duplicated in (G) and (H).

See also Figure S6.

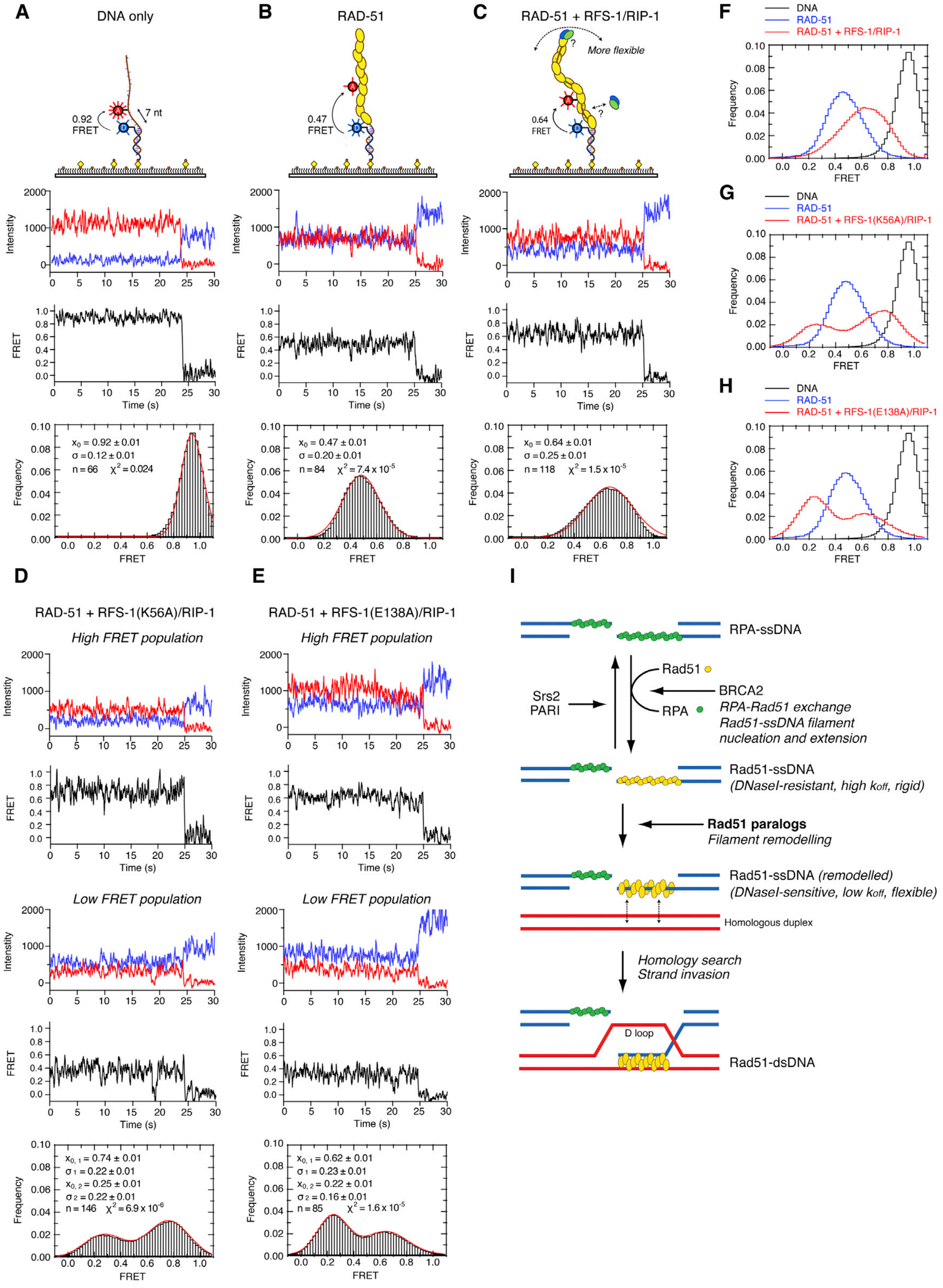

Figure 7. Remodeled RAD-51-ssDNA Filaments Are More Flexible

(A–E) smFRET analysis of (A) DNA alone, (B) DNA + RAD-51, (C) DNA + RAD-51 + RFS-1/RIP-1 (Wt), (D) DNA + RAD-51 + RFS-1(K56A)/RIP-1, and (E) DNA + RAD-51 + RFS-1(E138A)/RIP-1. Top to bottom: cartoon schematic of the smFRET experiment indicating FRET between Cy3 and Cy5 (7-nt separation) attached to biotinylated DNA constructs immobilized on a streptavidin-coated biotin-PEG surface; donor (blue) and acceptor (red) intensity trajectories are anti-correlated until single-step photobleaching of the acceptor; FRET trajectories (black) between Cy3 and Cy5 exhibit a sharp drop to zero FRET when the acceptor

(legend continued on next page)

Walker box mutants in RFS-1 are also deficient for stimulation of RAD-51 strand exchange activity, we propose that filament remodeling is a crucial molecular switch through which the Rad51 paralogs stimulate HR. Thus, our study provides mechanistic insights into the functional importance of the Walker boxes in Rad51 paralogs in resisting DNA damage in cells.

Biological Importance of Rad51 Filament Remodeling to HR

rfs-1 and *rip-1* mutant animals are defective for RAD-51 focus formation at stalled replication forks, and we propose this likely reflects a failure to remodel and stabilize RAD-51-ssDNA filaments. Although RAD-51 would be loaded onto chromatin normally in the absence of *rfs-1* or *rip-1*, its turnover would be more rapid, preventing detection of RAD-51 foci. Since Rad51 focus formation after damage is also defective in Rad51 paralog mutants from other eukaryotic organisms (Gasior et al., 1998), the filament remodeling function of RFS-1/RIP-1 to a stable conformation is likely to be conserved. This result is also consistent with the suppression of the DNA damage sensitivity and HR defects of yeast *rad55Δ* and *rad57Δ* mutants by a mutant form of Rad51 (I345T) that binds DNA more stably (Fortin and Symington, 2002).

A requirement for Rad51 paralogs in preventing filament disruption by Srs2 at DSBs (Liu et al., 2011a) is likely dispensable in nematodes as they lack a Srs2 ortholog. Although *rfs-1* and *rip-1* animals are sensitive to both IR and nitrogen mustard, but only defective in RAD-51 focus formation in response to the latter, this likely reflects the additional function for RFS-1/RIP-1 in late stages of meiotic DSB repair (Ward et al., 2010). Furthermore, the sensitivity of *rfs-1* animals to IR is relatively weak compared to deficiency in the core HR component BRC-1, whereas sensitivity of *rfs-1* and *brc-1* animals to cross-linking agents is virtually indistinguishable, in support of the greater specificity of RFS-1/RIP-1 toward stalled replication fork substrates (Ward et al., 2007).

Implications of Rad51 Filament Remodeling for the Homology Search

Structural remodeling of RAD-51-ssDNA filaments by RFS-1/RIP-1 to an "open," flexible, and stabilized conformation also has implications for the efficiency of the homology search. The structural transitions undergone by Rad51 filaments during homology sampling, template unwinding, strand exchange, and conversion of Rad51-ssDNA to Rad51-dsDNA filaments are extremely challenging to study due to their dynamic nature. Atomic resolution models of the RecA-ssDNA filament show that each nucleotide triplet associated with each RecA protomer adopts a local B-DNA conformation, but the DNA is stretched and underwound from one triplet to the next, resulting in a global underwound DNA conformation. This stretching may facilitate disruption of base pairing and stacking in the template duplex upon binding by the filament (Chen et al., 2008; Danilowicz et al., 2014). The DNaseI sensitivity of the remodeled filament indicates that the ssDNA is more accessible, and it is therefore possible that the remodeling induced by Rad51 paralogs exposes the ssDNA to facilitate homology probing after filament binding to and disruption of the donor duplex. A recent study demonstrated that Rad51 only stably captures template dsDNA harboring at least eight nucleotides of homology, reducing search complexity, and argued that physical discontinuities or gaps within the pre-synaptic complex could further limit search complexity by segregating the Rad51-ssDNA filament into non-overlapping functional search units (Qi et al., 2015). The binding of RFS-1/RIP-1 to or within the pre-synaptic filament could define such search unit boundaries. Alternatively, it is possible that the DNaseI sensitivity of the remodeled filament arises due to flexing of the protomers transiently, exposing naked ssDNA, or due to limited turnover of individual protomers within the filament (without complete filament disassembly), which could also introduce pre-synaptic complex discontinuities to facilitate homology searching.

It has also been known for many years that homology searching by RecA proceeds primarily by a processive 3D search process, facilitated by transient contacts with heterologous dsDNA to enhance the probability of locating homologous sequences (Forget and Kowalczykowski, 2012; Gonda and Radding, 1983, 1986; Honigberg et al., 1986; Tsang et al., 1985). The increased flexibility of the remodeled filament induced by RFS-1/RIP-1 in 3D space could aid such a 3D homology search mechanism. The increased stability of the remodeled Rad51 filament may also increase the lifetime of the homology search and provide more opportunities to locate the correct dsDNA template. Hence, all three altered properties of the remodeled filament are predicted to facilitate homology search during HR, and remodeling could therefore have a very important function in vivo in locating the correct dsDNA template.

Conclusions

Our observations, together with previously reported findings, suggest that Rad51 paralogs perform two distinct functions to promote HR: they protect Rad51-ssDNA filaments against disruption by antirecombinases (Liu et al., 2011a) and directly stimulate the intrinsic recombinase activity of Rad51 by remodeling pre-synaptic filaments to an active, "open," flexible, and stable conformation primed for homology search and strand invasion. Importantly, the mechanism we have discovered for the Rad51 paralogs in stimulating HR is distinct from that proposed for other positive regulators of HR, which are epistatic to Rad51 paralogs, including BRCA2 and Rad54 (Chun et al., 2013; Jensen et al., 2010, 2013; Liu et al., 2010; Solinger et al., 2002; Thorslund et al., 2010). We therefore propose a model (Figure 7I) for HR, in

photobleaches; histogram of FRET values collected from all molecules with a Gaussian fit are shown in red, where x_0 is the mean FRET, σ is the distribution width, and χ^2 quantifies the quality of the fit.

(F–H) Superimposed histograms from (A)–(E) showing the differences between the populations. Differences in the mean FRET are statistically significant (Student's t test; $p < 0.0005$).

(I) Model for the proposed function of Rad51 paralogs in Rad51 filament remodeling within the HR mechanism.

See also Figure S7.

which Rad51-ssDNA filaments are first nucleated by BRCA2, displacing RPA from ssDNA. Rad51 paralogs subsequently switch the filament to a more ‘‘open’’ and flexible structure, which is also less prone to disassembly. The altered properties of this remodeled pre-synaptic filament likely facilitate homology probing of the template and strand invasion to stimulate HR.

EXPERIMENTAL PROCEDURES

RFS-1/RIP-1 complex was co-expressed in yeast cells and purified by FLAG immunoprecipitation. RAD-51 was purified using the pET-SUMO system and the SUMO tag cleaved with Ulp1 SUMO protease to yield native protein followed by MonoQ ion-exchange chromatography. For EMSA, protein-DNA complexes were assembled on ^{32}P-labeled 60-mer ssDNA in the presence of ATP (10 min) and then resolved by native PAGE or crosslinked with 0.25% glutaraldehyde and resolved in agarose gels. In immuno-shift experiments, protein-DNA complexes assembled on fluorescently labeled 60-mer ssDNA were incubated with anti-FLAG antibody (Sigma F3165; 5 min) and resolved in agarose gels. Immuno-gold EM was performed by incubating RAD-51, RFS-1/RIP-1, and linearized PhiX ssDNA (10 min) and then incubating with anti-FLAG antibody conjugated to 20-nm gold particles (30 min), staining with uranyl acetate (2 min), and imaging. For nuclease protection assays, protein-DNA complexes were assembled on fluorescently labeled 135-mer ssDNA (10 min) before challenging with DNaseI (20 min), deproteinizing, and resolving DNA products by PAGE. D loop formation was performed by pre-incubating proteins and fluorescently labeled 90-mer ssDNA (15 min) before addition of pBluescript plasmid DNA (15 min), after which time reactions were deproteinized and resolved in agarose gels. Stopped-flow experiments were performed by rapidly mixing equal volumes of the indicated components and monitoring Cy3 fluorescence for 1 min using the following measurement protocol: (1) every 0.00005 s from 0–0.05 s, (2) every 0.0005 s from 0.05–0.56 s, and (3) every 0.02 s from 0.56–60.54 s. Raw data sets were normalized as follows: for binding and remodeling experiments (Figures 4, 6E, and 6F), data sets were normalized to the same starting value for Cy3 fluorescence, and for competition experiments (Figures 5A–5C, 6G, and 6H), data sets were normalized to the same value for Cy3 fluorescence at the 2.01998 s time point and truncated before this. Yeast two-hybrid and nematode genetic analysis were performed as previously described (Boulton et al., 2002; Ward et al., 2007, 2010). Full materials and methods, including details of stopped-flow data analysis and smFRET, are available in the Supplemental Experimental Procedures.

SUPPLEMENTAL INFORMATION

Supplemental Information includes Supplemental Experimental Procedures and seven figures and can be found with this article online at http://dx.doi.org/10.1016/j.cell.2015.06.015.

ACKNOWLEDGMENTS

We thank A. Alidoust and N. Patel for yeast fermentation and J. Frigola for assistance with protein expression in yeast. We are grateful to members of the S.J.B. and L.K. laboratories and M. Petalcorin, M. McIlwraith, S. West, S. Kowalczykowski, W. Dietrich-Heyer, and J. Svejstrup for comments and discussion throughout the project. This work was supported by a Cancer Research UK PhD studentship (to M.R.G.T.). The S.J.B. laboratory is funded by Cancer Research UK and an European Research Council advanced investigator grant (RecMitMei). S.J.B. is a Royal Society Wolfson Research Merit Award holder. The L.K. laboratory is supported by the Czech Science Foundation (GA13–26629S and GAP207/12/2323), the European Regional Development Fund - Project FNUSA-ICRC (no. CZ.1.05/1.1.00/02.0123), and project ICRC-ERA-HumanBridge (no. 316345), which is funded by the European Commission. The D.R. laboratory is funded by the Clinical Sciences Center of the Medical Research Council (RCUK MC-A658-5TY10) and a startup grant from the Imperial College London.

Received: January 8, 2015
Revised: March 27, 2015
Accepted: June 1, 2015
Published: July 16, 2015

REFERENCES

Antony, E., Tomko, E.J., Xiao, Q., Krejci, L., Lohman, T.M., and Ellenberger, T. (2009). Srs2 disassembles Rad51 filaments by a protein-protein interaction triggering ATP turnover and dissociation of Rad51 from DNA. Mol. Cell *35*, 105–115.

Boulton, S.J., Gartner, A., Reboul, J., Vaglio, P., Dyson, N., Hill, D.E., and Vidal, M. (2002). Combined functional genomic maps of the C. elegans DNA damage response. Science *295*, 127–131.

Burgess, R.C., Lisby, M., Altmannova, V., Krejci, L., Sung, P., and Rothstein, R. (2009). Localization of recombination proteins and Srs2 reveals anti-recombinase function in vivo. J. Cell Biol. *185*, 969–981.

Chapman, J.R., Taylor, M.R.G., and Boulton, S.J. (2012). Playing the end game: DNA double-strand break repair pathway choice. Mol. Cell *47*, 497–510.

Chen, Z., Yang, H., and Pavletich, N.P. (2008). Mechanism of homologous recombination from the RecA-ssDNA/dsDNA structures. Nature *453*, 489–494.

Chun, J., Buechelmaier, E.S., and Powell, S.N. (2013). Rad51 paralog complexes BCDX2 and CX3 act at different stages in the BRCA1-BRCA2-dependent homologous recombination pathway. Mol. Cell. Biol. *33*, 387–395.

Danilowicz, C., Peacock-Villada, A., Vlassakis, J., Facon, A., Feinstein, E., Kleckner, N., and Prentiss, M. (2014). The differential extension in dsDNA bound to Rad51 filaments may play important roles in homology recognition and strand exchange. Nucleic Acids Res. *42*, 526–533.

Forget, A.L., and Kowalczykowski, S.C. (2012). Single-molecule imaging of DNA pairing by RecA reveals a three-dimensional homology search. Nature *482*, 423–427.

Fortin, G.S., and Symington, L.S. (2002). Mutations in yeast Rad51 that partially bypass the requirement for Rad55 and Rad57 in DNA repair by increasing the stability of Rad51-DNA complexes. EMBO J. *21*, 3160–3170.

French, C.A., Masson, J.-Y., Griffin, C.S., O’Regan, P., West, S.C., and Thacker, J. (2002). Role of mammalian RAD51L2 (RAD51C) in recombination and genetic stability. J. Biol. Chem. *277*, 19322–19330.

French, C.A., Tambini, C.E., and Thacker, J. (2003). Identification of functional domains in the RAD51L2 (RAD51C) protein and its requirement for gene conversion. J. Biol. Chem. *278*, 45445–45450.

Gasior, S.L., Wong, A.K., Kora, Y., Shinohara, A., and Bishop, D.K. (1998). Rad52 associates with RPA and functions with rad55 and rad57 to assemble meiotic recombination complexes. Genes Dev. *12*, 2208–2221.

Godthelp, B.C., Wiegant, W.W., van Duijn-Goedhart, A., Schärer, O.D., van Buul, P.P.W., Kanaar, R., and Zdzienicka, M.Z. (2002). Mammalian Rad51C contributes to DNA cross-link resistance, sister chromatid cohesion and genomic stability. Nucleic Acids Res. *30*, 2172–2182.

Golmard, L., Caux-Moncoutier, V., Davy, G., Al Ageeli, E., Poirot, B., Tirapo, C., Michaux, D., Barbaroux, C., d’Enghien, C.D., Nicolas, A., et al. (2013). Germline mutation in the RAD51B gene confers predisposition to breast cancer. BMC Cancer *13*, 484.

Gonda, D.K., and Radding, C.M. (1983). By searching processively RecA protein pairs DNA molecules that share a limited stretch of homology. Cell *34*, 647–654.

Gonda, D.K., and Radding, C.M. (1986). The mechanism of the search for homology promoted by recA protein. Facilitated diffusion within nucleoprotein networks. J. Biol. Chem. *261*, 13087–13096.

Gruver, A.M., Miller, K.A., Rajesh, C., Smiraldo, P.G., Kaliyaperumal, S., Balder, R., Stiles, K.M., Albala, J.S., and Pittman, D.L. (2005). The ATPase motif in RAD51D is required for resistance to DNA interstrand crosslinking agents and interaction with RAD51C. Mutagenesis *20*, 433–440.

Hays, S.L., Firmenich, A.A., and Berg, P. (1995). Complex formation in yeast double-strand break repair: participation of Rad51, Rad52, Rad55, and Rad57 proteins. Proc. Natl. Acad. Sci. USA *92*, 6925–6929.

Honigberg, S.M., Rao, B.J., and Radding, C.M. (1986). Ability of RecA protein to promote a search for rare sequences in duplex DNA. Proc. Natl. Acad. Sci. USA *83*, 9586–9590.

Howlett, N.G., Taniguchi, T., Olson, S., Cox, B., Waisfisz, Q., De Die-Smulders, C., Persky, N., Grompe, M., Joenje, H., Pals, G., et al. (2002). Biallelic inactivation of BRCA2 in Fanconi anemia. Science *297*, 606–609.

Jensen, R.B., Carreira, A., and Kowalczykowski, S.C. (2010). Purified human BRCA2 stimulates RAD51-mediated recombination. Nature *467*, 678–683.

Jensen, R.B., Ozes, A., Kim, T., Estep, A., and Kowalczykowski, S.C. (2013). BRCA2 is epistatic to the RAD51 paralogs in response to DNA damage. DNA Repair (Amst.) *12*, 306–311.

Jeyasekharan, A.D., Liu, Y., Hattori, H., Pisupati, V., Jonsdottir, A.B., Rajendra, E., Lee, M., Sundaramoorthy, E., Schlachter, S., Kaminski, C.F., et al. (2013). A cancer-associated BRCA2 mutation reveals masked nuclear export signals controlling localization. Nat. Struct. Mol. Biol. *20*, 1191–1198.

Johnson, R.D., Liu, N., and Jasin, M. (1999). Mammalian XRCC2 promotes the repair of DNA double-strand breaks by homologous recombination. Nature *401*, 397–399.

Krejci, L., Van Komen, S., Li, Y., Villemain, J., Reddy, M.S., Klein, H., Ellenberger, T., and Sung, P. (2003). DNA helicase Srs2 disrupts the Rad51 presynaptic filament. Nature *423*, 305–309.

Lancaster, J.M., Wooster, R., Mangion, J., Phelan, C.M., Cochran, C., Gumbs, C., Seal, S., Barfoot, R., Collins, N., Bignell, G., et al. (1996). BRCA2 mutations in primary breast and ovarian cancers. Nat. Genet. *13*, 238–240.

Lin, Z., Kong, H., Nei, M., and Ma, H. (2006). Origins and evolution of the recA/RAD51 gene family: evidence for ancient gene duplication and endosymbiotic gene transfer. Proc. Natl. Acad. Sci. USA *103*, 10328–10333.

Liu, J., Doty, T., Gibson, B., and Heyer, W.-D. (2010). Human BRCA2 protein promotes RAD51 filament formation on RPA-covered single-stranded DNA. Nat. Struct. Mol. Biol. *17*, 1260–1262.

Liu, J., Renault, L., Veaute, X., Fabre, F., Stahlberg, H., and Heyer, W.-D. (2011a). Rad51 paralogues Rad55-Rad57 balance the antirecombinase Srs2 in Rad51 filament formation. Nature *479*, 245–248.

Liu, T., Wan, L., Wu, Y., Chen, J., and Huang, J. (2011b). hSWS1·SWSAP1 is an evolutionarily conserved complex required for efficient homologous recombination repair. J. Biol. Chem. *286*, 41758–41766.

Loveday, C., Turnbull, C., Ramsay, E., Hughes, D., Ruark, E., Frankum, J.R., Bowden, G., Kalmyrzaev, B., Warren-Perry, M., Snape, K., et al.; Breast Cancer Susceptibility Collaboration (UK) (2011). Germline mutations in RAD51D confer susceptibility to ovarian cancer. Nat. Genet. *43*, 879–882.

Martin, J.S., Winkelmann, N., Petalcorin, M.I.R., McIlwraith, M.J., and Boulton, S.J. (2005). RAD-51-dependent and -independent roles of a Caenorhabditis elegans BRCA2-related protein during DNA double-strand break repair. Mol. Cell. Biol. *25*, 3127–3139.

Martín, V., Chahwan, C., Gao, H., Blais, V., Wohlschlegel, J., Yates, J.R., 3rd, McGowan, C.H., and Russell, P. (2006). Sws1 is a conserved regulator of homologous recombination in eukaryotic cells. EMBO J. *25*, 2564–2574.

Masson, J.Y., Tarsounas, M.C., Stasiak, A.Z., Stasiak, A., Shah, R., McIlwraith, M.J., Benson, F.E., and West, S.C. (2001). Identification and purification of two distinct complexes containing the five RAD51 paralogs. Genes Dev. *15*, 3296–3307.

Meindl, A., Hellebrand, H., Wiek, C., Erven, V., Wappenschmidt, B., Niederacher, D., Freund, M., Lichtner, P., Hartmann, L., Schaal, H., et al. (2010). Germline mutations in breast and ovarian cancer pedigrees establish RAD51C as a human cancer susceptibility gene. Nat. Genet. *42*, 410–414.

Petalcorin, M.I.R., Sandall, J., Wigley, D.B., and Boulton, S.J. (2006). CeBRC-2 stimulates D-loop formation by RAD-51 and promotes DNA single-strand annealing. J. Mol. Biol. *361*, 231–242.

Petalcorin, M.I.R., Galkin, V.E., Yu, X., Egelman, E.H., and Boulton, S.J. (2007). Stabilization of RAD-51-DNA filaments via an interaction domain in Caenorhabditis elegans BRCA2. Proc. Natl. Acad. Sci. USA *104*, 8299–8304.

Pierce, A.J., Johnson, R.D., Thompson, L.H., and Jasin, M. (1999). XRCC3 promotes homology-directed repair of DNA damage in mammalian cells. Genes Dev. *13*, 2633–2638.

Qi, Z., Redding, S., Lee, J.Y., Gibb, B., Kwon, Y., Niu, H., Gaines, W.A., Sung, P., and Greene, E.C. (2015). DNA sequence alignment by microhomology sampling during homologous recombination. Cell *160*, 856–869.

Rahman, N., Seal, S., Thompson, D., Kelly, P., Renwick, A., Elliott, A., Reid, S., Spanova, K., Barfoot, R., Chagtai, T., et al.; Breast Cancer Susceptibility Collaboration (UK) (2007). PALB2, which encodes a BRCA2-interacting protein, is a breast cancer susceptibility gene. Nat. Genet. *39*, 165–167.

Rattray, A.J., and Symington, L.S. (1995). Multiple pathways for homologous recombination in Saccharomyces cerevisiae. Genetics *139*, 45–56.

Reid, S., Schindler, D., Hanenberg, H., Barker, K., Hanks, S., Kalb, R., Neveling, K., Kelly, P., Seal, S., Freund, M., et al. (2007). Biallelic mutations in PALB2 cause Fanconi anemia subtype FA-N and predispose to childhood cancer. Nat. Genet. *39*, 162–164.

San Filippo, J., Sung, P., and Klein, H. (2008). Mechanism of eukaryotic homologous recombination. Annu. Rev. Biochem. *77*, 229–257.

Sasanuma, H., Tawaramoto, M.S., Lao, J.P., Hosaka, H., Sanda, E., Suzuki, M., Yamashita, E., Hunter, N., Shinohara, M., Nakagawa, A., and Shinohara, A. (2013). A new protein complex promoting the assembly of Rad51 filaments. Nat. Commun. *4*, 1676.

Shor, E., Weinstein, J., and Rothstein, R. (2005). A genetic screen for top3 suppressors in Saccharomyces cerevisiae identifies SHU1, SHU2, PSY3 and CSM2: four genes involved in error-free DNA repair. Genetics *169*, 1275–1289.

Sigurdsson, S., Van Komen, S., Bussen, W., Schild, D., Albala, J.S., and Sung, P. (2001). Mediator function of the human Rad51B-Rad51C complex in Rad51/RPA-catalyzed DNA strand exchange. Genes Dev. *15*, 3308–3318.

Solinger, J.A., Kiianitsa, K., and Heyer, W.D. (2002). Rad54, a Swi2/Snf2-like recombinational repair protein, disassembles Rad51:dsDNA filaments. Mol. Cell *10*, 1175–1188.

Sung, P. (1997). Yeast Rad55 and Rad57 proteins form a heterodimer that functions with replication protein A to promote DNA strand exchange by Rad51 recombinase. Genes Dev. *11*, 1111–1121.

Takata, M., Sasaki, M.S., Sonoda, E., Fukushima, T., Morrison, C., Albala, J.S., Swagemakers, S.M., Kanaar, R., Thompson, L.H., and Takeda, S. (2000). The Rad51 paralog Rad51B promotes homologous recombinational repair. Mol. Cell. Biol. *20*, 6476–6482.

Takata, M., Sasaki, M.S., Tachiiri, S., Fukushima, T., Sonoda, E., Schild, D., Thompson, L.H., and Takeda, S. (2001). Chromosome instability and defective recombinational repair in knockout mutants of the five Rad51 paralogs. Mol. Cell. Biol. *21*, 2858–2866.

Tao, Y., Li, X., Liu, Y., Ruan, J., Qi, S., Niu, L., and Teng, M. (2012). Structural analysis of Shu proteins reveals a DNA binding role essential for resisting damage. J. Biol. Chem. *287*, 20231–20239.

Thorslund, T., McIlwraith, M.J., Compton, S.A., Lekomtsev, S., Petronczki, M., Griffith, J.D., and West, S.C. (2010). The breast cancer tumor suppressor BRCA2 promotes the specific targeting of RAD51 to single-stranded DNA. Nat. Struct. Mol. Biol. *17*, 1263–1265.

Tsang, S.S., Chow, S.A., and Radding, C.M. (1985). Networks of DNA and RecA protein are intermediates in homologous pairing. Biochemistry *24*, 3226–3232.

Vaz, F., Hanenberg, H., Schuster, B., Barker, K., Wiek, C., Erven, V., Neveling, K., Endt, D., Kesterton, I., Autore, F., et al. (2010). Mutation of the RAD51C gene in a Fanconi anemia-like disorder. Nat. Genet. *42*, 406–409.

Ward, J.D., Barber, L.J., Petalcorin, M.I., Yanowitz, J., and Boulton, S.J. (2007). Replication blocking lesions present a unique substrate for homologous recombination. EMBO J. *26*, 3384–3396.

Ward, J.D., Muzzini, D.M., Petalcorin, M.I.R., Martinez-Perez, E., Martin, J.S., Plevani, P., Cassata, G., Marini, F., and Boulton, S.J. (2010). Overlapping mechanisms promote postsynaptic RAD-51 filament disassembly during meiotic double-strand break repair. Mol. Cell *37*, 259–272.

Wiese, C., Hinz, J.M., Tebbs, R.S., Nham, P.B., Urbin, S.S., Collins, D.W., Thompson, L.H., and Schild, D. (2006). Disparate requirements for the Walker A and B ATPase motifs of human RAD51D in homologous recombination. Nucleic Acids Res. *34*, 2833–2843.

Wooster, R., Bignell, G., Lancaster, J., Swift, S., Seal, S., Mangion, J., Collins, N., Gregory, S., Gumbs, C., and Micklem, G. (1995). Identification of the breast cancer susceptibility gene BRCA2. Nature *378*, 789–792.

Xia, B., Dorsman, J.C., Ameziane, N., de Vries, Y., Rooimans, M.A., Sheng, Q., Pals, G., Errami, A., Gluckman, E., Llera, J., et al. (2007). Fanconi anemia is associated with a defect in the BRCA2 partner PALB2. Nat. Genet. *39*, 159–161.

Yamada, N.A., Hinz, J.M., Kopf, V.L., Segalle, K.D., and Thompson, L.H. (2004). XRCC3 ATPase activity is required for normal XRCC3-Rad51C complex dynamics and homologous recombination. J. Biol. Chem. *279*, 23250–23254.

Yonetani, Y., Hochegger, H., Sonoda, E., Shinya, S., Yoshikawa, H., Takeda, S., and Yamazoe, M. (2005). Differential and collaborative actions of Rad51 paralog proteins in cellular response to DNA damage. Nucleic Acids Res. *33*, 4544–4552.

Zaitsev, E.N., and Kowalczykowski, S.C. (1999). The simultaneous binding of two double-stranded DNA molecules by Escherichia coli RecA protein. J. Mol. Biol. *287*, 21–31.

Supplemental Figures

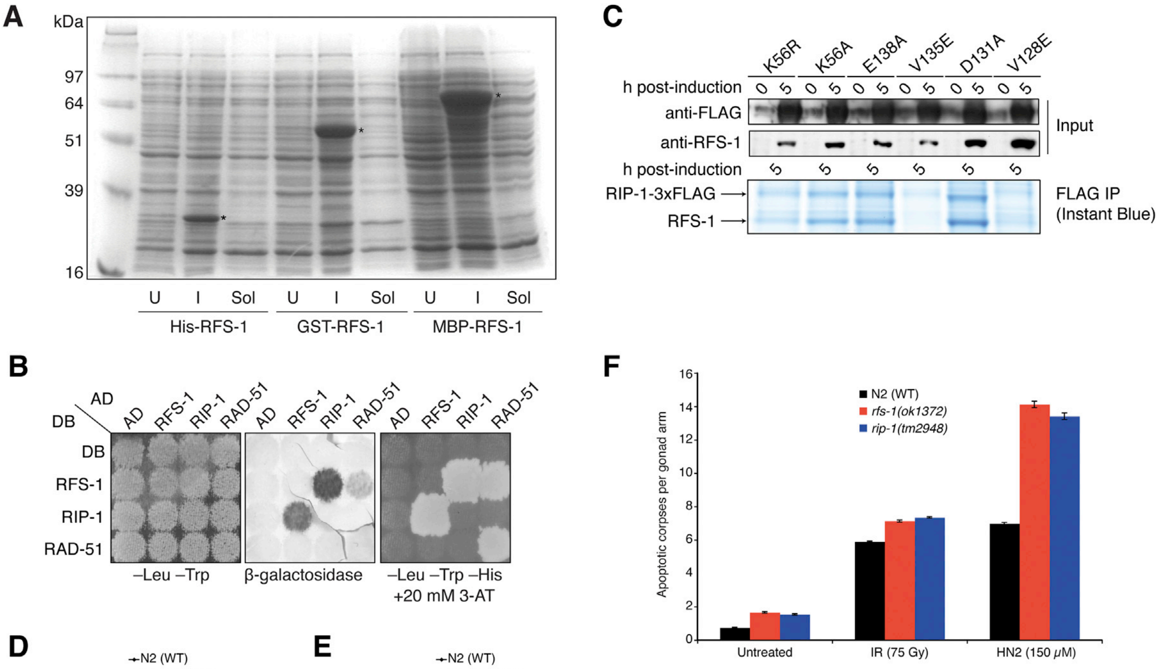

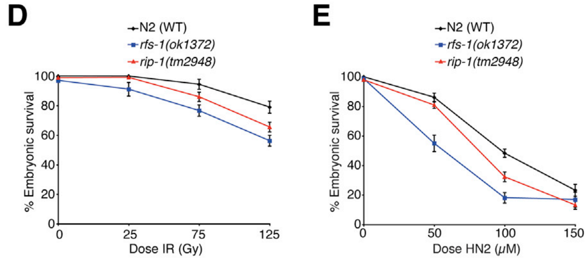

G

Genotype	Brood size (± S.D.)	% Embryonic lethal	% Males
N2	257.2 ± 20.3	0.63 (n = 471)	0 (n = 1286)
rfs-1(ok1372)	206.9 ± 73.1	0.13 (n = 2276)	2.20 (n = 2276)
rip-1(tm2948)	194.0 ± 40.4	1.48 (n = 1723)	1.00 (n = 2328)

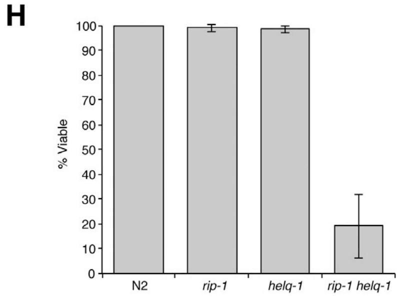

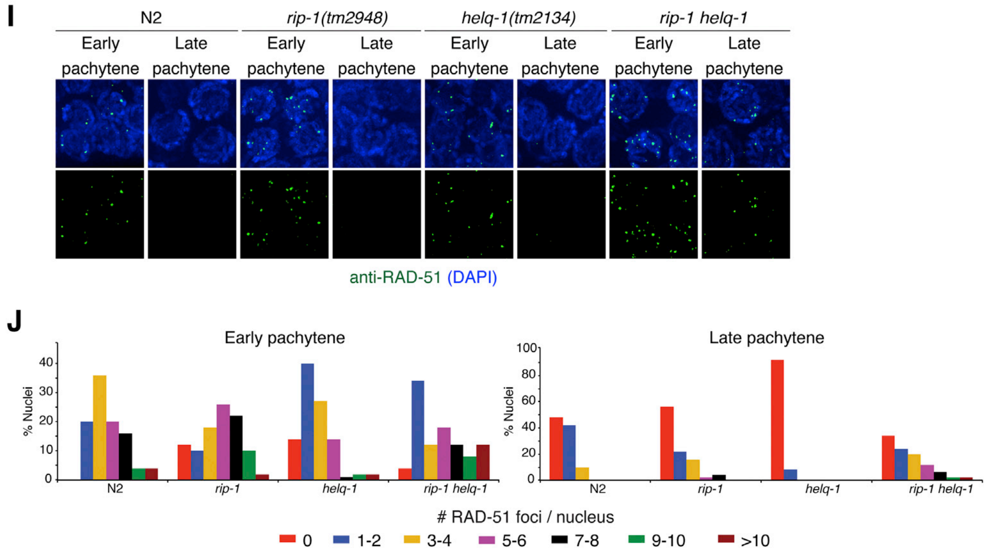

(legend on next page)

Figure S1. RIP-1 Is a Highly Divergent Rad51 Paralog that Forms a Complex with RFS-1, Related to Figure 1

(A) Expression of N-terminally 6xHis-, GST- or MBP-tagged RFS-1 in *E. coli* fails to produce soluble protein. Proteins were detected by Instant Blue staining. U, uninduced. I, induced. Sol, soluble. Induced RFS-1 fusions are indicated with asterisks.

(B) Reciprocal yeast two-hybrid matrix for RFS-1, RIP-1 and RAD-51. Interactions are indicated by positive β-galactosidase expression and survival on media containing 3-aminotriazole (3-AT) in the absence of histidine. Growth on media lacking leucine and tryptophan is a positive control for plasmid transfection.

(C) Expression and purification of RFS-1/RIP-1 mutant proteins in yeast. Western blots show whole cell TCA extracts before and 5 hr after 2% galactose induction. FLAG bead eluates from soluble extracts were detected by Instant Blue staining. IP, immunoprecipitate.

(D and E) Embryonic survival of progeny of animals treated with ionizing radiation (IR) (D) and nitrogen mustard (HN2) (E). Error bars indicate SEM from at least 24 adults over 2 independent experiments.

(F) Number of apoptotic corpses scored by SYTO12 staining in the gonads of animals treated with ionizing radiation (IR) or nitrogen mustard (HN2). Error bars indicate SEM from at least 28 adults over 2 independent experiments.

(G) Table of brood size, embryonic lethality and percent males for the indicated genotypes.

(H) Embryo viability of all progeny from 5-10 parental worms of the indicated genotype. Error bars indicate SD.

(I) RAD-51 immunofluorescence (green) from representative early and late pachytene nuclei of worm germlines from the indicated genotypes. DNA is stained with DAPI (blue).

(J) Quantification of (I) indicating the distribution of the number of RAD-51 foci per nucleus in early and late pachytene from each of the indicated genotypes. Foci were counted from 50 nuclei from 5 animals.

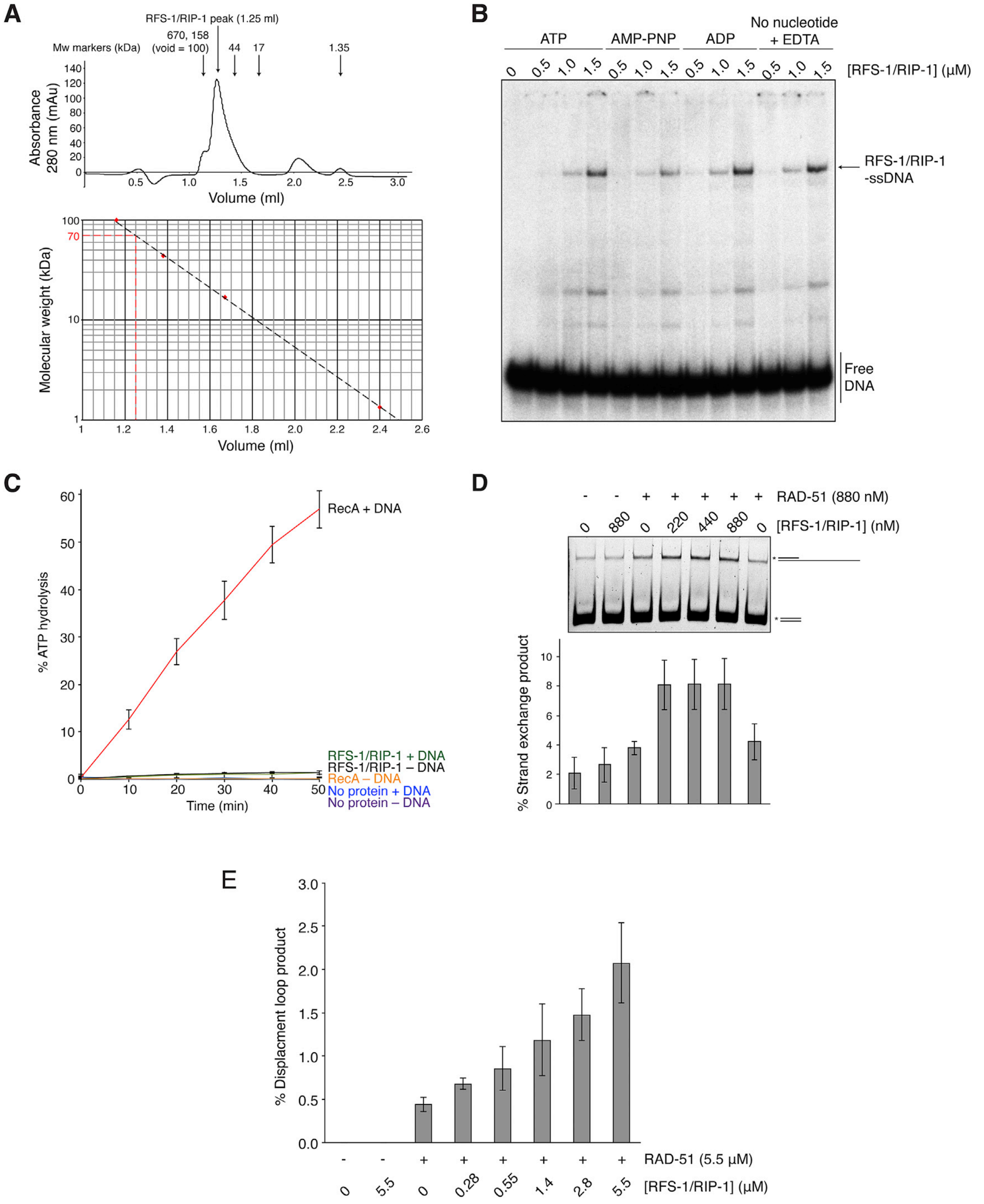

Figure S2. Biochemical Properties of RFS-1/RIP-1 Complex, Related to Figure 2

(A) Size exclusion chromatography trace from a 3 ml Superdex 75 5/150 GL column loaded with 30 μl RFS-1/RIP-1 from single-step FLAG immunoprecipitation. Arrows indicate molecular weight (Mw) markers. A plot of the $\log_{10}$(Mw) against elution volume for the markers allowed estimation of a size for the complex.

(B) EMSA showing RFS-1/RIP-1 ssDNA binding is nucleotide independent.

(C) RFS-1/RIP-1 lacks detectable ATPase activity in the presence or absence of DNA. Error bars indicate SD (n = 3). RecA + DNA is a positive control.

(D) RFS-1/RIP-1 stimulates strand exchange by RAD-51. Error bars indicate SD (n = 4).

(E) RFS-1/RIP-1 stimulates D loop formation by RAD-51 at the same RAD-51:DNA ratio as used in stopped flow experiments. Error bars indicate SD (n = 3).

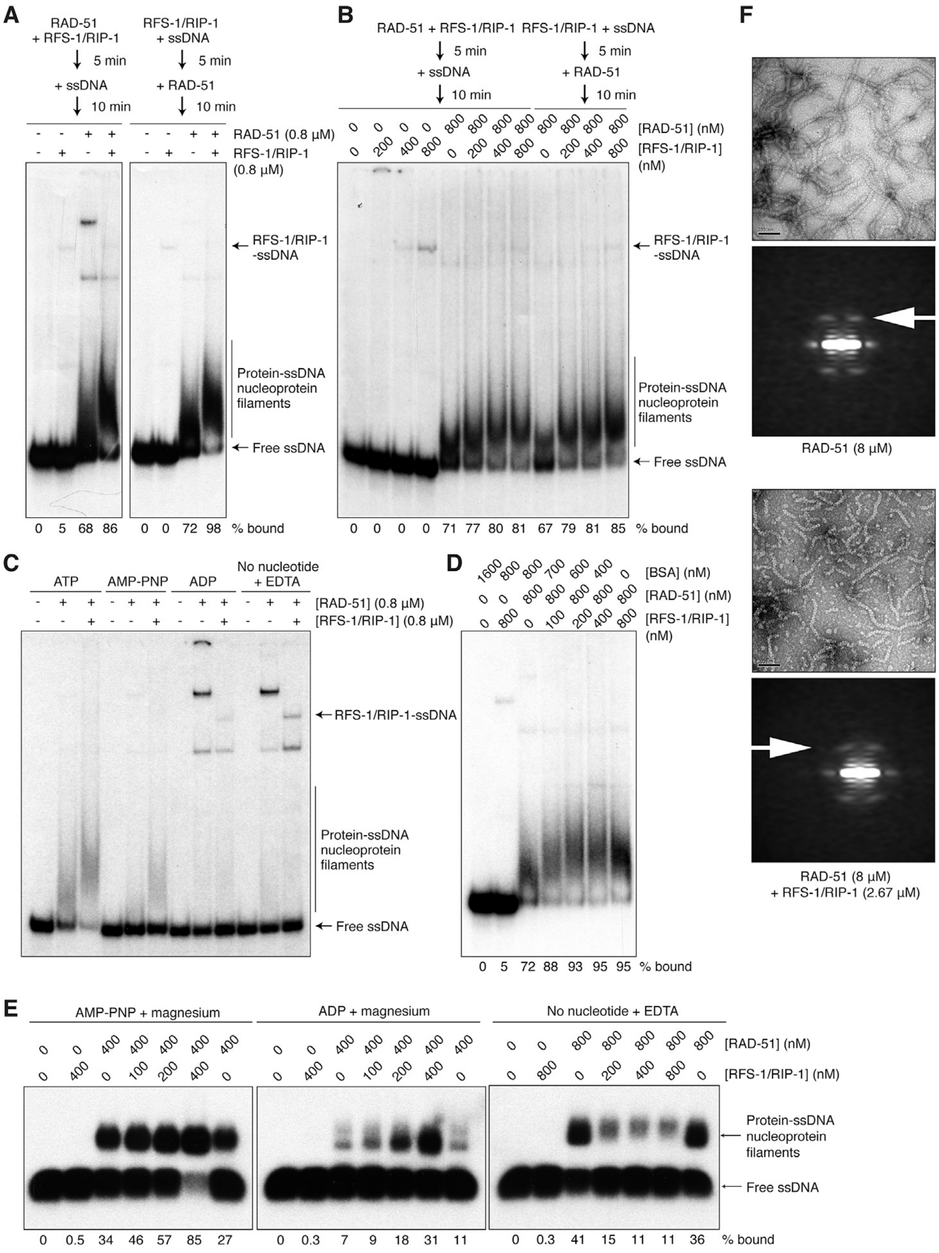

Figure S3. RFS-1/RIP-1 Binds and Modulates the Biochemical Properties of RAD-51-ssDNA Filaments, Related to Figure 3

(A) Protein-DNA complexes formed by RAD-51 and RFS-1/RIP-1 on ssDNA with ATP according to the mixing scheme indicated resolved by native PAGE.

(B) Protein-DNA complexes formed by RAD-51 and different concentrations of RFS-1/RIP-1 on ssDNA with ATP according to the mixing scheme indicated resolved by native PAGE.

(C) RAD-51 and RFS-1/RIP-1 were pre-incubated before addition of ssDNA with the indicated nucleotide, then protein-DNA complexes were resolved by native PAGE.

(D) The effect of RFS-1/RIP-1 on RAD-51-ssDNA filaments is specific and not due to changes in total protein level. RAD-51, RFS-1/RIP-1 and BSA were pre-incubated before addition of ssDNA with the indicated nucleotide, then protein-DNA complexes were resolved by native PAGE.

(E) Protein-DNA complexes formed by RAD-51 and RFS-1/RIP-1 on ssDNA with AMP-PNP, ADP or in the absence of nucleotide crosslinked with 0.25% glutaraldehyde and resolved by agarose gel electrophoresis.

(F) Electron micrographs (top) and averaged power spectra (bottom) of RAD-51-ssDNA filaments formed with and without RFS-1/RIP-1 used for attempted filament reconstructions. In the averaged power spectra, the arrows show that the layer line arising from the 1-start helix has the same spacing with and without RFS-1/RIP-1, and the average pitch in both cases is ~90 Å.

A

[RAD-51] (nM)	Δ Cy3 fluorescence (a.u.)	Half-time (s)
250	0.171 ± 0.0237	1.60 ± 0.668
500	0.322 ± 0.0187	0.0583 ± 0.0216
1000	0.346 ± 0.0180	0.0140 ± 0.00507

B

[RFS-1/RIP-1] (nM)	Δ Cy3 fluorescence (a.u.)	Half-time (s)
0	-0.0412 ± 0.0231	N.A.
20	-0.0733 ± 0.0178	N.A.
100	-0.150 ± 0.0130	12.4 ± 4.07
250	-0.197 ± 0.0258	11.0 ± 3.17
1000	-0.239 ± 0.0292	4.10 ± 1.87

E

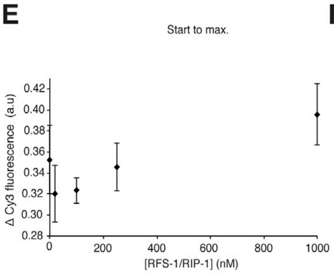

F

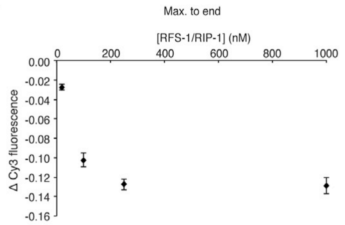

C

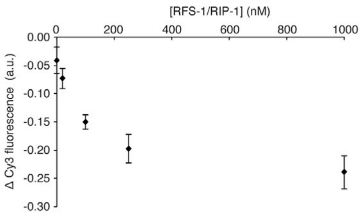

G

[RFS-1/RIP-1] (nM)	Δ Cy3 fluorescence (a.u.)
0	0.140 ± 0.0105
20	0.102 ± 0.0200
100	0.0778 ± 0.0262
250	0.0522 ± 0.0380
1000	0.0236 ± 0.0281

H

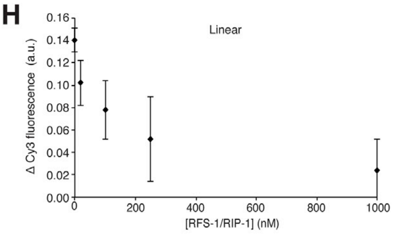

D

[RFS-1/RIP-1] (nM)	Time of max. (s)	Δ Cy3 fluorescence (a.u.) Start to max.	Δ Cy3 fluorescence (a.u.) Max. to end	Half-time (s) Start to max.	Half-time (s) Max. to end
0	N.A.	0.353 ± 0.0328	N.A.	0.0145 ± 0.00557	N.A.
20	7.87 ± 1.66	0.320 ± 0.0269	-0.0276 ± 0.00310	0.0111 ± 0.00244	22.4 ± 3.15
100	0.389 ± 0.127	0.323 ± 0.0121	-0.102 ± 0.00693	0.00826 ± 0.00119	18.2 ± 1.13
250	0.219 ± 0.133	0.346 ± 0.0228	-0.127 ± 0.00558	0.00847 ± 0.000778	11.2 ± 1.35
1000	0.273 ± 0.154	0.395 ± 0.0291	-0.129 ± 0.00828	0.00759 ± 0.00165	10.6 ± 0.629

I

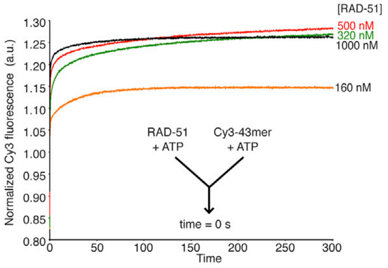

J

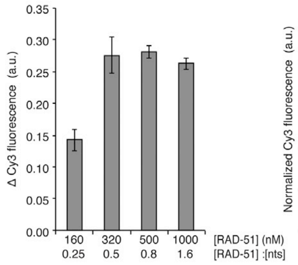

K

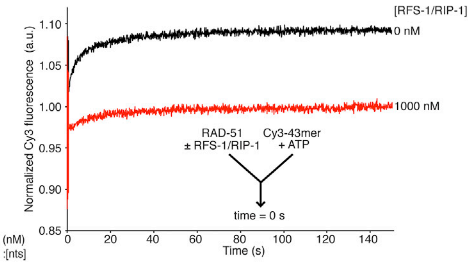

L

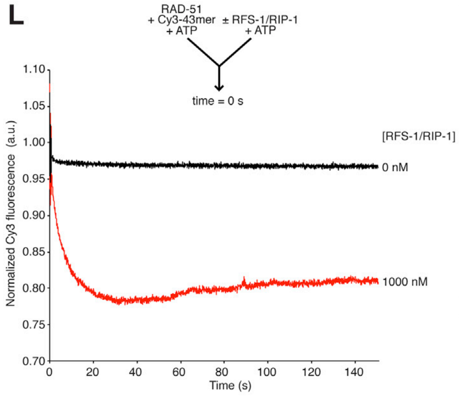

M

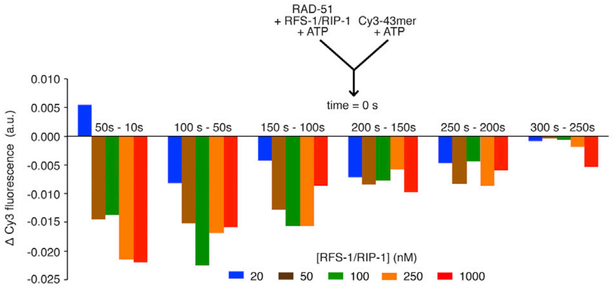

N

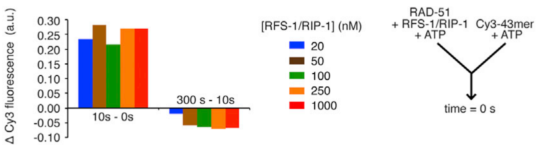

Figure S4. Analysis of Stopped-Flow Data for Remodeling of RAD-51-ssDNA Filaments by RFS-1/RIP-1, Related to Figure 4

(A–N) All fluorescence values for start, end and maximum fluorescence and corresponding time values were determined using ten-point moving averages calculated for all data sets (see the Supplemental Experimental Procedures for details).

(A) Table of average difference in normalized Cy3 fluorescence (Δ Cy3 fluorescence) and average time to reach half of the fluorescence change (half-time) over the full time course at different RAD-51 concentrations presented in Figure 4A. Error bars indicate SD (n = 8-9).

(legend continued on next page)

(B) Table of average Δ Cy3 fluorescence and half-time over the full time course at different RFS-1/RIP-1 concentrations presented in Figure 4C. Half-time values for 0 and 20 nM RFS-1/RIP-1 could not be determined since the initial noise in the dataset due to sample equilibration impairs reliable estimation. Error bars indicate SD (n = 6–9).

(C) Graph of average Δ Cy3 fluorescence as a function of RFS-1/RIP-1 concentration plotted in linear scale for the data presented in (B). Error bars indicate SD (n = 6–9).

(D) Table showing the average time the maximum in fluorescence is observed (Time of max.), and average Δ Cy3 fluorescence and half-times for both the first phase of increasing fluorescence (Start to max.) and the second phase of decreasing fluorescence (Max. to end) at different RFS-1/RIP-1 concentrations presented in Figure 4D. Errors indicate SD (n = 5–7).

(E and F) Graphs of Δ Cy3 fluorescence from start to max. (E) and max. to end (F) as a function of RFS-1/RIP-1 concentration for the data presented in (D). Error bars indicate SD (n = 5–7).

(G) Table of average Δ Cy3 fluorescence over the full time course at different RFS-1/RIP-1 concentrations presented in Figure 4E. Errors indicate SD (n = 6–9).

(H) Graph of average Δ Cy3 fluorescence as a function of RFS-1/RIP-1 concentration plotted in linear scale for the data presented in (G). Error bars indicate SD (n = 6–9).

(I) Analysis of average normalized Cy3-43-mer fluorescence plotted as a function of time over 300 s, for indicated concentrations of RAD-51 rapidly mixed with 15 nM Cy3-43-mer (both +ATP) at the 0 s time point in a stopped-flow instrument (indicated by arrow inset) (n = 3–4).

(J) Bar chart showing average Δ Cy3 fluorescence over the full time course at different RAD-51 concentrations presented in (A). Errors indicate SD (n = 3–4).

(K and L) Analysis of normalized Cy3-43-mer fluorescence plotted as a function of time over 150 s, demonstrating reactions attain equilibrium. The arrow (inset) indicates the components of the two syringes rapidly mixed at the 0 s time point in a stopped-flow instrument. (K) 1000 nM RAD-51 pre-incubated with indicated concentrations of RFS-1/RIP-1 for 10 min without ATP, then mixed with 15 nM Cy3-43-mer and ATP. (L) RAD-51-ssDNA filaments pre-formed with 1000 nM RAD-51 + 15 nM Cy3-43-mer for 10 min, then mixed with indicated concentrations of RFS-1/RIP-1 (both +ATP).

(M) Graphs of Δ Cy3 fluorescence for indicated time intervals over 300 s showing reactions reach equilibrium for an experiment where 1000 nM RAD-51 was pre-incubated with indicated concentrations of RFS-1/RIP-1 for 10 min, then mixed with 15 nM Cy3-43-mer (both +ATP).

(N) Graphs of Δ Cy3 fluorescence for time intervals corresponding to the filament formation phase (10 s – 0 s) and filament remodeling phase (300 s – 10 s) for the same experiment as in (M).

A

[RFS-1/RIP-1] (nM)	Δ Cy3 fluorescence (a.u.)
0	-0.0493 ± 0.00352
3.3	-0.0382 ± 0.00325
4.4	-0.0329 ± 0.00364
6.7	-0.0330 ± 0.00160
20	-0.0143 ± 0.00399
100	0.00393 ± 0.00326
250	0.0132 ± 0.00166
1000	0.00608 ± 0.00150

C

[RFS-1/RIP-1] (nM)	Δ Cy3 fluorescence (a.u.)
0	-0.142 ± 0.00297
3.3	-0.119 ± 0.00251
4.4	-0.117 ± 0.00167
6.7	-0.112 ± 0.00192
20	-0.0537 ± 0.00433
50	-0.0249 ± 0.00139
100	0.0304 ± 0.00275
250	0.0289 ± 0.00457

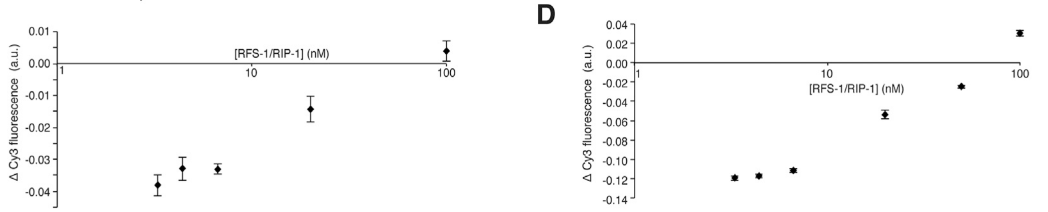

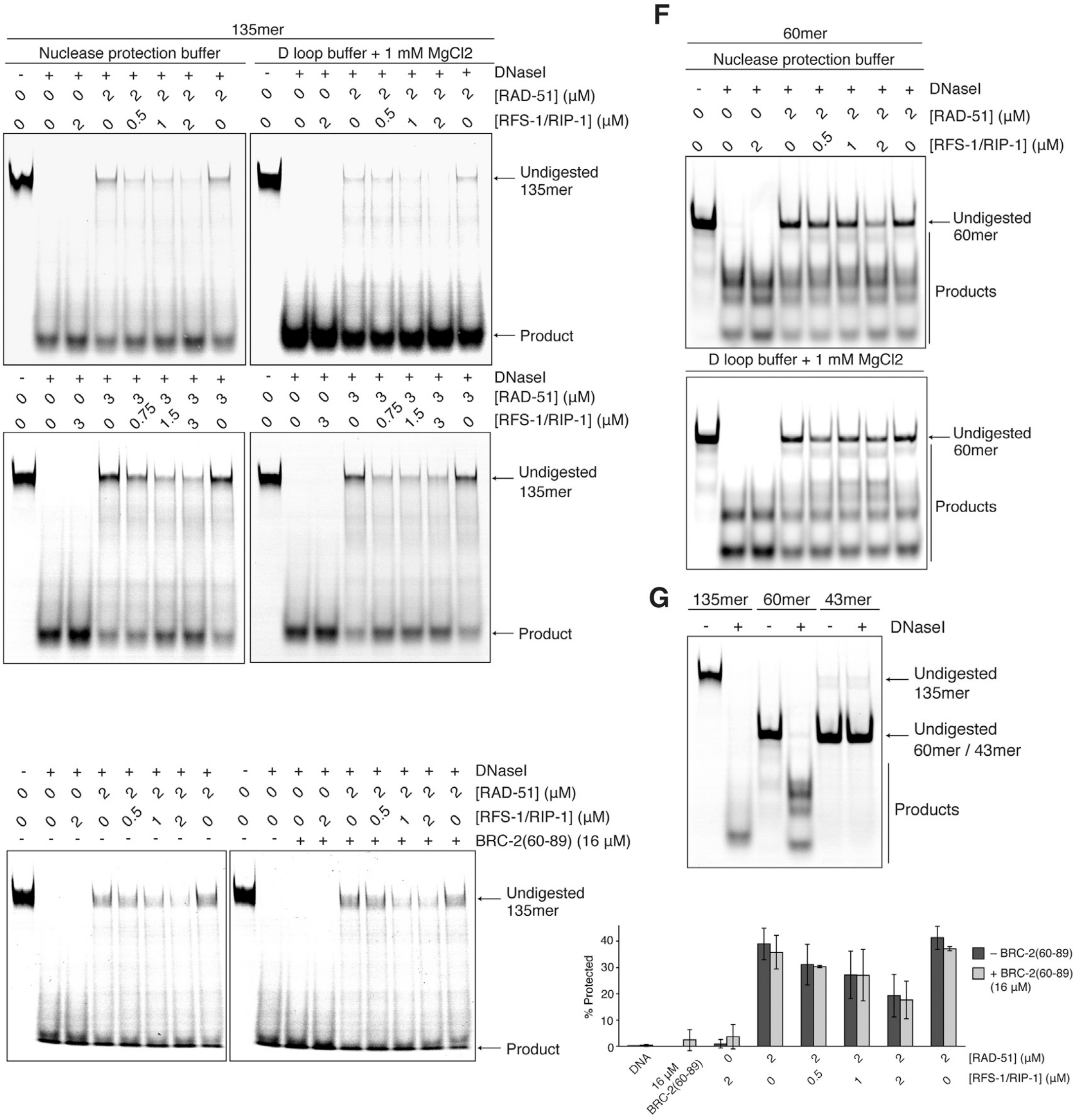

(legend on next page)

Figure S5. Analysis of Stopped-Flow Data for Stabilization of RAD-51-ssDNA Filaments by RFS-1/RIP-1 in a Nuclease-Sensitive Conformation, Related to Figure 5

(A) Table of average Δ Cy3 fluorescence at different RFS-1/RIP-1 concentrations for the data presented in Figure 5B (see the Supplemental Experimental Procedures for calculation details) using Cy3-43-mer DNA. Errors indicate SD (n = 4–6).

(B) Graph of average Δ Cy3 fluorescence as a function of RFS-1/RIP-1 concentration plotted in $\log_{10}$ scale for the data presented in (A). Error bars indicate SD (n = 4–6).

(C) Table of average Δ Cy3 fluorescence at different RFS-1/RIP-1 concentrations for the data presented in Figure 5C (see the Supplemental Experimental Procedures for calculation details) using Cy3-23-mer DNA. Errors indicate SD (n = 5–8).

(D) Graph of average Δ Cy3 fluorescence as a function of RFS-1/RIP-1 concentration plotted in $\log_{10}$ scale for the data presented in (C). Error bars indicate SD (n = 5–8).

(E and F) DNaseI protection assays on protein-DNA complexes formed by RAD-51 and RFS-1/RIP-1 on 135-mer (E) or 60-mer (F) ssDNA with ATP in either the same nuclease protection buffer as in Figure 5D or the D loop buffer (Figure 2C) supplemented with 1 mM $MgCl_2$.

(G) The 135-mer and 60-mer are substrates for DNaseI but the 43-mer used in stopped-flow is not efficiently digested by DNaseI.

(H) DNaseI protection assays on protein-DNA complexes formed by RAD-51 and RFS-1/RIP-1 in the presence or absence of BRC-2(60-89) peptide on 135-mer ssDNA with ATP (left, gels; right, quantification). Error bars indicate SD (n = 3).

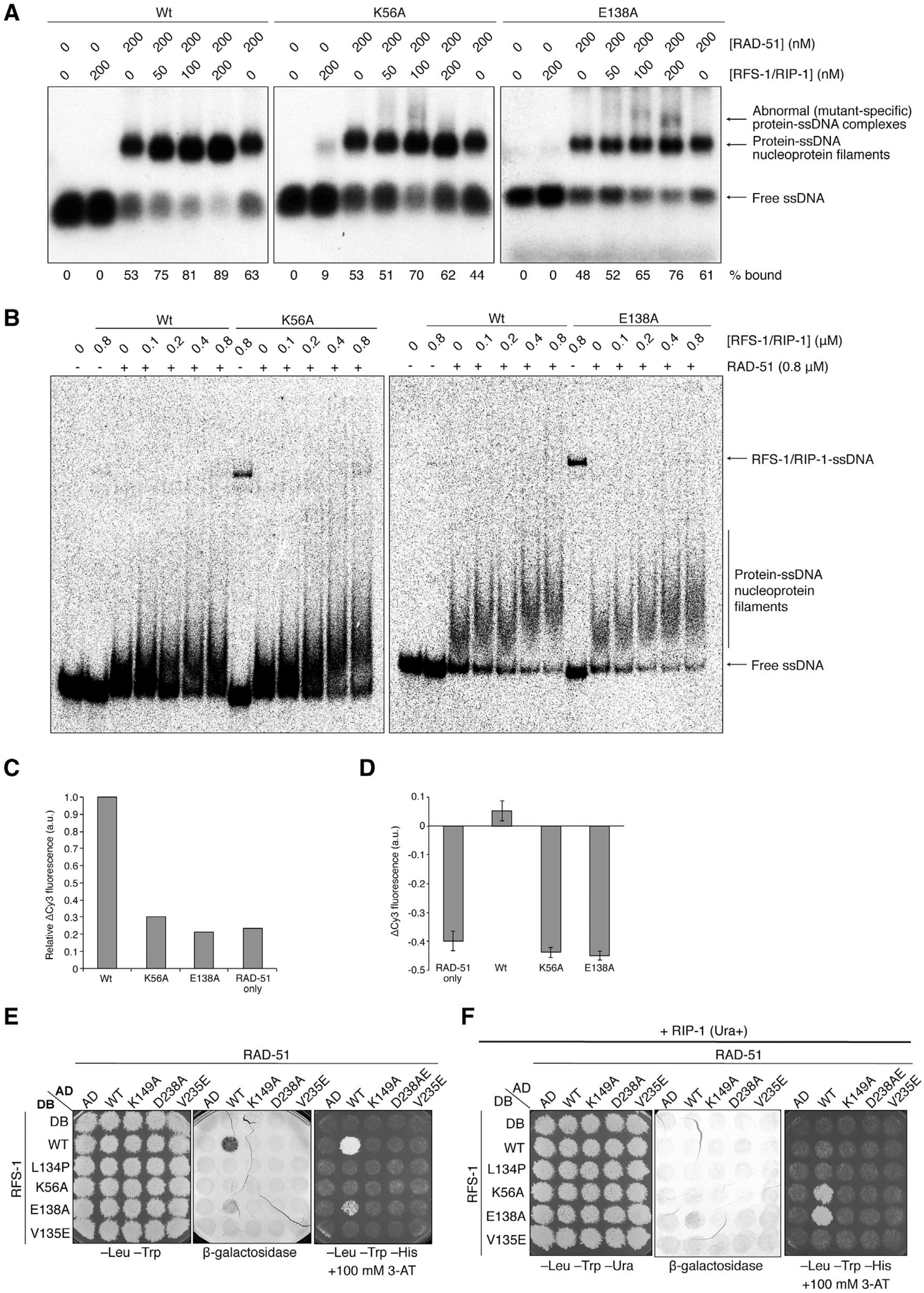

Figure S6. Walker Box Mutations in RFS-1 Are Permissive for Filament Binding but Prevent Remodeling, Related to Figure 6
(A) Protein-DNA complexes formed by RAD-51 and RFS-1/RIP-1 (Wt, K56A or E138A) on ssDNA with ATP. Proteins were pre-incubated before addition of ssDNA, then protein-DNA complexes crosslinked and resolved in agarose gels.

(legend continued on next page)

Cell

(B) Protein-DNA complexes formed by RAD-51 and RFS-1/RIP-1 (Wt, K56A or E138A) on ssDNA with ATP. Proteins were pre-incubated before addition of ssDNA and resolved by native page.
(C) Analysis of average Δ Cy3 fluorescence for K56A and E138A mutant complexes normalized to Wt for the data presented in Figures 6E and 6F (see the Supplemental Experimental Procedures for calculation details). Error bars indicate SD (n = 6–8).
(D) Analysis of average Δ Cy3 fluorescence for K56A and E138A mutant complexes for the data presented in Figures 6G and 6H (see the Supplemental Experimental Procedures for calculation details). Error bars indicate SD (n = 5–8).
(E) DB-RFS-1 and AD-RAD-51 interact via ATPase motifs in yeast two-hybrid, indicated by positive β-galactosidase expression and survival on media containing 3-aminotriazole (3-AT) in the absence of histidine. Growth on media lacking leucine and tryptophan is a positive control for plasmid transfection.
(F) RIP-1 was co-expressed in the same strains as in (E) on a Ura-selectable plasmid and modulates the RFS-1/RAD-51 interaction network. Growth on media lacking leucine, tryptophan and uracil is a positive control for plasmid transfection.

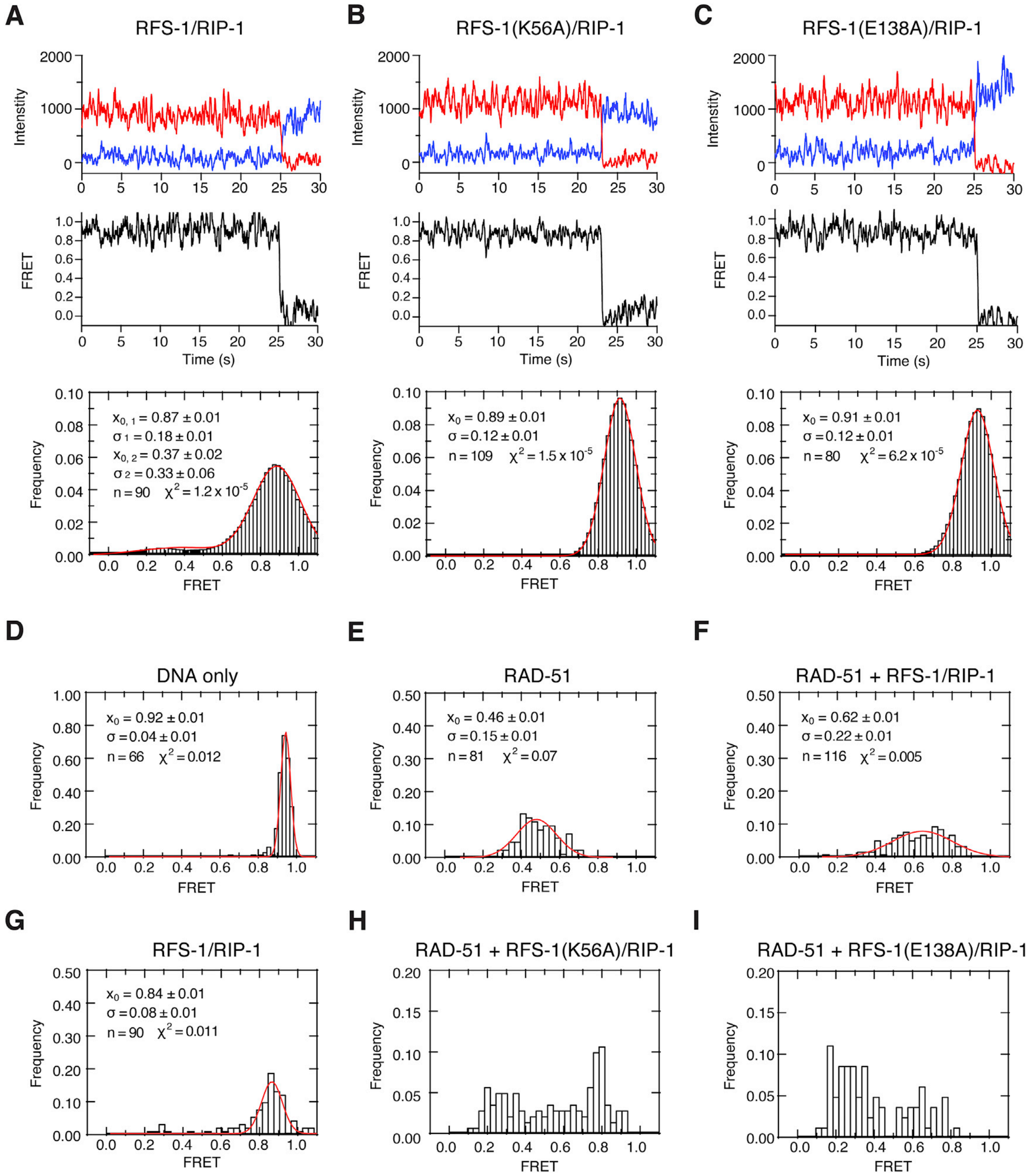

Figure S7. Remodeled RAD-51-ssDNA Filaments Are More Flexible, Related to Figure 7

(A–C) smFRET analysis of (A) DNA + RFS-1/RIP-1 (Wt), (B) DNA + RFS-1(K56A)/RIP-1, (C) DNA + RFS-1(E138A)/RIP-1. Top to bottom: cartoon schematic of the smFRET experiment indicating FRET between Cy3 and Cy5 (7 nt separation) attached to biotinylated DNA constructs immobilized on a streptavidin-coated biotin-PEG surface; donor (blue) and acceptor (red) intensity trajectories are anti-correlated until single-step photobleaching of the acceptor; FRET trajectories (black) between Cy3 and Cy5 exhibit a sharp drop to zero FRET when the acceptor photobleaches; histogram of mean FRET values for individual molecules with a Gaussian fit shown in red, where x_0 is the mean FRET, σ is the distribution width, and χ^2 quantifies the quality of the fit.

(D–I) Histograms of binned mean FRET values determined from each trajectory individually with Gaussian fits superimposed (red curves) (D) DNA only; (E) DNA + RAD-51; (F) DNA + RAD-51 + RFS-1/RIP-1 (Wt); (G) DNA + RFS-1/RIP-1 (Wt); (H) DNA + RAD-51 + RFS-1(K56A)/RIP-1; (I) DNA + RAD-51 + RFS-1(E138A)/RIP-1. Differences in the mean are statistically significant ($p < 0.0005$).

Cell

Supplemental Information

Rad51 Paralogs Remodel Pre-synaptic Rad51 Filaments to Stimulate Homologous Recombination

Martin R.G. Taylor, Mário Špírek, Kathy R. Chaurasiya, Jordan D. Ward, Raffaella Carzaniga, Xiong Yu, Edward H. Egelman, Lucy M. Collinson, David Rueda, Lumir Krejčí, and Simon J. Boulton

Supplemental Experimental Procedures

Cloning of protein expression constructs

Codon optimized cDNA encoding native RFS-1 and C-terminally 3xFLAG-tagged RIP-1 for expression in budding yeast were obtained from Geneart (Life Technologies). The sequence of the tag immediately downstream of RIP-1 is: LGGGGSGGGGDYKDDDDKDYKDDDDKDYKDDDDK. These constructs were cloned into a modified form of the pRS306 vector containing the bidirectional Gal1-10 promoter cloned between the AscI and SgrAI restriction sites, as described previously (Frigola et al., 2013). RFS-1 was cloned between the SgrAI and NotI restriction sites and RIP-1 between the AscI and SmaI restriction sites, and the construct integrated into yeast strain yJF1 (*W303-1a pep4::KanMx4 bar1::Hph-NT1 ade2-1 ura3-1 his3-11 trp1-1 leu2-3*) (gift of John Diffley and Jordi Frigola) after linearization at the NcoI unique restriction site in the URA3 cassette. Site directed mutagenesis to generate mutant expression constructs was performed using the QuikChange Site-Directed Mutagenesis Kit (Stratagene). Protein expression was confirmed in small scale cultures by growing cells in YP + 2% raffinose at 30°C until OD_{600} of 0.7-0.8, before induction for 5 h with 2% galactose. Samples of 5 x 10^8 cells were collected before and after induction, lysed with alkaline and proteins precipitated with trichloroacetic acid (TCA). The precipitate was harvested and resuspended in sample buffer and analysed by SDS-PAGE.

Codon optimized cDNA encoding native RAD-51 (short isoform) in *E. coli* was obtained from Geneart (Life Technologies) and cloned in the Champion pET-SUMO vector (Life Technologies) according to the manufacturer's instructions.

RFS-1/RIP-1 expression and purification

RFS-1/RIP-1 (Wt, K56A or E138A) were expressed in budding yeast cells by growing a 100 L culture in a 120 L BIOFLO 5000 fermenter (New Brunswick Scientific) in YP media + 2% raffinose at 30°C, pH 5.8, agitation 200 rpm and airflow 20 l/min until OD_{600} of 0.7-0.8, before induction for 5 h with 2% galactose. The cells were harvested and washed twice with Yeast Wash Buffer (25 mM HEPES-KOH (pH 7.5), 1 M sorbitol) then once with Buffer K (45 mM HEPES-KOH, 10% glycerol, 0.1 M potassium glutamate, 5 mM magnesium acetate) supplemented with 0.02% NP-40. The cells were then resuspended in 70 ml buffer K + 0.02% NP-40 supplemented with cOmplete, EDTA-free protease inhibitor cocktail tablets (Roche) (1 tablet per 25 ml buffer) and frozen dropwise in liquid nitrogen. Cells were lysed by crushing in a freezer mill (SPEX CertiPrep 6850) for 6 x 2 min cycles at rate 15 under liquid nitrogen and the frozen cell powder stored at –80°C. For purification, the cell

powder was thawed to room temperature in a water bath, diluted with Buffer K and the potassium glutamate concentration adjusted to 0.5 M in a total volume of 780 ml, and mixed well with a magnetic stirrer at 4°C until the lysate was homogenous. The lysate was cleared in an Optima LE-80K Ultracentrifuge (Beckman Coulter) using a Ti45 rotor at 40,000 rpm for 90 min at 4°C and the supernatant was applied to 10 ml bed volume of anti-FLAG M2 affinity gel (Sigma A2220), which had been pre-washed with 0.1 M glycine (pH 3.5), TBS and Buffer K according to the manufacturer's instructions. The protein was bound to the beads by rotating at 4°C overnight then applied to 5 x 25 ml batch purification columns. All subsequent steps were carried out at 4°C. The flow-through was discarded and the beads washed with 750 ml Buffer K (150 ml per column). The protein was eluted with 1 mg/ml 3xFLAG peptide (synthesised by Nicola O'Reilly, Peptide Chemistry Laboratory, The Francis Crick Institute) in Buffer K by passing 2 ml twice over each column then washing with 4 ml Buffer K per column (total eluate volume 30 ml), and dialyzed against 4 L Storage Buffer (20 mM Tris-acetate (pH 8.0), 100 mM potassium acetate, 10% glycerol, 1 mM EDTA, 0.5 mM DTT) overnight using 10 kDa MWCO SnakeSkin dialysis tubing (Thermo Scientific). The protein was then concentrated on a 30 kDa MWCO Amicon Ultra-15 Centrifugal Filter Unit pre-washed with water and Storage Buffer to a final volume of 1 ml, frozen in small aliquots in liquid nitrogen and stored at –80 °C. This method typically yielded 3-3.6 mg/ml recombinant RFS-1/RIP-1 (55-65 µM complex), devoid of detectable nuclease activity. Antibodies to detect RFS-1 and RIP-1 were raised in-house against peptides from each protein in rabbit or guinea pig.

RAD-51 expression and purification

For some EMSA experiments, RAD-51 was purified as described previously (Petalcorin et al., 2006). For all other experiments, RAD-51 was purified using the Champion pET-SUMO system (Life Technologies). The protein was expressed in BL21(DE3) One Shot *E. coli* by inoculating 6 L LB pre-warmed to 37 °C and supplemented with 50 µg/ml kanamycin to OD_{600} of ~0.01 from an overnight culture in 2TY grown at 30 °C. The culture was grown at 37 °C to OD_{600} of 0.6-0.8, before induction for 4 h with 1 mM IPTG at 30 °C. The cells were harvested at 4 °C and the pellets frozen on dry ice and stored at –80 °C. For purification, the pellets were thawed at 37 °C in a water bath and resuspended in 400 ml ice cold Lysis Buffer (50 mM potassium phosphate (pH 7.8), 1 M KCl, 10% glycerol) supplemented with cOmplete, EDTA-free protease inhibitor cocktail tablets (Roche) (1 tablet per 25 ml buffer), and mixed well with a magnetic stirrer at 4 °C until the mixture was homogenous. The cells were lysed by addition of Triton X-100 to 0.1% on the stirrer. The lysate was transferred to 4 x 100 ml flasks and each flask sonicated on ice 3 x 2 min with a large flat tip using a Branson Sonifier 450 (duty

cycle 80%, output control 8). The lysate was cleared in an Optima LE-80K Ultracentrifuge (Beckman Coulter) using a Ti45 rotor at 40,000 rpm for 60 min at 4 °C. Imidazole was added to the supernatant to a final concentration of 25 mM and applied to 12 ml bed volume of Ni-NTA agarose affinity gel (Qiagen 30210), which had been pre-washed with Binding Buffer (50 mM potassium phosphate (pH 7.8), 1 M KCl, 10% glycerol, 25 mM imidazole (pH 7.5)). The protein was bound to the beads by rotating at 4 °C for 2 h then applied to 6 x 25 ml batch purification columns. All subsequent steps were carried out at 4 °C. The flowthrough was discarded and the beads washed with 300 ml Binding Buffer and 300 ml Binding Buffer containing 50 mM imidazole (50 ml per column). The protein was eluted with Binding Buffer containing 200 mM imidazole by passing 8 ml twice over each column then washing with 4 ml Buffer K per column (total eluate volume 72 ml), and dialyzed against 4 L Dialysis Buffer (20 mM Tris-HCl (pH 8.0), 300 mM KCl, 10% glycerol) overnight using 10 kDa MWCO SnakeSkin dialysis tubing (Thermo Scientific). Some precipitate was observed the following day, but most protein remained in solution. The His-SUMO tag was cleaved to yield native RAD-51 by addition of 6 μl His-tagged Ulp1 SUMO protease (gift from Peter Cherepanov) for 45 min. The protein was centrifuged and the soluble fraction collected and bound to the same batch of NiNTA agarose affinity gel used for purification after regeneration according to the manufacturer's instructions to remove the SUMO protease and His-SUMO tag. The flowthrough containing native RAD-51 was collected and the resin washed with an additional 18 ml (3 ml per column) of Dialysis Buffer. These were pooled (total 90 ml) and mixed at 1:1 ratio with Dilution Buffer (20 mM Tris-HCl (pH 8.0), 10% glycerol, 2 mM EDTA, 1 mM DTT) (total 180 ml) to reduce salt concentration to 150 mM KCl. The protein was bound to a 1 ml Mono Q 5/50 GL column (GE Healthcare) at 0.5 ml / min using an Äkta Explorer HPLC system and washed with 30 ml R buffer (20 mM Tris-HCl (pH 8.0), 10% glycerol, 1 mM EDTA, 0.5 mM DTT) supplemented with 100 mM KCl then 20 ml R buffer supplemented with 150 mM KCl. The protein was eluted with a 30 ml gradient 150-640 mM KCl in R buffer and 0.5 ml fractions collected. The peak fractions were pooled and either dialysed against Storage Buffer for 1 h, frozen in small aliquots in liquid nitrogen and stored at –80 °C, or concentrated and frozen directly in the elution buffer. This method typically yielded 1.5-2.5 mg/ml recombinant RAD-51 (40-65 μM), which could be concentrated as high as 550 μM without precipitation.

Analytical size exclusion chromatography

30 μl of RFS-1/RIP-1 was loaded onto a 3 ml Superdex 75 5/150 GL column at 0.1 ml/min using an Ettan LC system and 0.1 ml fractions collected. Migration of molecular weight standards of 670, 158, 44, 17, 1.35 kDa (Biorad 151-1901) allowed estimation of a molecular weight for the complex.

RFS-1 expression in *E. coli*

N-terminal 6xHis, GST and MBP fusions of RFS-1 were cloned using the Gateway system under the control of the T7 promoter. Constructs were transformed into BL21(DE3) *E. coli* strains. Protein expression was performed by growing cells at 30°C until OD_{600} of 0.6, before induction for 4 h with 1 mM IPTG. Soluble extracts were prepared by harvesting cells, resuspending in 20 mM Tris-HCl (pH 7.4), 200 mM NaCl, 1 mM EDTA (pH 8.0), sonicating, then clearing the lysate at 4°C.

EMSA

Proteins were diluted from concentrated stocks into Storage Buffer, which was also used in no protein controls. For native polyacrylamide gels, proteins were mixed with a master mix (containing 100 nM (nucleotides) 5'-[^{32}P]-labelled 60mer oligonucleotide (ACGCTGCCGAATTCTACCAGTGCCTTGCTAGGACATCTTTGCCCACCTGCAGGTTCACCC), 20 mM Triethanolamine-HCl (pH 7.5), 8% glycerol, 1 mM DTT, 50 mM sodium acetate, and either 2 mM $MgCl_2$ and 2 mM of the indicated nucleotide, or 10 mM EDTA (pH 8.0)) according to the indicated scheme in 10 μl reaction volume at 25°C. Double-stranded DNA was prepared by annealing the above 60mer to its reverse complement. The reactions were transferred to ice and resolved on 8% native PAGE in 1X TBE using a Protean II xi cell system (Biorad) (200 V, 4 h 20 min, 4°C). For agarose gels, proteins were pre-incubated for 5 min then mixed with a master mix as above for 10 min, before crosslinking with 0.25% glutaraldehyde for 10 min, all at 25°C. In competition experiments, the same 60mer was added in unlabelled form in the indicated molar excess after protein-DNA complex formation for a further 10 min. Reactions were resolved on 1% agarose gels in 1X TBE (70 V, 2 h 20 min). Gels were dried and imaged by autoradiography or using a storage phosphor screen (Amersham Biosciences) and a Storm 840 molecular imager (Molecular Dynamics) and quantified using ImageQuant TL (Amersham Biosciences).

For immuno-shift experiments, proteins were incubated with 600 nM (nucleotides) of a 5'-fluorescein labelled version of the same 60mer for 10 min, before incubating protein-DNA complexes with anti-FLAG antibody (Sigma F3165) for 5 min and resolving in native agarose gels.

Nuclease protection assays

Proteins were diluted from concentrated stocks into T Buffer (25 mM Tris-HCl (pH 7.5), 10% glycerol, 0.5 mM EDTA (pH 8.0), 50 mM KCl), which was also used in no protein controls. Proteins were pre-incubated for 5 min then mixed with 7310 nM (nucleotides) 5'-fluorescein-labelled 135mer oligonucleotide (AGCTACCATGCCTGCACGAATTAAGCAATTCGTAATCATGGGTCAAAATCAATCTAAAGT ATATATGAGTAAACTTGGTCTGACAGTTACCAATGCTTAATCAGTGAGGCACCTATCTCAG CGATCTGTCTATTT) or a 5'-fluorescein-labelled version of the 60mer used in EMSA in either nuclease protection buffer (20 mM Tris-HCl (pH 7.5), 8% glycerol, 1 mM DTT, 50 mM sodium acetate, 2 mM ATP, 2 mM $MgCl_2$, 0.5 mM $CaCl_2$) or D loop Buffer (see below) supplemented with 1 mM ATP, 1 mM $MgCl_2$, 1 mM $CaCl_2$, in 10 µl reaction volume at 25°C for 10 min. 1 µl (2 U) bovine pancreatic DNaseI (New England Biolabs) was then added for 20 min at 25°C. The samples were deproteinized with 0.125% SDS and 12.5 µg proteinase K for 10 min at 37°C and resolved in 10% polyacrylamide gels in 1X TBE (110 V, 60 min). Gels were imaged on a FLA-9000 scanner (Fujifilm) and quantified with Multi Gauge V3.2 (Fujifilm) or ImageQuant TL (Amersham Biosciences). To calculate the normalized protection relative to RAD-51 alone (Figure 5E) the percentage of DNA binding at different RFS-1/RIP-1:RAD-51 ratios was determined from the native PAGE EMSA experiment in Figure S3B. The percentage of DNA binding and percentage of DNA protected in the EMSA and nuclease protection assays, respectively, were normalized such that the values for RAD-51 alone in each case were 1, and the normalized relative protection for each concentration of RFS-1/RIP-1 determined as the ratio of these values. This accounts for the underestimate in DNaseI deprotection due to the concomitant increase in protein-ssDNA complex formation in the presence of RFS-1/RIP-1.

D-loop formation assays

RAD-51 and RFS-1/RIP-1 were diluted from concentrated stocks into T Buffer (25 mM Tris-HCl (pH 7.5), 10% glycerol, 0.5 mM EDTA (pH 8.0), 50 mM KCl) or D-loop Buffer (10 mM Tris-HCl (pH 7.5), 50 mM KCl, 1 mM DTT) respectively, which were also used in no protein controls. Proteins were pre-incubated for 5 min then mixed with either 50 nM (moles) (Figure 2C, D) or 38.2 nM (moles) (Figure S2E) 5'-fluorescein-labelled 90mer oligonucleotide (AAATCAATCTAAAGTATATATGAGTAAACTTGGTCTGACAGTTACCAATGCTTAATCAGT GAGGCACCTATCTCAGCGATCTGTCTATTT), 1 mM ATP or the indicated nucleotide, 1 mM $CaCl_2$ in D-loop Buffer at 25 °C for 10 min. 2 µl (920 ng) pBluescript SK(–) (460 ng / µl) was then added to bring the final reaction volume to 10 µl and incubated for 15 min. The samples were

deproteinized with 0.1% SDS and 10 μg proteinase K for 10 min at 37 °C and resolved in 0.9% agarose gels in 1X TAE (90 V, 35 min). Gels were imaged on a FLA-9000 scanner (Fujifilm) and quantified with Multi Gauge V3.2 (Fujifilm).

Strand exchange assays

40mer dsDNA was prepared by annealing 5'-fluorescein-labelled 40mer oligonucleotide (TAATACAAAATAAGTAAATGAATAAACAGAGAAAATAAAG) to the complementary unlabelled 40mer oligonucleotide (CTTTATTTTCTCTGTTTATTCATTTACTTATTTTGTATTA) in 50 mM Tris-HCl (pH 7.5), 100 mM NaCl, 10 mM $MgCl_2$, and stored at stock concentration 200 nM (moles). Proteins were diluted from concentrated stocks into T Buffer (25 mM Tris-HCl (pH 7.5), 10% glycerol, 0.5 mM EDTA (pH 8.0), 50 mM KCl), which was also used in no protein controls. Proteins were pre-incubated for 5 min then mixed with 5.6 nM (moles) 150mer oligonucleotide (TCTTATTTATGTCTCTTTTATTTCATTTCCTATATTTATTCCTATTATGTTTTATTCATTTACT TATTCTTTATGTTCATTTTTTATATCCTTTACTTTATTTTCTCTGTTTATTCATTTACTTATTT TGTATTATCCTTATCTTATTTA), 50 mM Tris-HCl (pH 7.5), 1 mM DTT, 100 μg/ml of BSA, 2 mM ATP, 4 mM $CaCl_2$ in 12.5 μl reaction volume at 25 °C for 10 min. 0.5 μl dsDNA stock and 0.5 μl 0.1 M spermidine were then added incubated for 1 h. The samples were deproteinized with 0.1% SDS and 12.5 μg proteinase K at 37 °C and resolved in 10% polyacrylamide gels in 1X TBE (80 V, 1 h 15 min). Gels were imaged on a FLA-9000 scanner (Fujifilm) and quantified with Multi Gauge V3.2 (Fujifilm).

Stopped-flow assays and data analysis

Stopped-flow experiments were performed using an SFM-300 stopped-flow machine (Bio-Logic) fitted with a MOS-200 monochromator spectrometer (Bio-Logic) with excitation wavelength set at 545 nm. Fluorescence measurements were collected with a 550 nm long pass emission filter. The machine temperature was maintained at 25 °C with a circulating water bath.

For all experimental setups, a master mix containing all common reaction components for each of the two syringes was prepared to which variable components were added to generate the mixtures for individual syringes for different experimental conditions. These individual syringe mixtures are indicated in the mixing schemes. Since equal volumes were injected into the mixing chamber from each syringe, the two solutions became mutually diluted. Therefore, all reaction components common to each syringe were prepared at the final concentration, whereas reaction components present in only one syringe were added at twice the desired final concentration. All concentrations quoted represent final concentrations after mixing. Components of each syringe were pre-incubated for 10 min before

the start of experiments to allow the contents to reach equilibrium. Therefore in competition experiments with scavenger DNA, RAD-51-ssDNA filaments pre-formed in the presence of RFS-1/RIP-1 had already undergone remodelling, which is largely completed within 30-60 s, as judged by other experiments.

All reactions were performed in Stopped Flow Buffer (50 mM Tris-HCl (pH 7.5), 5 mM $MgCl_2$, 50 mM NaCl, 2 mM ATP), except in Figure 4E, where ATP was only pre-incubated with DNA, giving a final concentration of 1 mM. All reactions contained 15 nM (moles) 5'-Cy3 fluorescently labelled $(dT)_{43}$ or $(dT)_{23}$ oligonucleotide (Cy3-43mer or Cy3-23mer). Proteins were added directly from concentrated stocks. Unlabelled $(dT)_{43}$ or $(dT)_{23}$ oligonucleotide was used at 1500 nM (moles) (100-fold excess) in competition experiments. For all experiments, controls were also performed for buffer alone with and without DNA to confirm fluorescence signal stability over the time course of the experiments (data not shown). We verified that the theoretical minimum concentration of RAD-51 (320 nM) required to fully coat all molecules of DNA (640 nM nucleotides in stopped-flow) with filaments was sufficient to induce a maximal change in Cy3 fluorescence over longer time courses, whilst sub-saturating concentrations of RAD-51 (<320 nM) gave intermediate changes in Cy3 fluorescence (Figure S4I, J). Successively increasing RAD-51 concentration above 320 nM increased the rate of binding to ssDNA and attainment of equilibrium (Figure S4I). For experiments on pre-formed RAD-51 filaments we employed a saturating concentration of RAD-51 (1 μM), to ensure all ssDNA present was coated by RAD-51 filaments.

Fluorescence measurements for most experiments were collected according to the following protocol: (1) every 0.00005 s from 0-0.05 s; (2) every 0.0005 s from 0.05-0.56 s; (3) every 0.02 s from 0.56-60.54 s. For longer time courses (150 or 300 s), measurements were collected every 0.05 s during stage (3).

For each condition analysed, traces were collected from between three and nine independent reactions (n = 3-9, see individual figure legends for details). For presentation, average traces for each experiment were generated, except for some longer time course (150-300 s) experiments where single traces were analysed. Importantly, all conclusions from this study are made based on the magnitude and rate of changes in fluorescence over time and how these vary with RFS-1/RIP-1 concentration, and therefore the absolute fluorescence values were converted to arbitrary units by a normalization procedure to facilitate comparison. For all experiments the raw data were normalized to the same fluorescence value for the 0 s time point, except for the unlabelled DNA competition experiments (Figure 5, 6G, H) where raw data were normalized differently. In these experiments, an initial increase in fluorescence was observed in most samples, which reached a maximum at approximately 2 s,

particularly for the 43mer experiments. This is also observed for human Rad51 (M.S. and L.K., unpublished data) and therefore represents a systematic artefact of reaction equilibration upon mixing pre-assembled ATP-bound filaments with competitor DNA. Traces were therefore normalized to the same value for Cy3 fluorescence at the 2.01998 s time point and truncated before this to eliminate these artefacts. These adjustments were to aid comparison of protein concentration-dependent trends in the change in fluorescence over time.

For analysis, for all experiments in Figure 4 and 6E, F ten-point moving averages were calculated on each individual normalized trace, which were used to define initial (0 s), final (60.54 s), maximum fluorescence (and corresponding time point) and Δ Cy3 fluorescence values for each experiment. Half-times for different phases were also measured from time points where the fluorescence from moving averages was closest to the value calculated for Δ Cy3 fluorescence midpoints. Average values and associated standard deviations were then calculated. For competition experiments (Figure 5B, C and 6G, H), the relatively small changes in fluorescence observed meant the data was too noisy for analysis from moving averages for Δ Cy3 fluorescence and half-times in this way. For these experiments, Δ Cy3 fluorescence values were calculated on each individual normalized trace as the difference between the mean fluorescence values measured across all time points from 1.01998 to 2.99998 s (start value, on either side of the maximum at ~2 s) and 58.54 to 60.54 s (end value). Average values and associated standard deviations were then calculated. For data analysis of 300 s reactions (Figure S4M, N), estimates of fluorescence values at a given time point were determined as the mean fluorescence values measured across all time points ±1 s on individual traces. Positive and negative Δ Cy3 fluorescence values represent increases and decreases in fluorescence respectively.

Electron microscopy

For attempted filament reconstructions, 8 µM RAD-51 was incubated ± 3 µM RFS-1/RIP-1 with M13 ssDNA (USB) at RAD-51:DNA ratio 80:1 (w/w) in 25 mM triethanolamine-HCl (pH 7.2), 1.25 mM ATP, 1.25 mM NaF, 1.25 mM $Al(NO_3)_3$, 2 mM magnesium acetate at 30 °C for 30 min. Samples were applied to glow-discharged continuous carbon-coated grids, negatively stained by 2% uranyl acetate, and imaged on an FEI Tecnai T12 microscope at 80 keV with a nominal magnification of ×30,000. Images were recorded on Kodak SO163 film and digitized using a Nikon Coolpix 8000 scanner at a raster of 4.16 Å per pixel. The EMAN software package was used to extract filament images from micrographs, and the SPIDER software package was used for most image processing.

Immuno-gold labeling experiments were performed similarly to described previously (Shahid et al., 2014). Anti-FLAG antibody (Sigma F3165) was conjugated to 20-nm gold particles using the InnovaCoat Gold Conjugation Mini kit (Innova Biosciences). 1 μM RAD-51 ± 0.5 μM RFS-1/RIP-1 and 1 μM (nucleotides) of linearized PhiX ssDNA (gift of Michael Mcilwraith and Stephen West) were co-incubated in 20 mM Triethanolamine-HCl (pH 7.5), 8% glycerol, 1 mM DTT, 50 mM sodium acetate, 2 mM $MgCl_2$, 2 mM ATP for 10 min at 25 °C, before incubation with anti-FLAG-20-nm gold for a further 30 min at 25 °C. Samples were imaged by negative stain as described (Shahid et al., 2014). For quantification, five positions were counted for each grid (at the four cardinal directions and one centrally). Discrete filaments >50 nm in length (RAD-51: n = 644; RAD-51 + RFS-1/RIP-1: n = 588) were counted from each of 93 (RAD-51) or 112 (RAD-51 + RFS-1/RIP-1) fields of view, each an image of size 2120 nm x 1511 nm at nominal magnification 67,000X, and scored for number bound with anti-FLAG-20-nm gold on the end or along the filament length. Filament length was also determined for filaments associated with anti-FLAG-20-nm gold. Statistical analysis was performed in GraphPad Prism 6 (GraphPad Software).

Single molecule FRET (smFRET)

DNA oligonucleotides were ordered from Operon, and labelled with Cy3 or Cy5 using an NSH-ester modification (GE healthcare) reaction with amino-modified C6-dT. The sequence of the Cy3 anchor DNA is 5′-Cy3-CGCGAGGAATGGATGTAGGG-biotin-3′. The sequence of the Cy5 poly-dT7 DNA is 5′-CCCTACATCCATTCCTCGCGTTTTTT(Cy5-T)TTTTTTTTTTTTTTTTTTTTTTTTTT TTTTTTTTTTTTTTTTTTTTTTTTTTTTTTTTTTTTTT-3′. The labelling reaction was performed with 1 nmol DNA dissolved in 44 μL of 100 mM sodium tetraborate buffer, pH 8.5, and 10 nmol of Cy3 or Cy5 dissolved in 7 μL of DMSO. The reaction was incubated at room temperature overnight to obtain ≥80% labelled DNA. Labelled and unlabelled DNA fractions were separated by reverse phase HPLC chromatography on an analytical C18-column. Purified oligonucleotides were stored at –20 °C in 10 mM Tris-HCl, pH 8.0.

Single molecule FRET experiments were performed as previously described (Senavirathne et al., 2012). Briefly, quartz microscope slides were passivated with methoxy-PEG-SVA (M_r = 5,000; Laysan Bio Inc.) doped with 10% biotin-PEG-SVA (M_r = 3,400; Laysan Bio Inc.) to minimize non-specific binding to the surface. First, the reaction chamber was incubated with 0.2 mg/ml BSA (Sigma) in T50 buffer (50 mM Tris-HCl (pH 7.0), 50 mM NaCl) for 10 min, and then excess BSA was washed out with T50 buffer. Second, streptavidin (1 mg/ml in T50 buffer) was incubated for 10 min, and then

excess streptavidin was washed out with buffer (50 mM Tris-HCl (pH 7.5), 50 mM NaCl, 5 mM $MgCl_2$, 2 mM Trolox). Third, the ssDNA construct was surface immobilized. The DNA construct was prepared by annealing 1 µM each of the 3′-biotin 5′-Cy3 anchor and the Cy5 polydT7 DNA (Figure 7) in buffer (50 mM Tris-HCl (pH 7.5), 50 mM NaCl, 5 mM $MgCl_2$, 2 mM Trolox) by heating to 90 ˚C for 45 s and cooling to room temperature over 15 min, followed by serial dilutions to 25-50 pM DNA for surface immobilization. Excess DNA was washed out after a 10 min incubation. Finally, the proteins for each experiment (RAD-51, RFS-1/RIP-1, RFS-1(K56A)/RIP-1, and RFS-1(E138A)/RIP-1, 1 µM each) were introduced in imaging buffer (50 mM Tris-HCl (pH 7.5), 50 mM NaCl, 5 mM $MgCl_2$, 2 mM ATP, 0.2 mg/mL BSA, 2 mM Trolox to prevent photoblinking of the dyes, and an oxygen scavenger system consisting of 2.5 mM 3,4-dihydroxybenzoic acid (PCA) (Sigma) and 250 nM protocatechuate dioxygenase (PCD) (Sigma) to minimize photobleaching). Data was acquired on a home-built, prism-based single-molecule Total Internal Reflection Fluorescence (smTIRF) microscope at 30 ms time resolution. Movies were recorded for 5 min from 10 different areas of the slide. Apparent energy transfer efficiencies were calculated as FRET = $I_A/(I_D + I_A)$, where I_A is the acceptor intensity and I_D is the donor intensity.

ATPase assays

0.5 µM of RFS-1/RIP-1 or RecA (New England Biolabs) were mixed with or without 100 nM (moles) φX174 viron ssDNA (New England Biolabs) in 20 mM Triethanolamine-HCl (pH 7.5), 8% glycerol, 1 mM DTT, 50 mM sodium acetate, 2 mM $MgCl_2$, 2 mM ATP, 0.6 µCi $\gamma[^{32}P]$-ATP (6000 Ci/mmol) in 60 µl total volume at 37 °C. 8 µl samples were taken at the indicated time points and the reaction stopped with 4 µl 0.5 M EDTA (pH 8.0). 1.5 µl of each sample were loaded onto Polygram CEL 300 PEI thin layer chromatography plates (Macherey Nagel 801053), separated in 0.5 M LiCl, 1 M formic acid, and imaged using a storage phosphor screen (Amersham Biosciences) and a Storm 840 molecular imager (Molecular Dynamics).

Yeast two-hybrid

Yeast two-hybrid was performed as described previously (Boulton et al., 2002). N-terminal fusions of the DNA binding (DB) and transactivation (AD) domains of yeast Gal4 to RFS-1 and RIP-1 were cloned using the Gateway system and site-directed mutagenesis performed using the QuikChange Site-Directed Mutagenesis Kit (Stratagene).

GST pull-downs from human cells

N-terminal GST and FLAG fusions of RFS-1 and RIP-1 were cloned using the Gateway system under the control of the CMV promoter. Plasmids were transfected into 5 x 10^6 human 293T cells with Lipofectamine 2000 and cells were harvested 24 h later by scraping in Benzonase Lysis Buffer (20 mM Tris-HCl (pH 7.5), 40 mM NaCl, 2 mM $MgCl_2$, 0.5% NP-40, 50 U / ml benzonase (Novagen), cOmplete, EDTA-free protease inhibitor cocktail tablets (Roche) (1 tablet per 25 ml buffer)). After incubation for 10 min on ice, NaCl concentration was increased to 150 mM and the lysate rested on ice for a further 30 min with occasional vortexing. The lysate was cleared and the soluble fraction (4 mg protein) applied to 30 µl glutathione sepharose 4 fast flow beads (GE Healthcare 17-5132) by rotating overnight at 4 °C. 10% of the soluble fraction was saved as the input sample. The beads were washed three times with IP Buffer (25 mM Tris-HCl (pH 7.5), 10% glycerol, 1.5 mM DTT, cOmplete, EDTA-free protease inhibitor cocktail tablets (1 tablet per 50 ml buffer), 0.25% NP-40, 150 mM NaCl) before boiling for 5 min in 30 µl sample buffer to release pulled-down proteins. 40 µg input and 10 µl pull-downs were analysed by western blot with anti-GST (Santa Cruz 459) and anti-FLAG-HRP (Sigma A8592) antibodies.

C. elegans strains and methods

Strains were maintained using standard techniques on nematode growth medium agarose plates supplemented with nystatin. N2 (Bristol) wild type and *rfs-1(ok1372)* and *helq-1(tm2134)* mutant strains were as described previously (Ward et al., 2007; Ward et al., 2010). A mutant allele in the open reading frame R01H10.5, *rip-1(tm2948)*, was obtained from the Japanese National BioResource Project for *C. elegans*, which is a 668 bp deletion between nucleotides 12202 and 12870 in cosmid R01H10 that completely removes exons 2 and 3 and generates a frameshift that introduces a premature stop codon in exon 4. This allele is predicted to encode a truncated protein of 68 amino acids. The strain was backcrossed to N2 six times before use. The *rip-1(tm2948) helq-1(tm2134)* double mutant was maintained as a *rip-1(tm2948)* heterozygote by balancing with the hT2(gfp) balancer. Homozygote double mutants were selected by picking worms not expressing GFP in the pharynx.

DNA damage sensitivity assays

Nitrogen mustard (HN2, mechlorethamine; Sigma) sensitivity assays were performed in liquid culture by incubating randomly picked young adult animals in 2 ml total volume S Medium, the indicated drug concentration and 50 µl concentrated HB101 bacteria, at room temperature for 19 h on a slow shaker protected from light. Animals were washed in PBS, recovered for 3 h on OP50 seeded

MYOB plates, then transferred individually to single plates and left to lay for 4 h at 20 °C. 12 worms per strain per dose were scored. The parental worms were then removed and the eggs laid counted immediately and after 2 days at 20 °C, and the percentage of embryonic survival scored. Ionizing irradiation was carried out using a caesium-137 source on young adult animals on seeded plates. Plates were incubated for 22 h at 20 °C before animals were transferred individually to single plates and scored as for HN2.

Immunofluorescence

Randomly picked gravid adult hermaphrodites were treated with nitrogen mustard (HN2, 200 μM) and cisplatin (CDDP, 180 μM) for 19 h in liquid culture and hydroxyurea (HU, 40 mM) on OP50 seeded MYOB plates containing HU for 16 h. Ionizing irradiation and UV-C (254 nm) treatment were performed on seeded plates. After treatment, animals were transferred to fresh seeded plates and allowed to recover (HN2, 16 h; CDDP, 18 h; HU, 16 h; IR, 4 h; UV-C, 2h). Worms were washed twice in PBS, transferred to poly-L-lysine coated slides and germlines dissected. Germlines were fixed in 4% paraformaldehyde for 10 min, permeabilized in TBSBT (1X TBS, 0.5% BSA, 0.1% Triton X-100) for 20 min then washed 3 x 30 min with TBSB (1X TBS, 0.5% BSA). Germlines were stained with a rabbit anti-RAD-51 antibody (gift of Anton Gartner) diluted 1:500 in TBSB overnight at 4 °C in a humid chamber. Germlines were washed 3 x 30 min with TBSB, then incubated with secondary antibodies (anti-rabbit Cy3 (Sigma) or anti-rabbit Alexa488 (Life Technologies)) diluted 1:10000 in TBSB for 2 h at room temperature in a dark humid chamber. The germ lines were washed 1 x 30 min with TBSB, stained 5 min with DAPI (1 μg/ml in TBSB), washed 3 x 30 min with TBSB, then mounted with Vectashield. Images were acquired by Deltavision microscopy and analysed as described (Martin et al., 2005). Foci were analysed from 10-20 nuclei from each of 5-25 animals.

Germline apoptosis assays

Randomly picked young adult worms were incubated in 33 μM SYTO12 dye (Molecular Probes) in 50 μl M9 for 3 h at 20 °C protected from the light. Worms were then washed with PBS, incubated for 1 h on OP50 seeded MYOB plates in the dark to purge SYTO12-stained bacteria in the gut. Animals were then mounted in 10 mM levamisole on a 4% agarose pad and imaged and scored using an excitation wavelength of 488 nm on an Axioskop2 microscope (Zeiss).

Supplemental References

Frigola, J., Remus, D., Mehanna, A., and Diffley, J.F. (2013). ATPase-dependent quality control of DNA replication origin licensing. Nature. *495*, 339-343.

Senavirathne, G., Jaszczur, M., Auerbach, P.A., Upton, T.G., Chelico, L., Goodman, M.F., and Rueda, D. (2012). Single-stranded DNA Scanning and Deamination by APOBEC3G Cytidine Deaminase at Single Molecule Resolution. J. Biol. Chem. *287*, 15826-15835.

Shahid, T., Soroka, J., Kong, E.H., Malivert, L., McIlwraith, M.J., Pape, T., West, S.C., and Zhang, X. (2014). Structure and mechanism of action of the BRCA2 breast cancer tumor suppressor. Nat. Struct. Mol. Biol. *21*, 962-968.
